# Vaginal *Lactobacillus iners* abundance is associated with outcome in antibiotic treatment of bacterial vaginosis and capable of inhibiting *Gardnerella*

**DOI:** 10.3389/fcimb.2022.1033431

**Published:** 2022-11-22

**Authors:** Rui Zhou, Jingjing Lu, Jun Wang, Bingbing Xiao

**Affiliations:** ^1^ Department of Obstetrics and Gynecology, Peking University First Hospital, Beijing, China; ^2^ Chinese Academy of Science (CAS) Key Laboratory of Pathogenic Microbiology and Immunology, Institute of Microbiology, Chinese Academy of Sciences, Beijing, China; ^3^ University of Chinese Academy of Sciences, Beijing, China

**Keywords:** bacterial vaginosis, *Gardnerella* genomospecies, *Lactobacillus iners*, 16S rRNA sequencing, antimicrobial activity

## Abstract

Bacterial vaginosis is characterized as a polymicrobial dysbiosis with the loss of *Lactobacillus* spp. and growth of multiple anerobic bacteria, including *Gardnerella*, *Prevotella* and *Atopobium* ranked as the top three most abundant. A total of nine *Gardnerella* genomospecies have been identified, yet the association between their distribution or any exact *Lactobacillus* species with BV occurrence or prognosis remains controversial. A total of 308 patients and 62 healthy women who sought annual examinations were recruited, with 130 BV patients and 41 healthy women who met our inclusion criteria finally included. Vaginal samples were used for microscopic examination, 16S rRNA sequencing, bacterial culture and isolation. Isolates of *Gardnerella vaginalis*, *Fannyhessae vaginae* (used to be called *Atopobium vaginae*) and *Lactobacillus iners* were used for competition tests. We found that the relative abundances of *Gardnerella*, *Prevotella* and *Atopobium* were elevated in BV patients compared to healthy people (p<0.0001), yet no significant differences were found among patients with different clinical outcomes (p>0.05). Seven out of nine *Gardnerella* genomospecies were present in both BV patients and healthy women, and the relative abundances of all detected genomospecies were higher in BV patients (p<0.05). Cured patients possessed higher GS03 than intermediate and failed patients (p=0.005, 0.0337). *L. iners* was significantly higher in cured patients than in the other two groups (p=0.0021, p<0.0001), and its ability to inhibit the growth of *G. vaginalis* and *F. vaginae* was validated. In summary, seven *Gardnerella* genomospecies were detected in Chinese BV patients, but no association of its distribution and BV occurrence or prognosis was found. The relative abundance of *L. iners* was higher in cured patients, and its antimicrobial activity against *G. vaginalis* and *F. vaginae* was validated through *in vitro* inhibition experiment. *L. iners* could become a predictive indicator of clinical outcomes of BV patients, and its antimicrobial function might be beneficial to BV patients.

## Introduction

Bacterial vaginosis (BV) is the most common lower genital tract infection, affecting approximately 4-75% of reproductive-aged women internationally (Kenyon et al., 2013; Onderdonk et al., 2016; Abou Chacra et al., 2021). However, the exact etiology of BV still remains unclear. BV is characterized as a dysbiosis of the vaginal microbiome in which the *Lactobacillus* spp. dominant flora is lost (Abou Chacra et al., 2021), accompanied by a significant increase in anaerobic bacteria, including *Gardnerella*, *Atopobium*, *Prevotella*, *Megasphaera*, *Mobiluncus* and so on (Ravel et al., 2011; Muzny et al., 2018; Muzny et al., 2019; Mohankumar et al., 2022). Antibiotics such as metronidazole and clindamycin are recommended for BV treatment, and the short-term cure rate varied from 46.75%-96.20%, but above 70% of women will experience at least one episode of BV recurrence within 12 months (Bostwick et al., 2016; Munoz-Barreno et al., 2021). Recurrent episodes of BV have been demonstrated to be related to a variety of adverse outcomes in gynecology and obstetrics, such as sexually transmitted diseases, cervical cancer, pelvic inflammatory disease, infertility, and premature birth, causing a significant financial burden to the health system and society worldwide (King et al., 2011; Srinivasan et al., 2012; Ravel et al., 2021; Abou Chacra et al., 2021).

In past studies, a variety of anaerobic bacteria have been shown to be closely related to BV. Among these bacteria, *Gardnerella* has attracted special attention, as a series of 16S rRNA sequencing-based techniques have revealed that it could be detected in almost all BV patients, and its presence accounts for the formation of polymicrobial biofilms, which are related to refractory or recurrent BV (Jung et al., 2017; Vestby et al., 2020; Rosca et al., 2022); paradoxically, 40% of healthy women also test positive for such bacteria (Jung et al., 2017). Therefore, whether this species is the contributing pathogen for BV remains debatable (Schwebke et al., 2014; Onderdonk et al., 2016; Morrill et al., 2020). In recent years, researchers have isolated and identified 9 genomospecies of *Gardnerella* through cpn60 gene typing, whole genome sequencing and other methods (Schellenberg et al., 2016; Schellenberg et al., 2017; Hill and Albert, 2019; Vaneechoutte et al., 2019; Qin and Xiao, 2022). Many investigations have focused on identifying the differences in the ability to adhere to vaginal epithelial cells, virulence and drug resistance among genomospecies and the relevance of the distribution of *Gardnerella* genomospecies with the occurrence, symptoms or clinical outcome of BV, but the results lack consistency (Santiago et al., 2011; Onderdonk et al., 2016; Hardy et al., 2017; Hilbert et al., 2017; Deng et al., 2018; Janulaitiene et al., 2018; Ferreira et al., 2021; Khan et al., 2021).

With respect to the normal vaginal microbiome, which is important for the homeostasis of the vaginal environment, studies have also accumulated and concluded that the loss of *Lactobacillus* is an essential part of the progression of BV (Abou Chacra et al., 2021). Based on 16S rRNA sequencing of women across countries and ethnic groups, it is generally accepted that *L. crispatus*, *L. iners*, *L. gasseri* and *L. jensenii* are the four most commonly detected *Lactobacillus* species in the vaginal microbiome (Ravel et al., 2011). Former studies have explored the probiotic effect of different *Lactobacillus* spp. against different pathogens, with *L. crispatus* considered to be the most important species contributing to reproductive health and the combination of *L. gasseri*, *L. jensenii* and *L. acidophilus* might manifest protective effect against dysbiosis of vaginal microbiota (Pacha-Herrera et al., 2022). However, the true role *L. iners* playing in BV progression and prognosis or the restoration of normal vaginal flora remains debatable (Vaneechoutte, 2017; Abou Chacra et al., 2021). Thus, in our study, we profiled the vaginal microbiome in healthy women and BV patients before and after antibiotic treatments and explored the potential contributions of *Gardnerella* and *Lactobacillus* to the treatment outcome of BV at the genomospecies or species level.

## Materials and methods

### Cohort recruitment

A total of 308 premenopausal (18-50), nonpregnant women who came to Peking University First Hospital with major complaints about vulvovaginal discomfort and/or abnormal vaginal discharge and 62 women who underwent annual physical examinations from August 2020 to August 2021 were recruited for our study and a written form of informed consent was collected, approved and supervised by The Ethics Committee of Peking University First Hospital. All participants underwent blood tests, urine tests and cervicovaginal microscopic or PCR tests to rule out infections of HIV, HPV, HSV-2, syphilis, *Chlamydia trachomatis*, *Neisseria gonorrhoeae*, vulvovaginal candidiasis, *Trichomonas vaginalis*, *Ureaplasma urealyticum*, *M. hominis*, urinary tract infections and internal diseases, such as hypertension, diabetes, and hyperlipidemia. Exclusion criteria included pregnancy, diagnosis of any diseases mentioned above, multiple sex partners, history of any intrauterine operations such as hysteroscopy or implantation of intrauterine devices. Patients who were currently in the menstrual period or took oral contraception, any antibiotics whether orally, intravenously or vaginally applied within 30 days or engaged in sexual intercourse within 7 days ahead of sample collection were also excluded from this study. Information of menstrual cycle, last menstruation and reproductive history was also collected by the time of sample collection.

### Sample collection

Three vaginal microbiome samples were collected from the same position in the upper 1/3 of the anterior vaginal wall with vaginal swabs (Becton, Dickinson and Company) during inspection. The first swab was used for DNA extraction and sequencing and immediately stored at -80°C. The second swab was used for Gram staining, microscopic examination and evaluation of biological parameters. The last swab was used for bacterial culture, isolation and purification. The studies involving human participants were reviewed and approved by The Ethics Committee of Peking University First Hospital. All participants signed informed consent in written form for the publication of any potentially identifiable images or data included in this article and agreed to be involved in our follow-up voluntarily.

### Diagnostic procedures and treatment

The presence of BV is diagnosed by the Gram stain-based Nugent score according to Nugent et al. (Nugent et al., 1991) (a score of 0-3 is considered to be normal for BV, 4-6 intermediate status, and 7-10 BV) and Amsel criteria according to Amsel et al (Amsel R Fau - Totten et al., 1983) (BV is diagnosed when at least three of the following criteria are fulfilled: Homogenous, thin, grayish-white vaginal discharge that smoothly coats the vaginal wall, vaginal pH>4.5, release of fishy odor when 10% potassium hydroxide is added, and/or over 20% clue cells present on one saline wet mount). Two experienced technicians were involved in the microscopic examination separately and blinded to each other to ensure the authenticity of the diagnosis. Patients who were diagnosed with BV were prescribed topical 5% metronidazole gel for 5 days. All patients were asked to visit their gynecologist again within one week after completion of their treatment. Another two vaginal swabs were collected following the procedures above. The same diagnostic procedures mentioned above were repeated to confirm the patients’ clinical outcomes: cured (Nugent 0-3), intermediate (Nugent 4-6) and failed (Nugent 7-10).

### Genomic DNA isolation from vaginal samples

The vaginal swab and scraped samples were vortexed and centrifuged for 10 min at 10,000 g to collect the bacterial cells, and the supernatant was discarded. All genomic DNA extractions were performed by using the DNeasy^®^ Power Soil^®^ Pro Kit (Qiagen) following the manufacturer’s instructions.

### 16S rRNA sequencing

A 16S rRNA gene fragment comprising the V3 and V4 hypervariable regions was amplified by using the V3 forward primer 5′-CCTACGGGNBGCASCAG-3′ and the V4 reverse primer 5′-GACTACNVGGGTATCTAATCC-3′. The amplified products were checked and analyzed on a 2% agarose gel. Sequencing was performed using a 250-bp paired-end sequencing protocol on the Illumina NovaSeq6000 platform. Sequence analysis was performed following a previous study (Falony et al., 2016). The sequences were merged using the FLASH program (Magoc and Salzberg, 2011) and subjected to quality filtering using the FASTX-Toolkit (http://hannonlab.cshl.edu/fastx_toolkit/). Chimeras were excluded using the UCHIME command and the ‘GOLD’ database (Edgar et al., 2011). After random rarefication of microbiome sizes to 6555 reads, the taxonomic assignment of reads was determined by RDP classifier (Wang, 2007) to generate the composition matrices at the level of the phylum to the genus (Wang et al., 2007). The 6,555 rarefied reads were also blasted against the 16S rRNA sequences of established *Gardnerella* genomospecies and *Lactobacillus* species (including *L. crispatus*, *L. iners*, *L. jensenii* and *L. gasseri*) to identify the genomospecies *Gardnerella* and *Lactobacillus.* The 16S rRNA sequences of *Gardnerella* and *Lactobacillus* species in NCBI were collected (**Supplementary Table 1**) were distinguishable at the species level (**Supplementary Figure 1**) and were used to build the species level database of *Gardnerella* and *Lactobacillus*. The 6,555 rarefied reads were classified using the BLASTn algorithm against the species level database to identify the species of *Gardnerella* and *Lactobacillus* with identity threshold of >= 99%.

### Bacterial isolation and culture conditions

Vaginal swabs were immediately inoculated onto Colombia blood agar, baked sheep blood agar with kanamycin and vancomycin and MRS broth (BD Difcoä) supplemented with IsoVitale XTM Enrichment (BD BBLTM; 2% v/v) and L-Cys (augmented by L-Gln, with a final concentration of 1.1 mM). All broths mentioned above were securely stored at 4°C until used. The broths were placed into an anerobic environment at 37°C using an AS-580 anaerobic chamber (anaerobic system) with an atmosphere of 5% carbon dioxide, 5% hydrogen, and 90% nitrogen (AirgasO) for 24-48 hours. All bacterial colonies from all broths were picked out, purified and identified through 16S sequencing. *G. vaginalis*, *F. vaginae* and *L. iners* were tittered and maintained on Columbia blood agar.

### Antimicrobial activity evaluation

Purified *Gardnerella vaginalis* and *Fannyhessae vaginae* (used to be classified into *Atopobium vaginae*) strains were spread onto Colombia blood agar and coated on all boards after activation. Agar containing purchased *L. johnsonii* strains was used as positive control and agar containing purified water was used as negative control. Purchased *L. johnsonii* was safely stored in Microbank tubes (ProLab) at -80°C and fully activated before experiment. Agars containing isolated *L. iners*, purchased *L. johnsonii* or water were placed onto broth coated with either *G. vaginalis* (GS01) or *F. vaginae* and cultured under the anaerobic conditions mentioned above. Parallel tests for *L. johnsonii* and *L. iners* were run to ensure the validity of our results. The diameter of the inhibition zone was measured after culturing for 24-48 h.

### Statistical analysis

Statistical analysis of bacterial taxonomic identification was performed using R v4.1.1 software. The vegan package was used to analyses the α-diversity and conduct permutational multivariate analysis of variance (PERMANOVA) (Somerfield et al., 2021) followed in case of significant effects by a constrained canonical analysis of principal coordinates (Anderson, 2003). The Wilcoxon test and Kruskal-Wallis test in the ggpubr package were used to measure the difference in richness, α-diversity and abundance.

## Results

### Cohort description

Of the 370 participants we recruited, 130 BV patients and 41 healthy women were ultimately included in our study. The clinical information of all participants is shown in **Table 1**. After a standard 5-day metronidazole treatment, patients were divided into three groups according to their clinical outcome: 61 patients were cured (46.9%, group cured), 36 patients turned to intermediate BV (27.7%, group intermediate), and 33 patients still had BV (25.4%, group failed). There was no significant difference in age between healthy women and BV patients (38.03 vs. 37.19, p=0.4764, Kruskal-Wallis test). However, statistically significant differences could be seen in both Nugent score (0.58 vs. 7.88, p<0.0001, Kruskal-Wallis test) and pH (4.21 vs. 5.02, p<0.0001, Kruskal-Wallis test) between the two groups. Moreover, no difference was found in reproductive history or proportion of participants in any menstrual period between healthy participants and BV patients (**Table 1**). Furthermore, we analyzed the differences in Nugent score and vaginal pH among the three groups before treatment, and no significant differences were found

**Table 1:** Cohort description.

Mean (95CI%)	Healthy participants	BV patients	P value^b^
	(N=41)	(N=130)	
Age	38.03 (35.09-40.97)	37.19 (35.64-38.74)	
Nugent Score	0.58 (0.31-0.83)	7.88 (7.65-8.12)	<.0001
pH	4.21 (4.07-4.36)	5.02 (4.96-5.07)	<.0001
Infection of Other STI^a^	None		
Menstrual Cycle			
Follicular phase	48.78% (32.81%-64.75%)	46.15% (37.47%-54.84%)	
Period of ovulation	17.07% (5.05%-29.10%)	6.92% (2.50%-11.35%)	
Luteal phase	34.15% (18.99%-49.30%)	46.92% (38.23-55.62%)	
Reproductive history			
G	1.39 (1.07-1.71)	1.45 (1.24-1.67)	
P	0.71 (0.55-0.87)	0.80 (0.69-0.91)	

BV, bacterial vaginosis; STI, sexually transmitter infections.

^a^Other STI include HIV, HPV, HSV-2, syphilis, Chlamydia trachomatis, Neisseria gonorrhoeae, vulvovaginal candidiasis, Trichomonas vaginalis, Ureaplasma urealyticum, M. hominis, and urinary tract infections. ^b^Only statistically significant P value is manifested in the table. Kruskal-Wallis test was used for inter-group comparison.

### BV patients have higher *Gardnerella*, *Prevotella* and *Atopobium*

**Figure 1:**
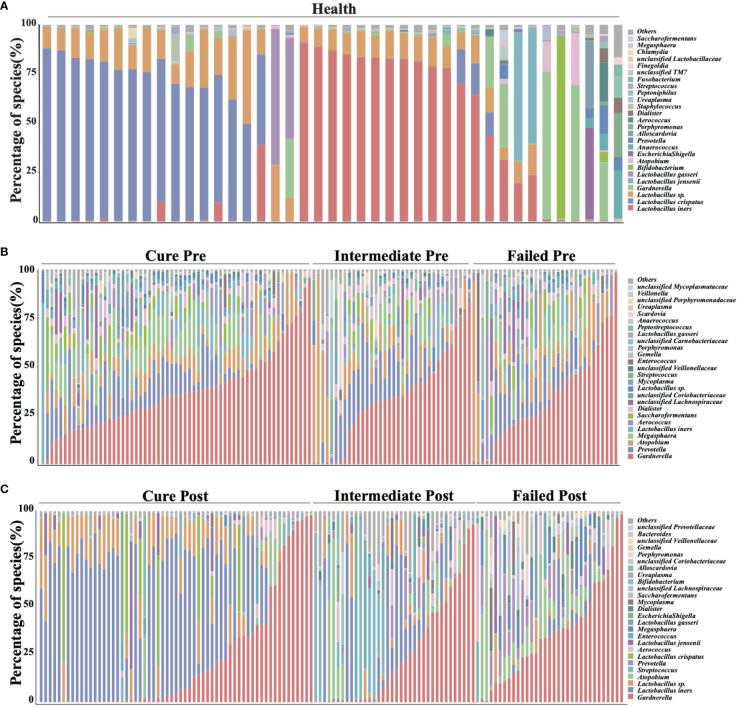
Vaginal microbiome composition of studied cohort. This figure manifests the top 26 most abundant bacteria in participants’ vaginal microbiome, organisms ranked 27 and below are all categorized into label “others”. **(A)** shows the vaginal microbiome of healthy participants, with **(B)** showing BV patients pretreatment and **(C)** showing posttreatment.

**Figure 2:**
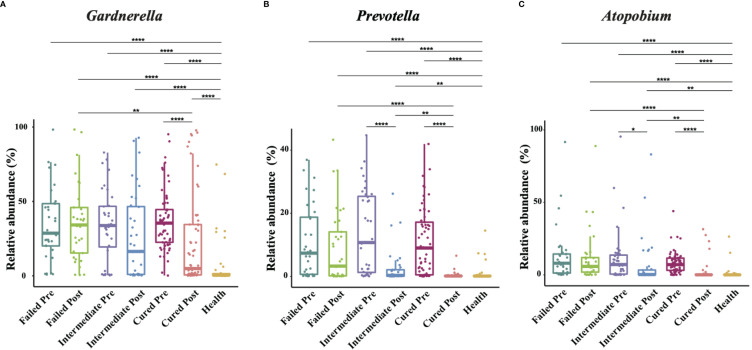
Comparison of *Gardnerella, Prevotella* and *Atopobium* abundance in different groups. Intergroup comparison of *Gardnerella* **(A)**, *Prevotella* **(B)** and *Atopobium* **(C)** relative abundance. Only statistically significant *P* value that has clinical meaning is marked in the graph. Significance is exhibited as: *: p<0.05; **: p<0.01; ***: p<0.001; ****: p<0.0001; Wilcoxon test for pairwise comparison between pre- and posttreatment and Kruskal-Wallis test for comparisons among different groups.

We analyzed all vaginal microbiota through 16S rRNA sequencing (**Figure 1**). The results reveal that *Lactobacillus.* spp. are the most dominant species in healthy women (78.95%), while 16 (39%) are *L. crispatus* dominant and 14 are *L. iners* dominant (34%). The average relative abundance of *L. crispatus* among those who were *L. crispatus* dominant was 71.77%, while average *L. iners* abundance was 81.27% when *L. iners* was dominant. In contrast, BV-related bacterial species are the most prevalent taxa in BV patients before taking any medications: Gardnerella spp. (35.61%) *Prevotella* (11.66%) and *Atopobium* (10.69%) were among the top three highest relative abundances in BV patients (**Table 2**). In terms of relative abundance before treatment, all three bacteria were statistically higher than those in healthy women (p<0.0001, Kruskal-Wallis test), while the relative abundance of each bacterium was similar among groups before the application of metronidazole and not statistically significant (p>0.99, Kruskal-Wallis test) (**Figure 2**).

**Table 2:** Relative abundance of top 10 most abundant bacteria at genus level.

	All participants_(N=171)_			Group cured _(N=61)_^a^			Group intermediate_(N=36)_^b^			Group failed _(N=33)_^c^		
Genus	Healthy	BV patients	*P* value^d^	Pretreatment	Posttreatment	*P* value^d^	Pretreatment	Posttreatment	*P* value^d^	Pretreatment	Posttreatment	*P* value^d^
*Relative abundance (%) of each genus, mean (95%CI)*												
*Lactobacillus*	78.95 (68.00-89.89)	7.66 (5.16-10.15)	<.0001	10.06 (5.96-14.16)	67.33 (57.78-77.09)	.0048	4.96 (1.53-8.38)	11.46 (4.29-18.64)	.231	6.23 (0.92-11.54)	23.23 (12.06-34.39)	.0761
*Gardnerella*	7.12 (1.70-12.54)	35.61 (31.73-39.50)	<.0001	37.21 (31.93-42.50)	22.32 (14.33-30.30)	<.0001	34.08 (26.07-42.09)	25.74 (15.97-35.51)	.0946	34.38 (25.73-43.03)	36.01 (26.72-45.30)	.980
*Prevotella*	0.98 (0.10-1.85)	11.66 (9.64-13.68)	<.0001	10.73 (7.97-13.48)	0.33 (0.10-0.56)	<.0001	14.07 (9.61-18.52)	2.69 (0.78-4.60)	<.0001	10.74 (6.69-14.78)	9.09 (4.99-13.19)	.1482
*Atopobium*	1.25 (-0.23-2.73)	10.69 (8.08-13.31)	<.0001	8.27 (6.24-10.29)	1.95 (0.29-3.61)	<.0001	12.35 (5.85-18.85)	7.02 (1.33-12.71)	.0103	13.29 (6.54-20.04)	12.07 (5.70-18.45)	.0657
*Megasphaera*	0.12 (-0.06-0.30)	7.56 (6.04-9.08)	<.0001	9.08 (6.74-11.42)	0.10 (0.045-0.16)	<.0001	5.74 (2.95-8.53)	0.61 (-0.05-1.27)	.0005	6.78 (3.81-9.76)	6.85 (3.56-10.15)	.7341
*Aerococcus*	0.60 (-0.40-1.59)	3.77 (2.35-5.18)	<.0001	3.63 (2.02-5.24)	0.93 (0.36-1.50)	<.0001	3.14 (0.81-5.48)	3.38 (1.16-5.60)	.6507	4.70 (0.52-8.89)	4.76 (0.60-8.91)	.7609
*Saccharofermentans*	0.12 (-0.096-0.33)	3.26 (2.27-4.25)	<.0001	4.09 (2.40-5.79)	0.04 (0.017-0.060)	<.0001	2.06 (0.58-3.54)	0.15 (0.02-0.29)	.0013	3.06 (1.23-4.90)	2.95 (0.91-4.99)	.8656
*Dialister*	0.30 (-1.96-2.55)	2.95 (2.52-3.39)	<.0001	3.33 (-6.40-13.05)	0.09 (-0.50-0.67)	<.0001	2.90 (-7.85-13.66)	0.81 (-7.53-9.14)	<.0001	2.93 (1.23-4.62)	2.82 (1.20-4.44)	.5207
*Streptococcus*	0.31 (0.045-0.57)	0.95 (0.23-1.68)	<.0001	0.20 (0.019-0.39)	1.13 (0.15-2.11)	.1953	2.28 (0.098-4.47)	13.60 (4.64-22.56)	.0123	0.86 (-0.71-2.43)	1.14 (-0.75-3.04)	.5391
*Enterococcus*	0.02 (0.004-0.05)	0.82 (-0.10-1.74)	.290	0.12 (0.030-0.320	0.00 (0.00-0.002)	.0143	0.08 (0.0015-0.16)	0.00 (0.00-0.01)	.009	1.99 (-1.46-5.44)	1.15 (-0.97-3.28)	.7718

BV, bacterial vaginosis.

^a^ “Group Cured” was defined as patients whose Nugent score were lowered to 0-3 after metronidazole treatment. ^b^ “Group Intermediate” was defined as patients whose Nugent score were changed to 4-6 after metronidazole treatment. ^c^ “Group Failed” was defined as patients whose Nugent score remained at 7-10 after metronidazole treatment. ^d^ Wilcoxon test was used for comparison between these two groups.

We then analyzed the vaginal microbiome in BV patients after metronidazole treatment and found significant differences in microbiome composition among patients in different clinical outcome groups. In cured patients (group cured), the relative abundance of all three BV-associated bacteria significantly decreased (p<0.0001, Wilcoxon test), yet the relative abundance of Gardnerella spp. was still higher than that in the healthy cohort (p<0.0001, Wilcoxon test). The relative abundance of both *Atopobium* and *Prevotella* decreased posttreatment in the intermediate group (group intermediate, p=0.0103, p<0.0001, Wilcoxon test), but the abundance of Gardnerella spp. did not change significantly (p=0.0946, Wilcoxon test). In contrast to the two groups with improvement, no significant decrease in any bacteria was detected in patients without improvement (failed group). Intergroup comparison shows that patients in the cured group had a lower relative abundance of Gardnerella spp., *Atopobium* and *Prevotella* than the failed group (p=0.0009, p<0.0001, p<0.0001, Kruskal-Wallis test) and a lower relative abundance of *Atopobium* and *Prevotella* than the intermediate group (p=0.0002, p=0.0038, Kruskal-Wallis test). Meanwhile, the intermediate group contained a lower relative abundance of *Atopobium* and *Prevotella* than the failed group (p=0.0022, p=0.0254, Kruskal-Wallis test), but the relative abundance of Gardnerella spp. showed no significant differences between the cured and intermediate groups or between the intermediate and failed groups (**Figure 2**).

Furthermore, we analyzed the α-diversity (Shannon index and Chao1 index) in participants’ vaginal microbiota (**Supplementary Figure 3**). We found that, α-diversity in all BV patients was significantly higher compared to healthy women (p<0.05), yet no statistical differences were noticed among groups with different clinical outcomes pretreatment. After metronidazole treatment, α-diversity was reduced in group cured and significantly higher than group intermediate and group failed, but was still significantly higher compared to healthy women. No statistical changes of α-diversity were noticed in either group intermediate or group failed after treatment. Moreover, no statistical differences were shown between group intermediate and group failed post-treatment.

### Cured patients possessed higher GS03 pretreatment

**Figure 3:**
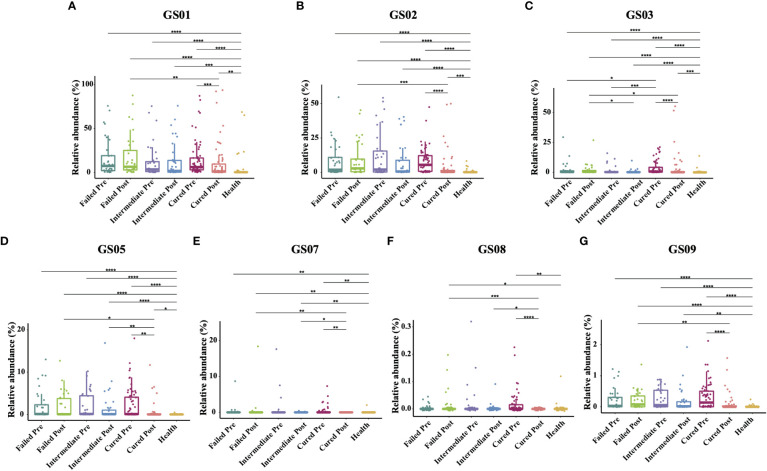
Comparison of *Gardnerella* genomospecies abundance in different groups. Intergroup comparison of the relative abundance of each *Gardnerella* genomospecies: GS01 **(A)**, GS02 **(B)**, GS03 **(C)**, GS05 **(D)**, GS07 **(E)**, GS08 **(F)** and GS09 **(G)**. Only statistically significant *P* value that has clinical meaning is marked in the graph. Significance is exhibited as: *: p<0.05; **: p<0.01; ***: p<0.001; ****: p<0.0001; Wilcoxon test for pairwise comparison between pre- and posttreatment and Kruskal-Wallis test for comparisons among different groups.

Since former studies have recognized nine different *Gardnerella* genomospecies *via* whole genome sequencing, only seven genomospecies have been detected in our specimen, namely, GS01, GS02, GS03, GS05, GS07, GS08 and GS09, with decreasing abundance. Each detected *Gardnerella* genomospecies was increased in BV patients compared to healthy women pretreatment (p<0.01, Kruskal-Wallis test) (**Table 3**). When comparing groups of patients with different treatment outcomes, we found that only the abundance of GS03 in the cured group was significantly higher than that in the intermediate group and the group that failed before treatment (p=0.005, 0.0337, Kruskal-Wallis test), while the abundances of other genomospecies showed no significant differences among groups (**Figure 3**).

**Table 3:** Relative abundance of each *Gardnerella* genomospecies in each group.

	All participants _(N=171)_			Group cured _(N=61)_^a^			Group intermediate _(N=36)_^b^			Group failed _(N=33)_^c^		
Genomospecies	Healthy	BV patients	*P* value^d^	Pretreatment	Posttreatment	*P* value^d^	Pretreatment	Posttreatment	*P* value^d^	Pretreatment	Posttreatment	*P* value^d^
*Relative abundance (%) of each genus, mean (95%CI)*												
*GS01*	5.20 (0.37-10.02)	14.7 (11.14-18.26)	<.0001	15.22 (9.89-20.54)	11.03 (5.47-16.59)	.0002	12.17 (5.63-18.71)	12.94 (6.09-19.79)	.3272	16.52 (8.84-24.19)	17.95 (9.41-26.48)	.8878
*GS02*	0.65 (0.14-1.16)	9.16 (6.98-11.33)	<.0001	7.94 (5.54-10.34)	3.73 (1.13-6.32)	<.0001	11.66 (6.00-17.31)	7.56 (3.18-11.95)	.2112	8.64 (4.33-12.95)	9.04 (4.56-13.53)	.9234
*GS03*	0.54 (-0.16-1.23)	2.83 (1.89-3.76)	<.0001	3.84 (2.30-5.37)	3.45 (0.77-6.13)	<.0001	1.62 (0.37-2.86)	0.68 (0.10-1.26)	.5614	2.30 (0.30-4.30)	2.16 (0.42-3.89)	.8374
*GS05*	0.02 (0.00-0.03)	2.17 (1.56-2.77)	<.0001	2.35 (1.37-3.33)	0.52 (0.03-1.01)	.0012	1.95 (0.93-2.96)	1.48 (0.35-2.62)	.5835	2.07 (0.84-3.30)	1.93 (0.84-3.01)	.657
*GS07*	0.05 (-0.05-0.15)	0.52 (0.16-0.87)	.0053	0.42 (0.10-0.74)	0.00 (0.00-0.00)	.0053	0.85 (-0.24-1.93)	0.03 (0.00-0.05)	.6835	0.33 (-0.20-0.87)	0.62 (-0.05-1.75)	.7967
*GS08*	0.00 (0.00-0.00)	0.01 (0.00-0.02)	.0189	0.02 (0.00-0.03)	0.00 (0.00-0.00)	<.0001	0.02 (-0.02-0.04)	0.02 (-0.02-0.04)	.2555	0.00 (0.00-0.00)	0.02 (0.00-0.03)	.6641
*GS09*	0.01 (-0.01-0.02)	0.28 (0.22-0.35)	<.0001	0.32 (0.21-0.43)	0.09 (0.02-0.16)	<.0001	0.27 (0.16-0.37)	0.18 (0.05-0.31)	.0954	0.24 (0.11-0.37)	0.21 (0.10-0.31)	.9843

BV, bacterial vaginosis; GS, genomospecies.

^a^ “Group Cured” was defined as patients whose Nugent score were lowered to 0-3 after metronidazole treatment. ^b^ “Group Intermediate” was defined as patients whose Nugent score were changed to 4-6 after metronidazole treatment. ^c^ “Group Failed” was defined as patients whose Nugent score remained at 7-10 after metronidazole treatment. ^d^ Wilcoxon test was used for comparison between these two groups.

With respect to treatment outcome, in the cured group, the relative abundance of every *Gardnerella* genomospecies was decreased posttreatment (p<0.05, Wilcoxon test), but only the relative abundance of GS07, GS08 and GS09 was restored to levels similar to those of healthy individuals (**Figure 3**). In the intermediate group or the failed group, no significant changes were found in any genomospecies before and after treatment (p>0.05, Wilcoxon test). Further analysis showed that the relative abundance of GS05, GS07 and GS08 was lower in the cured group than in the intermediate group, and all genomospecies were significantly lower than in the failed group. Between the intermediate group and the failed group, only GS03 showed significant differences (p=0.0265, Kruskal-Wallis test) (**Figure 3**).

### Higher *L. iners* is associated with a positive outcome of BV treatment

**Figure 4:**
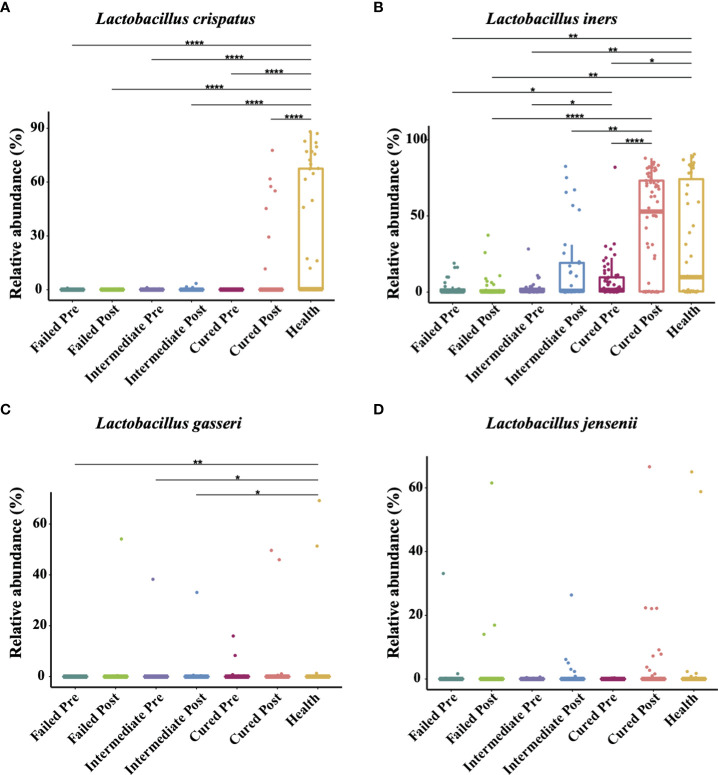
Comparison of four *Lactobacillus* species abundance in different groups. Intergroup comparison of the relative abundance of the four most abundant *Lactobacillus* species: *L. crispatus* **(A)**, *L. iner*s **(B)**, *L. gasseri* **(C)** and *L. jensenii* **(D)**. Only statistically significant *P* value that has clinical meaning is marked in the graph. Significance is exhibited as: *: p<0.05; **: p<0.01; ***: p<0.001; ****: p<0.0001; Wilcoxon test for pairwise comparison between pre- and posttreatment and Kruskal-Wallis test for comparisons among different groups.

The four most commonly observed *Lactobacillus* species in reproductive-aged women are *L. crispatus*, *L. iners*, *L. gasseri* and *L. jensenii;* we specifically allocated the sequences to the four species with a stringent similarity threshold (99%). In the results, we found *L. iners* to be the highest in terms of abundance in healthy individuals, with *L. crispatus*, *L. jensenii* in decreasing order and *L. gasseri* having the lowest proportion. The relative abundance of *Lactobacillus* spp. in BV patients was overall significantly lower than that in healthy group pre-treatment, but in terms of species, only *L. crispatus* and *L. iners* were significantly different among BV patients and healthy women (p<0.0001, p=0.0407, Kruskal-Wallis test) (**Table 4**). We also discovered that even though the relative abundance of *Lactobacillus* spp. in total among the three groups of BV patients was similar before treatment, but the proportion of *L. iners* was higher in the cured group than in the intermediate and failed pretreatment groups (p=0.0021, p<0.0001, Kruskal-Wallis test), while it was not significantly different between the intermediate and failed groups (p>0.9999, Kruskal-Wallis test) (**Figure 4**).

**Table 4:** Relative abundance of four *Lactobacillus* species in each group.

	All participants _(N=171)_			Group cured _(N=61)_^a^			Group intermediate _(N=36)_^b^			Group failed _(N=33)_^c^		
Species	Healthy	BV patients	*P* value^d^	Pretreatment	Posttreatment	*P* value^d^	Pretreatment	Posttreatment	*P* value^d^	Pretreatment	Posttreatment	*P* value^d^
Relative abundance (%) of each genus, mean (95%CI)												
*L. crispatus*	29.21 (18.01-40.41)	0.08 (0.06-0.11)	<.0001	0.069 (0.051-0.087)	5.67 (1.25-10.09)	.4657	0.11 (0.041-0.18)	0.22 (0.0012-0.44)	.4011	0.08 (0.024-0.14)	0.081 (0.047-0.11)	.2359
*L. iners*	30.33 (18.69-41.41)	4.83 (3.12-6.54)	.0288	7.31 (3.89-10.64)	43.71 (34.88-52.54)	.0007	2.51 (0.74-4.27)	15.65 (7.22-24.09)	.0989	2.86 (1.01-4.72)	3.49 (0.68-6.30)	.6350
*L. gasseri*	2.99 (-1.20-7.19)	0.51 (-0.14-1.15)	.0288	0.44 (-0.16-1.04)	1.64 (-0.60-3.87)	.596	1.08 (-1.08-3.23)	0.95 (-0.91-2.81)	.9237	0.01 (0.0025-0.025)	1.67 (-1.67-5.01)	.3355
*L. jensenii*	3.17 (-1.09-7.43)	0.31 (-0.20-0.82)	.2024	0.03 (0.014-0.047)	2.79 (0.25-5.32)	.8006	0.064 (0.018-0.11)	1.23 (-0.30-2.77)	.7226	1.08 (-0.96-3.12)	2.82 (-1.15-6.79)	.9946

BV, bacterial vaginosis.

^a^ “Group Cured” was defined as patients whose Nugent score were lowered to 0-3 after metronidazole treatment. ^b^ “Group Intermediate” was defined as patients whose Nugent score were changed to 4-6 after metronidazole treatment. ^c^ “Group Failed” was defined as patients whose Nugent score remained at 7-10 after metronidazole treatment. ^d^ Wilcoxon test was used for comparison between these two groups.

In addition, we found that *Lactobacillus* spp. abundance in total was restored only in the group cured after being treated with metronidazole (p=0.0048, Wilcoxon test), while other two groups showed no signs of *Lactobacillus* spp. restoration. But at the species level, only *L. iners* showed a significant difference (p=0.0007, Wilcoxon test); thus, it was the most affected species. After metronidazole treatment, only the relative abundance of *L. iners* was significantly different among treatment outcome groups, as the cured group possessed a higher *L. iners* relative abundance than the intermediate and failed groups (p=0.02, p=0.0274, Kruskal-Wallis test). No difference was found between the intermediate group and the failed group with regard to any other *Lactobacillus* species abundance (p>0.05, Kruskal-Wallis test) (**Figure 4**).

### *Lactobacillus iners* inhibits GS01 and Fennyhessae vaginae *in vitro*

As our results indicate that higher *L. iners* is associated with a positive outcome of BV treatment, we examined whether *L. iners* possessed antimicrobial ability against BV related bacteria. We co-cultured clinically isolated *L. iners* with GS01 or *F. vaginae* and used *L. johnsonii*, which has been reported to be capable of inhibiting the growth of a series of pathogens, as a positive control and agar containing purified water as negative control. We found that after culturing for 24-48 h, inhibition zones were manifested in all parallel tests cocultured with GS01 or *F. vaginae*, indicating the inhibitory effect of *L. iners* against the growth of GS01 and *F. vaginae* in the *in vitro* coculture system, yet system of negative control showed no sign of antimicrobial abilities. (**Supplementary Figure 4**).

## Discussions

Our study shows that *L. iners* (30.33%), *L. crispatus* (29.21%) and *Gardnerella* (7.12%) ranked the top three most prevalent bacteria in healthy Chinese women, with *Gardnerella* (37.12%), *Prevotella* (10.73%) and *Atopobium* (8.72%) ranked as the top three in BV patients, but no such correlation between the relative abundance of *Gardnerella*, *Atopobium* or *Prevotella* and clinical outcomes was found. Interestingly, even though the amount of healthy individuals dominant by *L. crispatus* was slightly higher than those dominant by *L. iners* (16 vs 14), we noticed that the average relative abundance of *L. crispatus* was even mildly lower than *L. iners* (29.21% vs 30.33%). This was due to the average abundance of dominant *Lactobacillus* species was different. It seems that when *L. iners* is the dominant species in vaginal flora, it occupies more ecological niche than *L. crispatus*. Our study also showed that BV patients had higher richness and diversity compared to healthy women pretreatment which is in consistence with former studies. The reduction of diversity in group cured after treatment infers the success of treatment, yet the richness is still significantly higher than healthy women indicates that further therapeutic procedures might be required to fully restore the normal vaginal microbiota.

Former studies have demonstrated that different genomospecies of Gardnerella spp. manifest diverse characteristics such as virulent factors, adherent abilities, antibiotics resistance, etc. Although lacking explicit conclusions, studies based on cohorts from other regions and ethnicities, utilizing the concept of “*Gardnerella* clades” based on cpn60 sequencing reported that different structures of the *Gardnerella* clades are related to BV, as certain genomospecies being more abundant or positive in BV patients, while others are not (Numanović et al., 2008; Vodstrcil et al., 2017). In our study, we brought former studies to a further level as we analyzed *Gardnerella* genomospecies which were classified by whole-genome sequencing. We detected seven out of nine genomospecies in Chinese women, with the absence of GS04 and GS06 and all detected genomospecies were presented in both BV patients and healthy people. Furthermore, GS01, GS02, and GS03 ranked the top three most prevalent and GS08 the least in both BV patients and healthy people. No genomospecies are thought to be specifically related to BV, as the relative abundance of all genomospecies is significantly higher in BV patients than in healthy women.

A previous study reported an association between a high abundance of certain *Gardnerella* genomospecies or a combination of several genomospecies with BV clinical outcomes (Harwich et al., 2010; Hilbert et al., 2017; Faught and Reyes, 2019; Hill and Albert, 2019; Shipitsyna et al., 2019), and coinfection of GS03 and GS04 was thought to be related to negative clinical outcomes based on a cohort of recurrent BV patients (Turner et al., 2021; Qin and Xiao, 2022). In contrary to former studies, our study found that the relative abundance of GS03 was even higher in cured patients. However, whether GS03 relative abundance is associated with better clinical outcomes might remain controversial, as in our study, GS01 is the most prevalent *Gardnerella* genomospecies and former studies have shown that GS01 is less resistant to metronidazole compared to GS03 (35% vs 100%). GS03 only made up 3.84% of the whole bacterial taxa which was approximately 1/5 of the most abundant genomospecies GS01(14.7%) and it’s difficult to determine whether this small amount of GS03 is able to shift the clinical outcomes of BV patients. Therefore, we propose that GS01 instead of GS03 might be the most important genomospecies affecting BV clinical outcomes.

At the same time, our study noticed that patients with more *L. iners* before treatment might have a better clinical outcome. This is a notable finding, as it indicates that *L. iners* may be an innovative indicator for BV clinical outcomes, in contrast to previous findings that it might be an opportunistic pathogen (Lee et al., 2020). Different from other *Lactobacillus* species mentioned in this article, *L. iners* shows unique metabolic and genomic characteristics, and its protective function is questionable compared to other *Lactobacilli* (France et al., 2016; France et al., 2020; Bloom et al., 2022; France et al., 2022). Its production of hydrogen peroxide and D-lactic acid is lower, and the inerolysin it secretes is thought to be a cholesterol-dependent cytotoxin that is homogenous to vaginolysin and expressed by several BV-associated bacteria (Zhou et al., 2010; Pleckaityte, 2019; Ragaliauskas et al., 2019; Zheng et al., 2021). It has been acknowledged that metronidazole instantly reduces the load of vaginal microbiota, and *L. iners* becomes the dominant species (Lehtoranta et al., 2020; Verwijs et al., 2020; Armstrong et al., 2022), but this kind of structure is unstable and has the potential to lead to BV recurrence (France et al., 2022). Although lacking consistent conclusions, *L. iners* is considered to be a “foe” instead of a “friend” (Hill and Albert, 2020; Zheng et al., 2021; Novak et al., 2022). However, our study proposed a novel point of view, as we found that the cured patients’ microbiome had more *L. iners*. Our *in vitro* experiments also validated the inhibitory effect of *L. iners* against *G. vaginalis* and *F. vaginae*. We assume that when *L. iners* is higher at the time of treatment, its antimicrobial abilities might facilitate the therapy of BV, as it is resistant to metronidazole and able to scavenge pathogens simultaneously. Furthermore, considering that *L. iners* is capable of synthesizing L-lactic acid and a small amount of D-lactic acid (Vaneechoutte, 2017), we hypothesized that the restoration of *L. iners* after treatment might be crucial for the recovery of other *Lactobacillus* by maintaining an acidic environment and countering the growth of BV-associated bacteria.

This study is the first attempt to describe the distribution of *Gardnerella* genomospecies in Chinese women to determine its relationship with the clinical outcomes of BV patients. Moreover, we also validated the inhibitory effect of *L. iners* against *G. vaginalis* and *F. vaginae* through coculture experiments. Though with limited sample size and restricted experimental conditions, our conclusion could be more general with more incorporated participants. Nonetheless, our *in vitro* test is only preliminary in explaining the correlation of *L. iners* with BV clinical outcomes. In future studies, more experiments and animal models are needed to reveal the mechanism underneath the relation between *L. iners* and BV clinical outcomes and more participants are required to better represent the *Gardnerella* genomospecies distribution in China.

## Conclusion

Our research found seven *Gardnerella* genomospecies and revealed that Chinese women and cured patients possessed higher GS03 and *L. iners* pretreatment and validated the inhibitory effect of *L. iners* against the growth of *Gardnerella vaginalis* and *Fannyhessae vaginae.* Finally, we suggest gynecologists have a better understanding of the vaginal microbiota of BV patients pretreatment to improve their overall health and that *L. iners* might become an innovative biomarker for BV treatment outcomes.

## Data availability statement

The datasets presented in this study can be found in online repositories. The names of the repository/repositories and accession number(s) can be found below: https://nmdc.cn/resource/attachment/detail/NMDCX0000148.

## Ethics statement

The studies involving human participants were reviewed and approved by The Ethics Committee of Peking University First Hospital (2020[083]-001). The patients/participants provided their written informed consent to participate in this study.

## Author contributions

BX and JW designed the project. BX collected samples. RZ and JL ran the experiment procedure. All authors participated in data analysis, writing and discussing the contents of this article and approved the submitted version.

## Funding

This present work was funded by the grants of the National Natural Science Foundation of China (81971342) and the National Key Research and Development Program of China (2021YFC2301000).

## Acknowledgments

We thank Dai ZHANG, Jiahuizi GAO and Hanyu QIN helped with collecting samples and diagnostic procedures. We thank all participants who attended this study.

## Conflict of interest

The authors declare that the research was conducted in the absence of any commercial or financial relationships that could be construed as a potential conflict of interest.

## Publisher’s note

All claims expressed in this article are solely those of the authors and do not necessarily represent those of their affiliated organizations, or those of the publisher, the editors and the reviewers. Any product that may be evaluated in this article, or claim that may be made by its manufacturer, is not guaranteed or endorsed by the publisher.
