# Supplementary material for: Vaginal Lactobacillus iners abundance is associated with outcome in antibiotic treatment of bacterial vaginosis and capable of inhibiting Gardnerella
Source: Front Cell Infect Microbiol. 2022 Nov 22;12:1033431. doi: 10.3389/fcimb.2022.1033431 (PMC9723143; doi:10.3389/fcimb.2022.1033431)
Supplement: Supplementary file 1 [file DataSheet_1.pdf]

[Supplementary Figure 1] Phylogenetic trees of *Gardnerella* genomospecies and *Lactobacillus* species

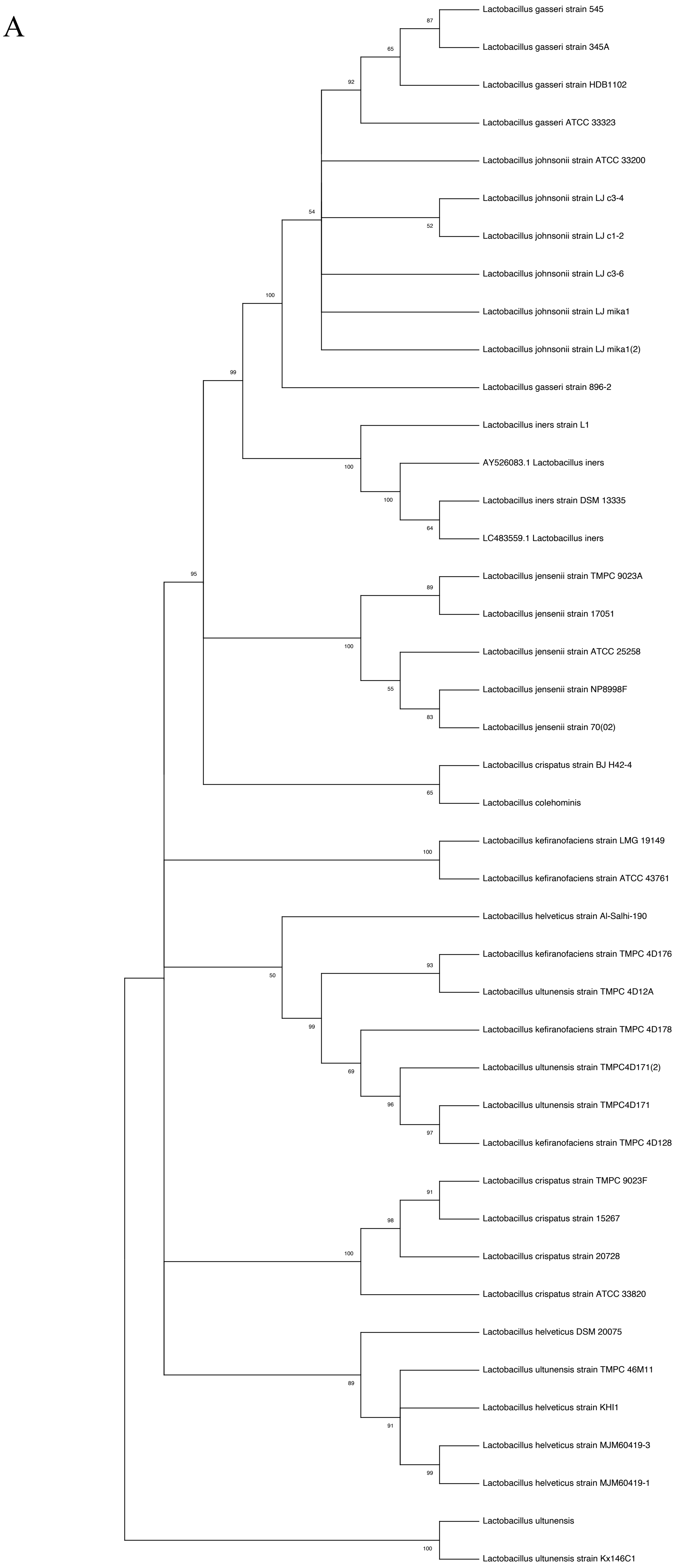

B

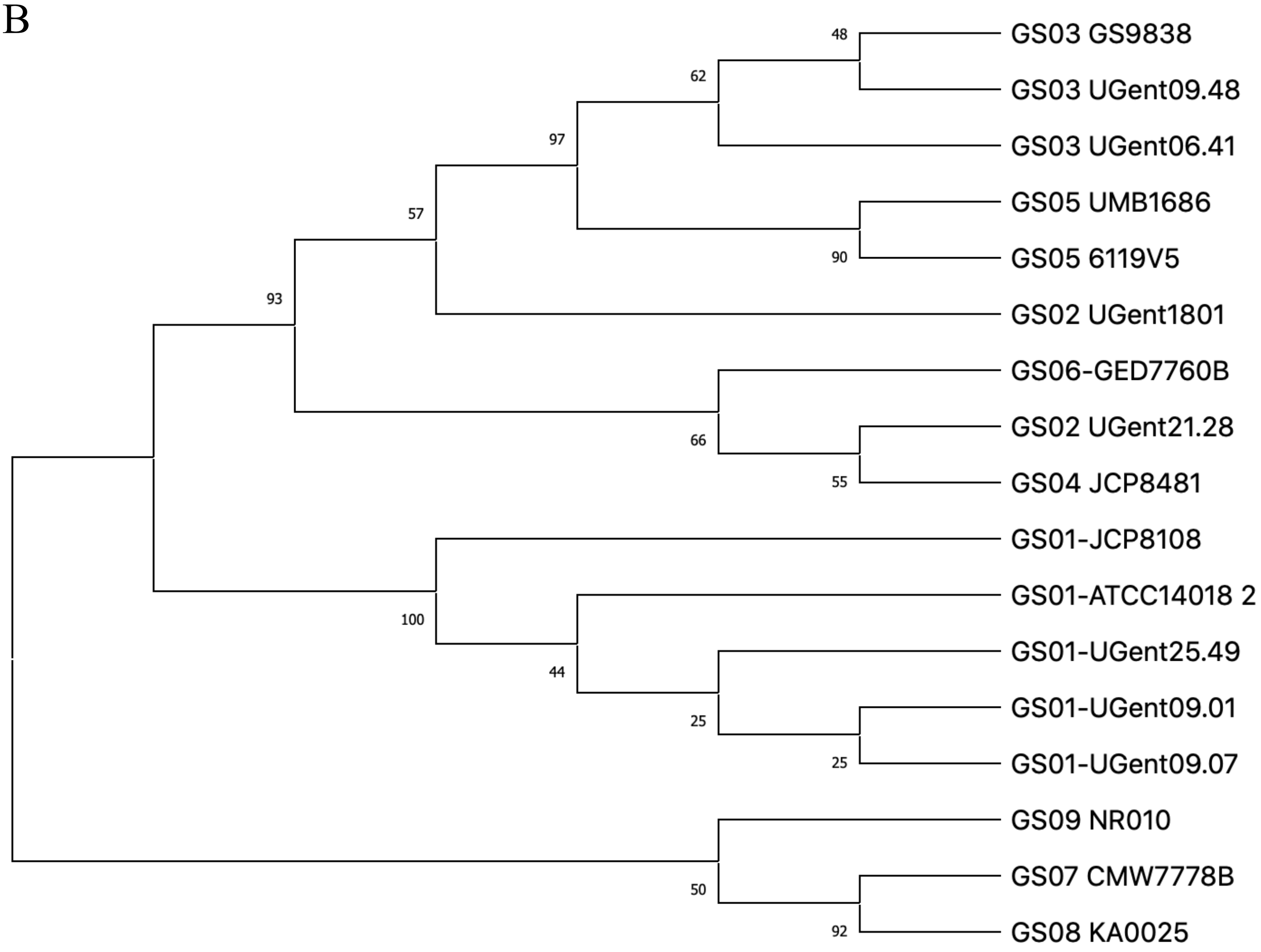
GS03 GS9838
GS03 UGent09.48
GS03 UGent06.41
GS05 UMB1686
GS05 6119V5
GS02 UGent1801
GS06-GED7760B
GS02 UGent21.28
GS04 JCP8481
GS01-JCP8108
GS01-ATCC14018 2
GS01-UGent25.49
GS01-UGent09.01
GS01-UGent09.07
GS09 NR010
GS07 CMW7778B
GS08 KA0025
48
62
97
57
93
90
66
55
100
44
25
25
50
92

[Supplementary Figure 2] Intergroup comparison of Nugent score and vaginal pH

This figure shows the intergroup comparison of Nugent score (Supplementary Figure-2A) and vaginal pH (Supplementary Figure-2B) among Group cured, Group intermediate and Group failed with Kruskal-Wallis test used for statistical analysis. Only statistically significant differences are marked in the figure.

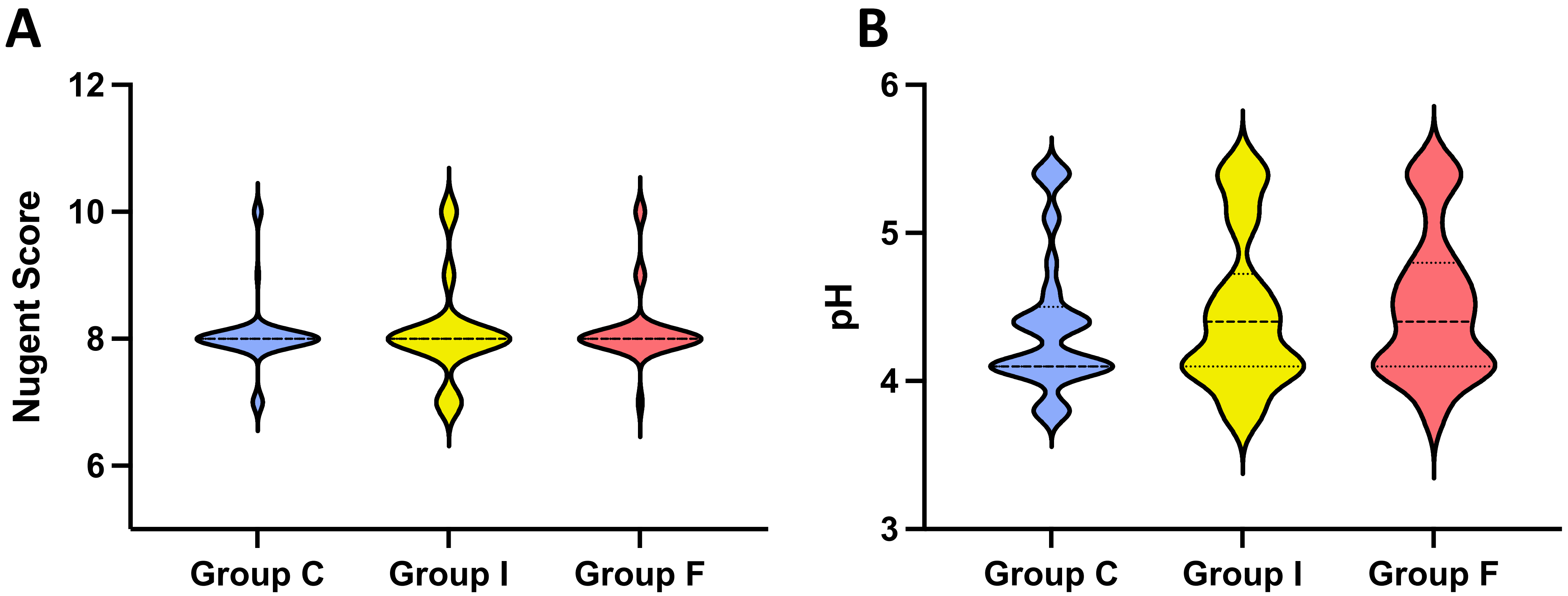

[Supplementary Figure 3] α-diversity analysis of all participants

This figure shows the α-diversity analysis of healthy women and BV patients in each group. Significance is exhibited as: *: p<0.05; **: p<0.01; ***: p<0.001; ****: p<0.0001; Wilcoxon test for pairwise comparison between pre- and posttreatment and Kruskal-Wallis test for comparisons among different groups.

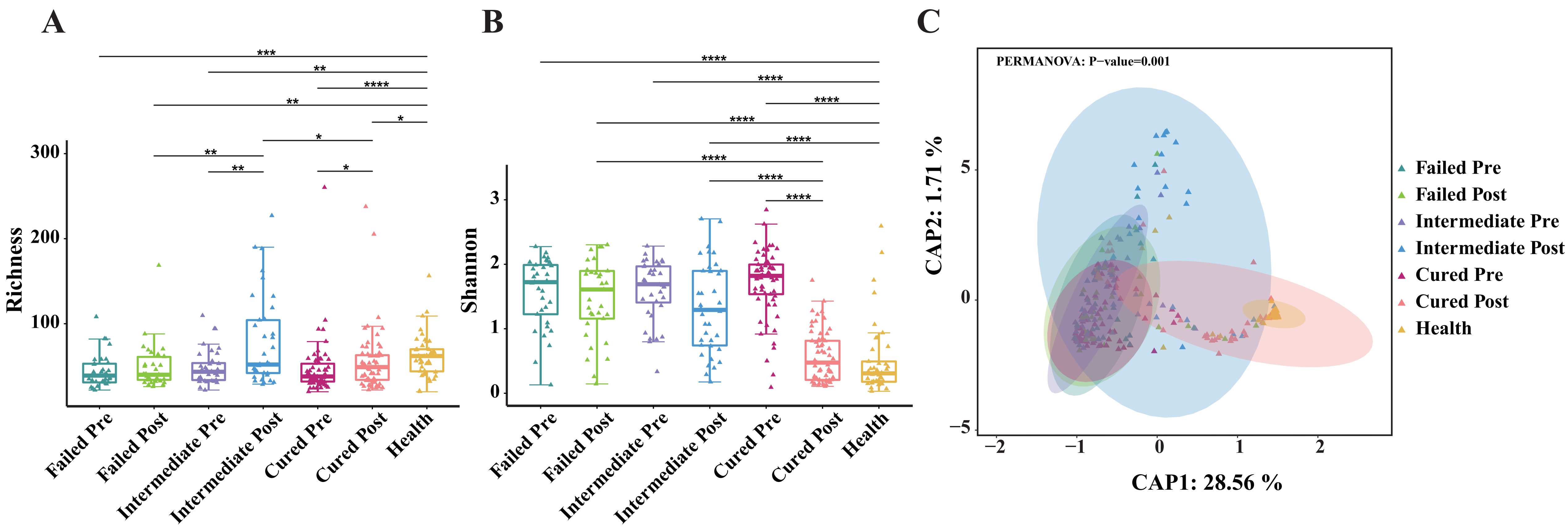

[Supplementary Figure 4] Antimicrobial activity test of *L. johnsonii* or *L. iners* against *G. vaginalis* or *F. vaginae*

A. Shows broths we used for antimicrobial activity tests. The clarity, brightness and contrast ratio were adjusted to better show the inhibition zone. The black dashed line marked the estimated edge of inhibition zone in each broth.

B. Shows the radium of inhibition zone in each broth.

A

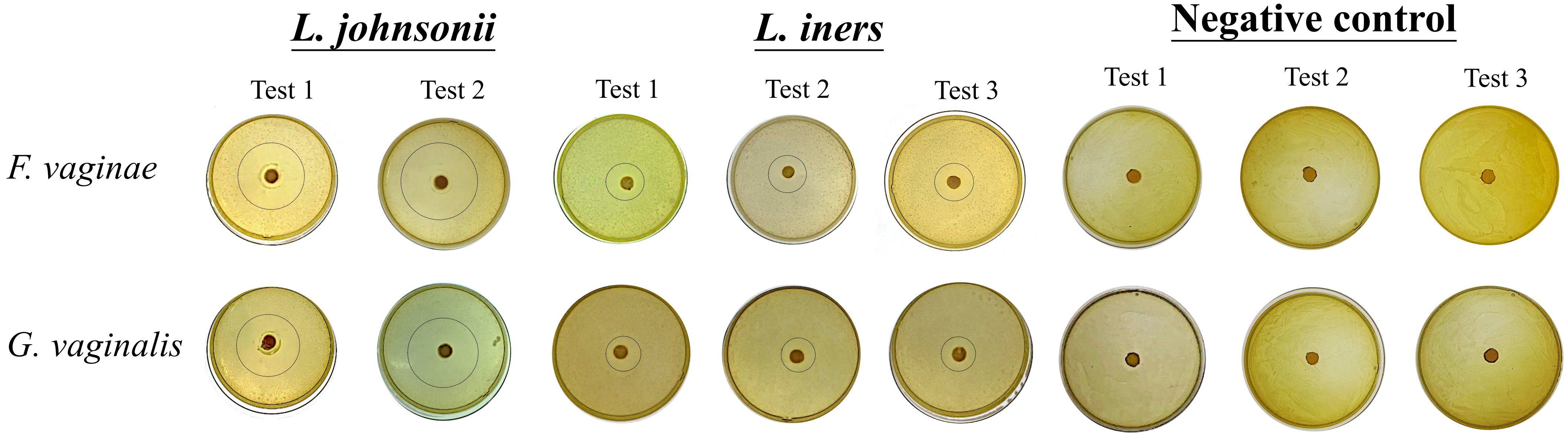

B

	L. johnsonii [a]		*L. iners* [a]			Negative Control [a]		
	Test 1	Test 2	Test 1	Test 2	Test 3	Test 1	Test 2	Test 3
F. vaginae	4.6cm	5.5cm	2.5cm	2.7cm	3.5cm	0cm	0cm	0cm
G. vaginalis	4.2cm	4.5cm	1.9cm	2.0cm	2.0cm	0cm	0cm	0cm

[a] This table shows the radium of inhibition zone in each broth.

[Supplementary Table 1] 16S rDNA sequence of *Gardnerella* genomospecies and *Lactobacillus* spp involved in this study

Species	Strain	Sequence
Lactobacillus Crispatus	TMPC 9023F	TAAGCATAGACGGCTATACATGCAAGTCGAGCGAGCGGAACTAACAGATTTACTTCGGTAATGACGTTAGGAAAGCG<br>AGCGGCGGATGGGTGAGTAACACGTGGGGAACCTGCCCCATAGTCTGGGATACCACTTGGAAAAGGTGCTAATACCG<br>GATAAGAAAGCAGATCGCATGATCAGCTTTTAAAAGGCGGCGTAAGCTGTCGCTATGGGATGGCCCCGCGGTGCATT<br>AGCTAGTTGGTAAGGTAAAGGCTTACCAAGGCGATGATGCATAGCCGAGTTGAGAGACTGATCGGCCACATTGGGAC<br>TGAGACACGGCCCAAACTCCTACGGGAGGCAGCAGTAGGGAATCTTCCACAATGGACGCAAGTCTGATGGAGCAACG<br>CCGCGTGAGTGAAGAAGGTTTTCGGATCGTAAAGCTCTGTTGTTGGTGAAGAAGGATAGAGGTAGTAACTGGCCTTTA<br>TTTGACGGTAATCAACCAGAAAGTCACGGCTAACTACGTGCCAGCAGCCGCGGTAATACGTAGGTGGCAAGCGTTGTC<br>CGGATTTATTGGGCGTAAAGCGAGCGCAGGCGGAAGAATAAGTCTGATGTGAAAGCCCTCGGCTTAACCGAGGAACT<br>GCATCGGAAACTGTTTTTCTTGAGTGCAGAAGAGGAGAGTGGAACTCCATGTGTAGCGGTGGAATGCGTAGATATATG<br>GAAGAACACCAGTGGCGAAGGCGGCTCTCTGGTCTGCAACTGACGCTGAGGCTCGAAAGCATGGGTAGCGAACAGGA<br>TTAGATACCCTGGTAGTCCATGCCGTAAACGATGAGTGCTAAGTGTTGGGAGGTTTCCGCCTCTCAGTGCTGCAGCTA<br>ACGCATTAAGCACTCCGCCTGGGGAGTACGACCGCAAGGTTGAAACTCAAAGGAATTGACGGGGGCCCGCACAAGCG<br>GTGGAGCATGTGGTTTAATTCGAAGCAACGCGAAGAACCTTACCAGGTCTTGACATCTAGTGCCATTTGTAGAGATAC<br>AAAGTTCCCTTCGGGGACGCTAAGACAGGTGGTGCATGGCTGTCGTCAGCTCGTGTCGTGAGATGTTGGGTTAAGTCC<br>CGCAACGAGCGCAACCCTTGTTATTAGTTGCCAGCATTAAGTTGGGCACTCTAATGAGACTGCCGGTGACAAACCGGA<br>GGAAGGTGGGGATGACGTCAAGTCATCATGCCCCTTATGACCTGGGCTACACACGTGCTACAATGGGCAGTACAACG<br>AGAAGCGAGCCTGCGAAGGCAAGCGAATCTCTGAAAGCTGTTCTCAGTTCGGACTGCAGTCTGCAACTCGACTGCACG<br>AAGCTGGAATCGCTAGTAATCGCGGATCAGCACGCCGCGGTGAATACGTTCCCGGGCCTTGTACACACCGCCCGTCAC<br>ACCATGGGAGTCTGCAATGCCCAAAGCCGGTGGCCTAACCTTCGGGAAGGAGCCGTCTAAGGCAGGGCAGATGACTG<br>GGGTGAAGTCGTAACAAGAGCATTCAC
	15267	TCGAGCGAGCGGAACTAACAGATTTACTTCGGTAATGACGTTAGGAAAGCGAGCGGCGGATGGGTGAGTAACACGTG<br>GGGAACCTGCCCCATAGTCTGGGATACCACTTGGAAACAGGTGCTAATACCGGATAAGAAAGCAGATCGCATGATCA<br>GCTTTTAAAAGGCGGCGTAAGCTGTCGCTATGGGATGGCCCCGCGGTGCATTAGCTAGTTGGTAAGGTAAAGGCTTAC<br>CAAGGCGATGATGCATAGCCGAGTTGAGAGACTGATCGGCCACATTGGGACTGAGACACGGCCCAAACTCCTACGGG<br>AGGCAGCAGTAGGGAATCTTCCACAATGGACGCAAGTCTGATGGAGCAACGCCGCGTGAGTGAAGAAGGTTTTCGGA<br>TCGTAAAGCTCTGTTGTTGGTGAAGAAGGATAGAGGTAGTAACTGGCCTTTATTTGACGGTAATCAACCAGAAAGTCA<br>CGGCTAACTACGTGCCAGCAGCCGCGGTAATACGTAGGTGGCAAGCGTTGTCCGGATTTATTGGGCGTAAAGCGAGC<br>GCAGGCGGAAGAATAAGTCTGATGTGAAAGCCCTCGGCTTAACCGAGGAACTGCATCGGAAACTGTTTTTCTTGAGTG<br>CAGAAGAGGAGAGTGGAACTCCATGTGTAGCGGTGGAATGCGTAGATATATGGAAGAACACCAGTGGCGAAGGCGG<br>CTCTCTGGTCTGCAACTGACGCTGAGGCTCGAAAGCATGGGTAGCGAACAGGATTAGATACCCTGGTAGTCCATGCCG<br>TAAACGATGAGTGCTAAGTGTTGGGAGGTTTCCGCCTCTCAGTGCTGCAGCTAACGCATTAAGCACTCCGCCTGGGGA<br>GTACGACCGCAAGGTTGAAACTCAAAGGAATTGACGGGGGCCCGCACAAGCGGTGGAGCATGTGGTTTAATTCGAAG

		CAACGCGAAGAACCTTACCAGGTCTTGACATCTAGTGCCATTTGTAGAGATACAAAGTTCCCTTCGGGGACGCTAAGA<br>CAGGTGGTGCATGGCTGTCGTCAGCTCGTGTCGTGAGATGTTGGGTTAAGTCCCGCAACGAGCGCAACCCTTGTTATT<br>AGTTGCCAGCATTAAGTTGGGCACTCTAATGAGACTGCCGGTGACAAACCGGAGGAAGGTGGGGATGACGTCAAGTC<br>ATCATGCCCCTTATGACCTGGGCTACACACGTGCTACAATGGGCAGTACAACGAGAAGCGAGCCTGCGAAGGCAAGC<br>GAATCTCTGAAAGCTGTTCTCAGTTCGGACTGCAGTCTGCAACTCGACTGCACGAAGCTGGAATCGCTAGTAATCGCG<br>GATCAGCACGCCGCGGTGAATACGTTCCCGGGCCTTGTACACACCGCCCGTCACACCATGGGAGTCTGCAATGCCCAA<br>AGCCGGTGGCCTAACCTTCGGGAAGGAGCC
	20278	AAGTCGAGCGAGCGGAACTAACAGATTTACTTCGGTAATGACGTTAGGAAAGCGAGCGGCGGATGGGTGAGTAACAC<br>GTGGGGAACCTGCCCCATAGTCTGGGATACCACTTGGAAACAGGTGCTAATACCGGATAAGAAAGCAGATCGCATGA<br>TCAGCTTTTAAAAGGCGGCGTAAGCTGTCGCTATGGGATGGCCCCGCGGTGCATTAGCTAGTTGGTAAGGTAAAGGCT<br>TACCAAGGCGATGATGCATAGCCGAGTTGAGAGACTGATCGGCCACATTGGGACTGAGACACGGCCCAAACTCCTAC<br>GGGAGGCAGCAGTAGGGAATCTTCCACAATGGACGCAAGTCTGATGGAGCAACGCCGCGTGAGTGAAGAAGGTTTTC<br>GGATCGTAAAGCTCTGTTGTTGGTGAAGAAGGATAGAGGTAGTAACTGGCCTTTATTTGACGGTAATCAACCAGAAAG<br>TCACGGCTAACTACGTGCCAGCAGCCGCGGTAATACGTAGGTGGCAAGCGTTGTCCGGATTTATTGGGCGTAAAGCGA<br>GCGCAGGCGGAAGAATAAGTCTGATGTGAAAGCCCTCGGCTTAACCGAGGAACTGCATCGGAAACTGTTTTCTTGAG<br>TGCAGAAGAGGAGAGTGGAACTCCATGTGTAGCGGTGGAATGCGTAGATATATGGAAGAACACCAGTGGCGAAGGC<br>GGCTCTCTGGTCTGCAACTGACGCTGAGGCTCGAAAGCATGGGTAGCGAACAGGATTAGATACCCTGGTAGTCCATGC<br>CGTAAACGATGAGTGCTAAGTGTTGGGAGGTTTCCGCCTCTCAGTGCTGCAGCTAACGCATTAAGCACTCCGCCTGGG<br>GAGTACGACCGCAAGGTTGAAACTCAAAGGAATTGACGGGGGCCCGCACAAGCGGTGGAGCATGTGGTTTAATTCGA<br>AGCAACGCGAAGAACCTTACCAGGTCTTGACATCTAGTGCCATTTGTAGAGATACAAAGTTCCCTTCGGGGACGCTAA<br>GACAGGTGGTGCATGGCTGTCGTCAGCTCGTGTCGTGAGATGTTGGGTTAAGTCCCGCAACGAGCGCAACCCTTGTTA<br>TTAGTTGCCAGCATTAAGTTGGGCACTCTAATGAGACTGCCGGTGACAAACCGGAGGAAGGTGGGGATGACGTCAAG<br>TCATCATGCCCCTTATGACCTGGGCTACACACGTGCTACAATGGGCAGTACAACGAGAAGCGAGCCTGCGAAGGCAA<br>GCGAATCTCTGAAAGCTGTTCTCAGTTCGGACTGCAGTCTGCAACTCGACTGCACGAAGCTGGAATCGCTAGTAATCG<br>CGGATCAGCACGCCGCGGTGAATACGTTCCCGGGCCTTGTACACACCGCCCGTCACACCATGGGAGTCTGCAATGCCC<br>AAAGCCGGTGGCCTAACCTTAGGGAA
	ATCC 33820	GACGAACGCTGGCGGCGTGCCTAATACATGCAAGTCGAGCGAGCGGAACTAACAGATTTACTTCGGTAATGACGTTA<br>GGAAAGCGAGCGGCGGATGGGTGAGTAACACGTGGGGAACCTGCCCCATAGTCTGGGATACCACTTGGAAACAGGTG<br>CTAATACCGGATAAGAAAGCAGATCGCATGATCAGCTTTTAAAAGGCGGCGTAAGCTGTCGCTATGGGATGGCCCCG<br>CGGTGCATTAGCTAGTTGGTAAGGTAAAGGCTTACCAAGGCGATGATGCATAGCCGAGTTGAGAGACTGATCGGCCA<br>CATTGGGACTGAGACACGGCCCAAACTCCTACGGGAGGCAGCAGTAGGGAATCTTCCACAATGGACGCAAGTCTGAT<br>GGAGCAACGCCGCGTGAGTGAAGAAGGTTTTCGGATCGTAAAGCTCTGTTGTTGGTGAAGAAGGATAGAGGTAGTAA<br>CTGGCCTTTATTTGACGGTAATCAACCAGAAAGTCACGGCTAACTACGTGCCAGCAGCCGCGGTAATACGTAGGTGGC<br>AAGCGTTGTCCGGATTTATTGGGCGTAAAGCGAGCGCAGGCGGAAGAATAAGTCTGATGTGAAAGCCCTCGGCTTAA

		CCGAGGAACTGCATCGGAAACTGTTTTTCTTGAGTGCAGAAGAGGAGAGTGGAACTCCATGTGTAGCGGTGGAATGC<br>GTAGATATATGGAAGAACACCAGTGGCGAAGGCGGCTCTCTGGTCTGCAACTGACGCTGAGGCTCGAAAGCATGGGT<br>AGCGAACAGGATTAGATACCCTGGTAGTCCATGCCGTAAACGATGAGTGCTAAGTGTTGGGAGGTTTCCGCCTCTCAG<br>TGCTGCAGCTAACGCATTAAGCACTCCGCCTGGGGAGTACGACCGCAAGGTTGAAACTCAAAGGAATTGACGGGGGC<br>CCGCACAAGCGGTGGAGCATGTGGTTTAATTCGAAGCAACGCGAAGAACCTTACCAGGTCTTGACATCTAGTGCCATT<br>TGTAGAGATACAAAGTTCCCTTCGGGGACGCTAAGACAGGTGGTGCATGGCTGTCGTCAGCTCGTGTCGTGAGATGTT<br>GGGTTAAGTCCCGCAACGAGCGCAACCCTTGTTATTAGTTGCCAGCATTAAGTTGGGCACTCTAATGAGACTGCCGGT<br>GACAAACCGGAGGAAGGTGGGGATGACGTCAAGTCATCATGCCCCTTATGACCTGGGCTACACACGTGCTACAATGG<br>GCAGTACAACGAGAAGCGAGCCTGCGAAGGCAAGCGAATCTCTGAAAGCTGTTCTCAGTTCGGACTGCAGTCTGCAA<br>CTCGACTGCACGAAGCTGGAATCGCTAGTAATCGCGGATCAGCACGCCGCGGTGAATACGTTCCCGGGCCTTGTACAC<br>ACCGCCCGTCACACCATGGGAGTCTGCAATGCCCAAAGCCGGTGGCCTAACCTTCGGGAAGGAGCCGTCTAAGGCAG<br>GGCAGATGACTGGGGTGAAGTCGTAACAAGGTAGCCGTAGGAGAACTGC
	BJ H42-4	TCTGCACTCAAGAAAAACAGTTTCCGATGCAGTTCCTCGGTTAAGCCGAGGGCTTTCACATCAGACTTATTCTTCCGCC<br>TGCGCTCGCTTTACGCCCAATAAATCCGGACAACGCTTGCCACCTACGTATTACCGCGGCTGCTGGCACGTAGTTAGC<br>CGTGACTTTCTGGTTGATTACCGTCAAATAAAGGCCAGTTACTACCTCTATCCTTCTTCACCAACAACAGAGCTTTACG<br>ATCCGAAAACCTTCTTCACTCACGCGGCGTTGCTCCATCAGACTTGCGTCCATTGTGGAAGATTCCCTACTGCTGCCTC<br>CCGTAGGAGTTTGGGCCGTGTCTCAGTCCCAATGTGGCCGATCAGTCTCTCAACTCGGCTATGCATCATCGCCTTGGTA<br>AGCCTTTACCTTACCAACTAGCTAATGCACCGCGGGGCCATCCCATAGCGACAGCTTACGCCGCCTTTTAAAAGCTGA<br>TCATGCGATCTGCTTTCTTATCCGGTATTAGCACCTGTTTCCAAGTGGTATCCCAGACTATGGGGCAGGTTCCCCACGT<br>GTTACTCACCCATCCGCCGCTCGCTTTCCTAACGTCATTACCGAAGTAAATCTGTTAGTTCCGCTCGCTCGACTTGCAT<br>GTATTAGGCACGCCGCCAGCGTTCGTCTGAGCCAT
	TMPC 9023F	TAAGCATAGACGGCTATACATGCAAGTCGAGCGAGCGGAACTAACAGATTTACTTCGGTAATGACGTTAGGAAAGCG<br>AGCGGCGGATGGGTGAGTAACACGTGGGGAACCTGCCCCATAGTCTGGGATACCACTTGGAAACAGGTGCTAATACC<br>GGATAAGAAAGCAGATCGCATGATCAGCTTTTAAAAGGCGGCGTAAGCTGTCGCTATGGGATGGCCCCGCGGTGCAT<br>TAGCTAGTTGGTAAGGTAAAGGCTTACCAAGGCGATGATGCATAGCCGAGTTGAGAGACTGATCGGCCACATTGGGA<br>CTGAGACACGGCCCAAACTCCTACGGGAGGCAGCAGTAGGGAATCTTCCACAATGGACGCAAGTCTGATGGAGCAAC<br>GCCGCGTGAGTGAAGAAGGTTTTCGGATCGTAAAGCTCTGTTGTTGGTGAAGAAGGATAGAGGTAGTAACTGGCCTTT<br>ATTTGACGGTAATCAACCAGAAAGTCACGGCTAACTACGTGCCAGCAGCCGCGGTAATACGTAGGTGGCAAGCGTTG<br>TCCGGATTTATTGGGCGTAAAGCGAGCGCAGGCGGAAGAATAAGTCTGATGTGAAAGCCCTCGGCTTAACCGAGGAA<br>CTGCATCGGAAACTGTTTTTCTTGAGTGCAGAAGAGGAGAGTGGAACTCCATGTGTAGCGGTGGAATGCGTAGATATA<br>TGGAAGAACACCAGTGGCGAAGGCGGCTCTCTGGTCTGCAACTGACGCTGAGGCTCGAAAGCATGGGTAGCGAACAG<br>GATTAGATACCCTGGTAGTCCATGCCGTAAACGATGAGTGCTAAGTGTTGGGAGGTTTCCGCCTCTCAGTGCTGCAGC<br>TAACGCATTAAGCACTCCGCCTGGGGAGTACGACCGCAAGGTTGAAACTCAAAGGAATTGACGGGGGCCCGCACAAG<br>CGGTGGAGCATGTGGTTTAATTCGAAGCAACGCGAAGAACCTTACCAGGTCTTGACATCTAGTGCCATTTGTAGAGAT

		ACAAAGTTCCCTTCGGGGACGCTAAGACAGGTGGTGCATGGCTGTCGTCAGCTCGTGTCGTGAGATGTTGGGTTAAGT<br>CCCGCAACGAGCGCAACCCTTGTTATTAGTTGCCAGCATTAAGTTGGGCACTCTAATGAGACTGCCGGTGACAAACCG<br>GAGGAAGGTGGGGATGACGTCAAGTCATCATGCCCCTTATGACCTGGGCTACACACGTGCTACAATGGGCAGTACAA<br>CGAGAAGCGAGCCTGCGAAGGCAAGCGAATCTCTGAAAGCTGTTCTCAGTTCGGACTGCAGTCTGCAACTCGACTGCA<br>CGAAGCTGGAATCGCTAGTAATCGCGGATCAGCACGCCGCGGTGAATACGTTCCCGGGCCTTGTACACACCGCCCGTC<br>ACACCATGGGAGTCTGCAATGCCCAAAGCCGGTGGCCTAACCTTCGGGAAGGAGCCGTCTAAGGCAGGGCAGATGAC<br>TGGGGTGAAGTCGTAACAAGAGCATTCAC
Lactobacillus gasseri	HDB1102	CGAACGCTGGCGGCGTGCCTAATACATGCAAGTCGAGCGAGCTTGCCTAGATGAATTTGGTGCTTGCACCAAATGAAA<br>CTAGATACAAGCGAGCGGCGGACGGGTGAGTAACACGTGGGTAACCTGCCCAAGAGACTGGGATAACACCTGGAAAC<br>AGATGCTAATACCGGATAACAACACTAGACGCATGTCTAGAGTTTAAAAGATGGTTCTGCTATCACTCTTGGATGGAC<br>CTGCGGTGCATTAGCTAGTTGGTAAGGTAACGGCTTACCAAGGCAATGATGCATAGCCGAGTTGAGAGACTGATCGGC<br>CACATTGGGACTGAGACACGGCCCAAACTCCTACGGGAGGCAGCAGTAGGGAATCTTCCACAATGGACGCAAGTCTG<br>ATGGAGCAACGCCGCGTGAGTGAAGAAGGGTTTCGGCTCGTAAAGCTCTGTTGGTAGTGAAGAAAGATAGAGGTAGT<br>AACTGGCCTTTATTTGACGGTAATTACTTAGAAAGTCACGGCTAACTACGTGCCAGCAGCCGCGGTAATACGTAGGTG<br>GCAAGCGTTGTCCGGATTTATTGGGCGTAAAGCGAGTGCAGGCGGTTCAATAAGTCTGATGTGAAAGCCTTCGGCTCA<br>ACCGGAGAATTGCATCAGAAACTGTTGAACTTGAGTGCAGAAGAGGAGAGTGGAACTCCATGTGTAGCGGTGGAATG<br>CGTAGATATATGGAAGAACACCAGTGGCGAAGGCGGCTCTCTGGTCTGCAACTGACGCTGAGGCTCGAAAGCATGGG<br>TAGCGAACAGGATTAGATACCCTGGTAGTCCATGCCGTAAACGATGAGTGCTAAGTGTTGGGAGGTTTCCGCCTCTCA<br>GTGCTGCAGCTAACGCATTAAGCACTCCGCCTGGGGAGTACGACCGCAAGGTTGAAACTCAAAGGAATTGACGGGGG<br>CCCGCACAAGCGGTGGAGCATGTGGTTTAATTCGAAGCAACGCGAAGAACCTTACCAGGTCTTGACATCCAGTGCAA<br>ACCTAAGAGATTAGGAGTTCCCTTCGGGGACGCTGAGACAGGTGGTGCATGGCTGTCGTCAGCTCGTGTCGTGAGATG<br>TTGGGTTAAGTCCCGCAACGAGCGCAACCCTTGTCATTAGTTGCCATCATTAAGTTGGGCACTCTAATGAGACTGCCG<br>GTGACAAACCGGAGGAAGGTGGGGATGACGTCAAGTCATCATGCCCCTTATGACCTGGGCTACACACGTGCTACAAT<br>GGACGGTACAACGAGAAGCGAACCTGCGAAGGCAAGCGGATCTCTGAAAGCCGTTCTCAGTTCGGACTGTAGGCTGC<br>AACTCGCCTACACGAAGCTGGAATCGCTAGTAATCGCGGATCAGCACGCCGCGGTGAATACGTTCCCGGGCCTTGTAC<br>ACACCGCCCGTCACACCATGAGAGTCTGTAACACCCAAAGCCGGTGGGATAACCTTTATAGGAGTCAGCCGTCTAAGG<br>TAGGACAGATGATTAGGGTGAATCTAAAAG
	ATCC 33323	GAAAATGAGAGTTTGATCCTGGCTCAGGACGAACGCTGGCGGCGTGCCTAATACATGCAAGTCGAGCGAGCTTGCCT<br>AGATGAATTTGGTGCTTGCACCAGATGAAACTAGATACAAGCGAGCGGCGGACGGGTGAGTAACACGTGGGTAACCT<br>GCCCAAGAGACTGGGATAACACCTGGAAACAGATGCTAATACCGGATAACAACACTAGACGCATGTCTAGAGTTTAA<br>AAGATGGTTCTGCTATCACTCTTGGATGGACCTGCGGTGCATTAGCTAGTTGGTAAGGTAACGGCTTACCAAGGCAAT<br>GATGCATAGCCGAGTTGAGAGACTGATCGGCCACATTGGGACTGAGACACGGCCCAAACTCCTACGGGAGGCAGCAG<br>TAGGGAATCTTCCACAATGGACGCAAGTCTGATGGAGCAACGCCGCGTGAGTGAAGAAGGGTTTCGGCTCGTAAAGC<br>TCTGTTGGTAGTGAAGAAAGATAGAGGTAGTAACTGGCCTTTATTTGACGGTAATTACTTAGAAAGTCACGGCTAACT

		ACGTGCCAGCAGCCGCGGTAATACGTAGGTGGCAAGCGTTGTCCGGATTTATTGGGCGTAAAGCGAGTGCAGGCGGTTCAATAAGTCTGATGTGAAAGCCTTCGGCTCAACCGGAGAATTGCATCAGAAACTGTTGAACTTGAGTGCAGAAGAGGAGAGTGGAACTCCATGTGTAGCGGTGGAATGCGTAGATATATGGAAGAACACCAGTGGCGAAGGCGGCTCTCTGGTCTGCAACTGACGCTGAGGCTCGAAAGCATGGGTAGCGAACAGGATTAGATACCCTGGTAGTCCATGCCGTAAACGATGAGTGCTAAGTGTTGGGAGGTTTCCGCCTCTCAGTGCTGCAGCTAACGCATTAAGCACTCCGCCTGGGGAGTACGACCGCAAGGTTGAAACTCAAAGGAATTGACGGGGGCCCGCACAAGCGGTGGAGCATGTGGTTTAATTCGAAGCAACGCGAAGAACCTTACCAGGTCTTGACATCCAGTGCAAACCTAAGAGATTAGGTGTTCCCTTCGGGGACGCTGAGACAGGTGGTGCATGGCTGTCGTCAGCTCGTGTCGTGAGATGTTGGGTTAAGTCCCGCAACGAGCGCAACCCTTGTCATTAGTTGCCATCATTAAGTTGGGCACTCTAATGAGACTGCCGGTGACAAACCGGAGGAAGGTGGGGATGACGTCAAGTCATCATGCCCCTTATGACCTGGGCTACACACGTGCTACAATGGACGGTACAACGAGAAGCGAACCTGCGAAGGCAAGCGGATCTCTGAAAGCCGTTCTCAGTTCGGACTGTAGGCTGCAACTCGCCTACACGAAGCTGGAATCGCTAGTAATCGCGGATCAGCACGCCGCGGTGAATACGTTCCCGGGCCTTGTACACACCGCCCGTCACACCATGAGAGTCTGTAACACCCAAAGCCGGTGGGATAACCTTTATAGGAGTCAGCCGTCTAAGGTAGGACAGATGATTAGGGTGAAGTCGTAACAAGGTAGCCGTAGGAGAACCTGCGGCTGGATCACCTCCTTT
	896-2	AAACTGCAGTCGAGCGAGCTTGCCTAGATGAATTTGGTGCTTGCACCAGATGAAACTAGATACAAGCGAGCGGCGGACGGGTGAGTAACACGTGGGTAACCTGCCCAAGAGACTGGGATAACACCTGGAAACAGATGCTAATACCGGATAACAACACTAGACGCATGTCTAGAGTTTAAAAGATGGTTCTGCTATCACTCTTGGATGGACCTGCGGTGCATTAGCTAGTTGGTAAGGTAACGGCTTACCAAGGCAATGATGCATAGCCGAGTTGAGAGACTGATCGGCCACATTGGGACTGAGACACGGCCCAAACTCCTACGGGAGGCAGCAGTAGGGAATCTTCCACAATGGACGCAAGTCTGATGGAGCAACGCCGCGTGAGTGAAGAAGGGTTTCGGCTCGTAAAGCTCTGTTGGTAGTGAAGAAAGATAGAGGTAGTAACTGGCCTTTATTTGACGGTAATTACTTAGAAAGTCACGGCTAACTACGTGCCAGCAGCCGCGGTAATACGTAGGTGGCAAGCGTTGTCCGGATTTATTGGGCGTAAAGCGAGTGCAGGCGGTTCAATAAGTCTGATGTGAAAGCCTTCGGCTCAACCGGAGAATTGCATCAGAAACTGTTGAACTTGAGTGCAGAAGAGGAGAGTGGAACTCCATGTGTAGCGGTGGAATGCGTAGATATATGGAAGAACACCAGTGGCGAAGGCGGCTCTCTGGTCTGCAACTGACGCTGAGGCTCGAAAGCATGGGTAGCGAACAGGATTAGATACCCTGGTAGTCCATGCCGTAAACGATGAGTGCTAAGTGGTGGGAGGTTTCCGCCTCTCAGTGCTGCAGCTAACGCATTAAGCACTCCGCCTGGGGAGTACGACCGCAAGGTTGAAACTCAAAGGAATTGACGGGGGCCCGCACAAGCGGTGGAGCATGTGGTTTAATTCGAAGCAACGCGAAGAACCTTACCAGGTCTTGACATCCAGTGCAAACCTAAGAGATTAGGTGTTCCCTTCGGGGACGCTGAGACAGGTGGTGCATGGCTGTCGTCAGCTCGTGTCGTGAGATGTTGGGTTAAGTCCCGCAACGAGCGCAACCCTTGTCATTAGTTGCCATCATTAAGTTGGGCACTCTAATGAGACTGCCGGTGACAAACCGGAGGAAGGTGGGGATGACGTCAAGTCATCATGCCCCTTATGACCTGGGCTACACACGTGCTACAATGGACGGTACAACGAGAAGCGAACCTGCGAAGGCAAGCGGATCTCTGAAAGCCGTTCTCAGTTCGGACTGTAGGCTGCAACTCGCCTACACGAAGCTGGAATCGCTAGTAATCGCCGA
	545	CGCTGGCGGCGTGCCTAATACATGCAAGTCGAGCGAGCTTGCCTAGATGAATTTGGTGCTTGCACCAAATGAAACTAGATACAAGCGAGCGGCGGACGGGTGAGTAACACGTGGGTAACCTGCCCAAGAGACTGGGATAACACCTGGAAACAGA

		TGCTAATACCGGATAACAACACTAGACGCATGTCTAGAGTTTAAAAGATGGTTCTGCTATCACTCTTGGATGGACCTG<br>CGGTGCATTAGCTAGTTGGTAAGGTAACGGCTTACCAAGGCAATGATGCATAGCCGAGTTGAGAGACTGATCGGCCA<br>CATTGGGACTGAGACACGGCCCAAACTCCTACGGGAGGCAGCAGTAGGGAATCTTCCACAATGGACGCAAGTCTGAT<br>GGAGCAACGCCGCGTGAGTGAAGAAGGGTTTCGGCTCGTAAAGCTCTGTTGGTAGTGAAGAAAGATAGAGGTAGTAA<br>CTGGCCTTTATTTGACGGTAATTACTTAGAAAGT
	345A	AGAGTTTGATCCTGGCTCAGGACGAACGCTGGCGGCGTGCCTAATACATGCAAGTCGAGCGAGCTTGCCTAGATGAAT<br>TTGGTGCTTGCACCAAATGAAACTAGATACAAGCGAGCGGCGGACGGGTGAGTAACACGTGGGTAACCTGCCCAAGA<br>GACTGGGATAACACCTGGAAACAGATGCTAATACCGGATAACAACACTAGACGCATGTCTAGAGTTTAAAAGATGGT<br>TCTGCTATCACTCTTGGATGGACCTGCGGTGCATTAGCTAGTTGGTAAGGTAACGGCTTACCAAGGCAATGATGCATA<br>GCCGAGTTGAGAGACTGATCGGCCACATTGGGACTGAGACACGGCCCAAACTCCTACGGGAGGCAGCAGTAGGGAAT<br>CTTCCACAATGGACGCAAGTCTGATGGAGCAACGCCGCGTGAGTGAAGAAGGGTTTCGGCTCGTAAAGCTCTGTTGGT<br>AGTGAAGAAAGATAGAGGTAGTAACTGGCCTTTATTTGACGGTAATTACTTAGAAAGTCACGGCTAACTACGTGCCAG<br>CAGCCGCGGTAATACGTAGGTGGCAAGCGTTGTCCGGATTTATTGGGCGTAAAGCGAGTGCAGGCGGTTCAATAAGTC<br>TGATGTGAAAGCCTTCGGCTCAACCGGAGAATTGCATCAGAAACTGTTGAACTTGAGTGCAGAAGAGGAGAGTGGAA<br>CTCCATGTGTAGCGGTGGAATGCGTAGATATATGGAAGAACACCAGTGGCGAAGGCGGCTCTCTGGTCTGCAACTGAC<br>GCTGAGGCTCGAAAGCATGGGTAGCGAACAGGATTAGATACCCTGGTAGTCCATGCCGTAAACGATGAGTGCTAAGT<br>GTTGGGAGGTTTCCGCCTCTCAGTGCTGCAGCTAACGCATTAAGCACTCCGCCTGGGGAGTACGACCGCAAGGTTGAA<br>ACTCAAAGGAATTGACGGGGGCCCGCACAAGCGGTGGAGCATGTGGTTTAATTCGAAGCAACGCGAAGAACCTTACC<br>AGGTCTTGACATCCAGTGCAAACCTAAGAGATTAGGTGTTCCCTTCGGGGACGCTGAGACAGGTGGTGCATGGCTGTC<br>GTCAGCTCGTGTCGTGAGATGTTGGGTTAAGTCCCGCAACGAGCGCAACCCTTGTCATTAGTTGCCATCATTAAGTTGG<br>GCACTCTAATGAGACTGCCGGTGACAAACCGGAGGAAGGTGGGGATGACGTCAAGTCATCATGCCCCTTATGACCTG<br>GGCTACACACGTGCTACAATGGACGGTACAACGAGAAGCGAACCTGCGAAGGTAAGCGGATCTCTGAAAGCCGTTCT<br>CAGTTCGGACTGTAGGCTGCAACTCGCCTACACGAAGCTGGAATCGCTAGTAATCGCGGATCAGCACGCCGCGGTGA<br>ATACGTTCCCGGGCCTTGTACACACCGCCCGTCACACCATGAGAGTCTGTAACACCCAAAGCCGGTGGGATAACCTTT<br>ATAGGAGTCAGCCGTCTAAGGTAGGACAGATGATTAGGGTGAAGTCGTAACAAGGTAGCCGTAGGAGAACCTGCGGC<br>TGGATCACCT
Lactobacillus iners	LC483559.1	GGCTCAGGACGAACGCTGGCGGCGTGCCTAATACATGCAAGTCGAGCGAGTCTGCCTTGAAGATCGGAGTGCTTGCA<br>CTCTGTGAAACAAGATACAGGCTAGCGGCGGACGGGTGAGTAACACGTGGGTAACCTGCCCAAGAGATCGGGATAAC<br>ACCTGGAAACAGATGCTAATACCGGATAACAACAGATGATGCCTATCAACTGTTTAAAAGATGGTTCTGCTATCACTC<br>TTGGATGGACCTGCGGTGCATTAGCTAGTTGGTAGGGTAACGGCCTACCAAGGCGATGATGCATAGCCGAGTTGAGA<br>GACTGATCGGCCACATTGGGACTGAGACACGGCCCAAACTCCTACGGGAGGCAGCAGTAGGGAATCTTCCACAATGG<br>ACGCAAGTCTGATGGAGCAACGCCGCGTGAGTGAAGAAGGGTTTCGGCTCGTAAAGCTCTGTTGTTGGTGAAGAAGG<br>ACAGGGGTAGTAACTGACCTTTGTTTGACGGTAATCAATTAGAAAGTCACGGCTAACTACGTGCCAGCAGCCGCGGTA<br>ATACGTAGGTGGCAAGCGTTGTCCGGATTTATTGGGCGTAAAGCGAGTGCAGGCGGCTCGATAAGTCTGATGTGAAA

		GCCTTCGGCTCAACCGGAGAATTGCATCAGAAACTGTCGAGCTTGAGTACAGAAGAGGAGAGTGGAACTCCATGTGT<br>AGCGGTGAAATGCGTAGATATATGGAAGAACACCGGTGGCGAAGGCGGCTCTCTGGTCTGTTACTGACGCTGAGGCT<br>CGAAAGCATGGGTAGCGAACAGGATTAGATACCCTGGTAGTCCATGCCGTAAACGATGAGTGCTAAGTGTTGGGAGG<br>TTTCCGCCTCTCAGTGCTGCAGCTAACGCATTAAGCACTCCGCCTGGGGAGTACGACCGCAAGGTTGAAACTCAAAGG<br>AATTGACGGGGGCCCGCACAAGCGGTGGAGCATGTGGTTTAATTCGAAGCAACGCGAAGAACCTTACCAGGTCTTGA<br>CATCCATAGCCAGTCTAAGAGATTAGATGTTCCCTTCGGGGACTATGAGACAGGTGGTGCATGGCTGTCGTCAGCTCG<br>TGTCGTGAGATGTTGGGTTAAGTCCCGCAACGAGCGCAACCCTTGTCATTAGTTGCCAGCATTAAGTTGGGCACTCTA<br>ATGAGACTGCCGGTGACAAACCGGAGGAAGGTGGGGATGACGTCAAGTCATCATGCCCCTTATGACCTGGGCTACAC<br>ACGTGCTACAATGGACGGTACAACGAGAAGCGACCCTGTGAAGGCAAGCGGATCTCTGAAAGCCGTTCTCAGTTCGG<br>ATTGCAGGCTGCAACTCGCCTGCATGAAGCTGGAATCGCTAGTAATCGCAAATCAGCACGTTGCGGTGAATACGTTCC<br>CGGGCCTTGTACACACCGCCCGTCACACCATGAGAGTCTGTAACGCCCGAAGCCGGCGGGATAACCGAAAGGAGTCA<br>GCCGTCTAAGGCGGGACAGATGATTAGGGTGAAGTCG
	AY526083.1	GACGAACGCTGGCGGCGTGCCTAATACATGCAAGTCGAGCGAGTCTGCCTTGAAGATCGGAGTGCTTGCACTCTGTGA<br>AACAAGATACAGGCTAGCGGCGGACGGGTGAGTAACACGTGGGTAACCTGCCCAAGAGATCGGGATAACACCTGGA<br>AACAGATGCTAATACCGGATAACAACAGATGATGCCTATCAACTGTTTAAAAGATGGTTCTGCTATCACTCTTGGATG<br>GACCTGCGGTGCATTAGCTAGTTGGTAGGGTAACGGCCTACCAAGGCGATGATGCATAGCCGAGTTGAGAGACTGAT<br>CGGCCACATTGGGACTGAGACACGGCCCAAACTCCTACGGGAGGCAGCAGTAGGGAATCTTCCACAATGGACGCAAG<br>TCTGATGGAGCAACGCCGCGTGAGTGAAGAAGGGTTTCGGCTCGTAAAGCTCTGTTGTTGGTGAAGAAGGACAGGGG<br>TAGTAACTGACCTTTGTTTGACGGTAATCAATTAGAAAGTCACGGCTAACTACGTGCCAGCAGCCGCGGTAATACGTA<br>GGTGGCAAGCGTTGTCCGGATTTATTGGGCGTAAAGCGAGTGCAGGCGGCTCGATAAGTCTGATGTGAAAGCCTTCGG<br>CTCAACCGGAGAATTGCATCAGAAACTGTCGAGCTTGAGTACAGAAGAGGAGAGTGGAACTCCATGTGTAGCGGTGA<br>AATGCGTAGATATATGGAAGAACACCGGTGGCGAAGGCGGCTCTCTGGTCTGTTACTGACGCTGAGGCTCGAAAGCA<br>TGGGTAGCGAACAGGATTAGATACCCTGGTAGTCCATGCCGTAAACGATGAGTGCTAAGTGTTGGGAGGTTTCCGCCT<br>CTCAGTGCTGCAGCTAACGCATTAAGCACTCCGCCTGGGGAGTACGACCGCAAGGTTGAAACTCAAAGGAATTGACG<br>GGGGCCCGCACAAGCGGTGGAGCATGTGGTTTAATTCGAAGCAACGCGAAGAACCTTACCAGGTCTTGACATCCATA<br>GCCAGTCTAAGAGATTAGATGTTCCCTTCGGGGACTATGAGACAGGTGGTGCATGGCTGTCGTCAGCTCGTGTCGTGA<br>GATGTTGGGTTAAGTCCCGCAACGAGCGCAACCCTTGTCATTAGTTGCCAGCATTAAGTTGGGCACTCTAATGAGACT<br>GCCGGTGACAAACCGGAGGAAGGTGGGGATGACGTCAAGTCATCATGCCCCTTATGACCTGGGCTACACACGTGCTA<br>CAATGGACGGTACAACGAGAAGCGACCCTGTGAAGGCAAGCGGATCTCTGAAAGCCGTTCTCAGTTCGGATTGCAGG<br>CTGCAACTCGCCTGCATGAAGCTGGAATCGCTAGTAATCGCAAATCAGCACGTTGCGGTGAATACGTTCCCGGGCCTT<br>GTACACACCGCCCGTCACACCATGAGAGTCTGTAACGCCCGAAGCCGGCGGGATAACCGAAAGGAGTCAGCCGTCTA<br>AGGCGGGAC
	DSM 13335	TGGCTCAGGACGAACGCTGGCGGCGTGCCTAATACATGCAAGTCGAGCGAGTCTGCCTTGAAGATCGGAGTGCTTGCA<br>CTCTGTGAAACAAGATACAGGCTAGCGGCGGACGGGTGAGTAACACGTGGGTAACCTGCCCAAGAGATCGGGATAAC

		ACCTGGAAACAGATGCTAATACCGGATAACAACAGATGATGCCTATCAACTGTTTAAAAGATGGTTCTGCTATCACTCTTGGATGGACCTGCGGTGCATTAGCTAGTTGGTAGGGTAACGGCCTACCAACGCGATGATGCATAGCCGAGTTGAGAGACTGATCGGCCACATTGGGACTGAGACACGGCCCAAACTCCTACGGGAGGCAGCAGTAGGGAATCTTCCACAATGGACGCAAGTCTGATGGAGCAACGCCGCGTGAGTGAAGAAGGGTTTCGGCTCGTAAAGCTCTGTTGTTGGTGAAGAAGGACAGGGGTAGTAACTGACCTTTGTTTGACGGTAATCAATTAGAAAGTCACGGCTAACTACGTGCCAGCAGCCGCGGTAATACGTAGGTGGCAAGCGTTGTCCGGATTTATTGGGCGTAAAGCGAGTGCAGGCGGCTCGATAAGTCTGATGTGAAAGCCTTCGGCTCAACCGGAGAATTGCATCAGAAACTGTCGAGCTTGAGTACAGAAGAGGAGAGTGGAACTCCATGTGTAGCGGTGAAATGCGTAGATATATGGAAGAACACCGGTGGCGAAGGCGGCTCTCTGGTCTGTTACTGACGCTGAGGCTCGAAAGCATGGGTAGCGAACAGGATTAGATACCCTGGTAGTCCATGCCGTAAACGATGAGTGCTAAGTGTTGGGAGGTTTCCGCCTCTCAGTGCTGCAGCTAACGCATTAAGCACTCCGCCTGGGGAGTACGACCGCAAGGTTGAAACTCAAAGGAATTGACGGGGGCCCGCACAAGCGGTGGAGCATGTGGTTTAATTCGAAGCAACGCGAAGAACCTTACCAGGTCTTGACATCCATAGCCAGTCTAAGAGATTAGATGTTCCCTTCGGGGACTATGAGACAGGTGGTGCATGGCTGTCGTCAGCTCGTGTCGTGAGATGTTGGGTTAAGTCCCGCAACGAGCGCAACCCTTGTCATTAGTTGCCAGCATTAAGTTGGGCACTCTAATGAGACTGCCGGTGACAAACCGGAGGAAGGTGGGGATGACGTCAAGTCATCATGCCCCTTATGACCTGGGCTACACACGTGCTACAATGGACGGTACAACGAGAAGCGACCCTGTGAAGGCAAGCGGATCTCTGAAAGCCGTTCTCAGTTCGGATTGCAGGCTGCAACTCGCCTGCATGAAGCTGGAATCGCTAGTAATCGCAAATCAGCACGTTGCGGTGAATACGTTCCCGGGCCTTGTACACACCGCCCGTCACACCATGAGAGTCTGTAACGCCCGAAGCCGGCGGGATAACCGAAAGGAGTCAGCCGTCTAAGGCGGGACAGATGATTAGGGTGAAGTCGTAACAAGGTAGCCGTAGGAGAACCTGCGGCTG
	L1	CTATACTGCAGTCGAGCGAGTCTGCCTTGAAGATCGGAGTGCTTGCACTCTGTGAAACAAGATACAGGCTAGCGGCGGACGGGTGAGTAACACGTGGGTAACCTGCCCAAGAGATCGGGATAACACCTGGAAACAGATGCTAATACCGGATAACAACAGATGATGCCTATCAACTGTTTAAAAGATGGTTCTGCTATCACTCTTGGATGGACCTGCGGTGCATTAGCTAGTTGGTAGGGTAACGGCCTACCAAGGCGATGATGCATAGCCGAGTTGAGAGACTGATCGGCCACATTGGGACTGAGACACGGCCCAAACTCCTACGGGAGGCAGCAGTAGGGAATCTTCCACAATGGACGCAAGTCTGATGGAGCAACGCCGCGTGAGTGAAGAAGGGTTTCGGCTCGTAAAGCTCTGTTGTTGGTGAAGAAGGACAGGGGTAGTAACTGACCTTTGTTTGACGGTAATCAATTAGAAAGTCACGGCTAACTACGTGCCAGCAGCCGCGGTAATACGTAGGTGGCAAGCGTTGTCCGGATTTATTGGGCGTAAAGCGAGTGCAGGCGGCTCGATAAGTCTGATGTGAAAGCCTTCGGCTCAACCGGAGAATTGCATCAGAAACTGTCGAGCTTGAGTACAGAAGAGGAGAGTGGAACTCCATGTGTAGCGGTGAAATGCGTAGATATATGGAAGAACACCGGTGGCGAAGGCGGCTCTCTGGTCTGTTACTGACGCTGAGGCTCGAAAGCATGGGTAGCGAACAGGATTAGATACCCTGGTAGTCCATGCCGTAAACGATGAGTGCTAAGTGTTGGGAGGTTTCCGCCTCTCAGTGCTGCAGCTAACGCATTAAGCACTCCGCCTGGGGAGTACGACCGCAAGGTTGAAACTCAAAGGAATTGACGGGGGCCCGCACAAGCGGTGGAGCATGTGGTTTAATTCGAAGCAACGCGAAGAACCTTACCAGGTCTTGACATCCATAGCCAGTCTAAGAGATTAGATGTTCCCTTCGGGGACTATGAGACAGGTGGTGCATGGCTGTCGTCAGCTCGTGTCGTGAGATGTTGGGTTAAGTCCCGCAACGAGCGCAACCCTTGTCATTAGTTGCCAGCATTAAGTTGGGCACTCTAATGAGACTGCCGGTGACAAACCGGAGGAAGGTGGGGATGACGTCAAGTCATCATGCCCCTTATGACCTGGGCTACACACGTGCTACAATGGACGGTACAACGAGAAGCGAC

		CCTGTGAAGGCAAGCGGATCTCTGAAAGCCGTTCTCAGTTCGGATTGCAGGCTGCAACTCGCCTGCATGAAGCTGGAA<br>TCGCTAGTAATCGCAAATCAGCACGTTGCGGTGAATACGTTCCCGGGCCTTGTACACACCGCCCGTCACACCATGAGA<br>GTCTGTAACGCCCGAAGCCGGCGGATAACCGAAAGGAGTCAGCCGTCTAGTC
Lactobacillus jensenii	70(02)	AGAGTTTGATCCTGGCTCAGGACGAACGCTGGCGGCGTGCCTAATACATGCAAGTCGAGCGAGCTTGCCTATAGAAAT<br>TCTTCGGAATGGACATAGATACAAGCTAGCGGCGGATGGGTGAGTAACGCGTGGGTAACCTGCCCTTAAGTCTGGGAT<br>ACCATTTGGAAACAGATGCTAATACCGGATAAAAGCTACTTTCGCATGAAAGAAGTTTAAAAGGCGGCGTAAGCTGT<br>CGCTAAAGGATGGACCTGCGATGCATTAGCTAGTTGGTAAGGTAACGGCTTACCAAGGCGATGATGCATAGCCGAGTT<br>GAGAGACTGATCGGCCACATTGGGACTGAGACACGGCCCAAACTCCTACGGGAGGCAGCAGTAGGGAATCTTCCACA<br>ATGGACGAAAGTCTGATGGAGCAACGCCGCGTGAGTGAAGAAGGTTTTCGGATCGTAAAGCTCTGTTGTTGGTGAAG<br>AAGGATAGAGGTAGTAACTGGCCTTTATTTGACGGTAATCAACCAGAAAGTCACGGCTAACTACGTGCCAGCAGCCG<br>CGGTAATACGTAGGTGGCAAGCGTTGTCCGGATTTATTGGGCGTAAAGCGAGCGCAGGCGGATTGATAAGTCTGATGT<br>GAAAGCCTTCGGCTCAACCGAAGAACTGCATCAGAAACTGTCAATCTTGAGTGCAGAAGAGGAGAGTGGAACTCCAT<br>GTGTAGCGGTGGAATGCGTAGATATATGGAAGAACACCAGTGGCGAAGGCGGCTCTCTGGTCTGTAACTGACGCTGA<br>GGCTCGAAAGCATGGGTAGCGAACAGGATTAGATACCCTGGTAGTCCATGCCGTAAACGATGAGTGCTAAGTGTTGG<br>GAGGTTTCCGCCTCTCAGTGCTGCAGCTAACGCATTAAGCACTCCGCCTGGGGAGTACGACCGCAAGGTTGAAACTCA<br>AAGGAATTGACGGGGGCCCGCACAAGCGGTGGAGCATGTGGTTTAATTCGAAGCAACGCGAAGAACCTTACCAGGTC<br>TTGACATCCTTTGACCACCTAAGAGATTAGGTTTTCCCTTCGGGGACAAAGAGACAGGTGGTGCATGGCTGTCGTCAG<br>CTCGTGTCGTGAGA
	17051	TGCAAGTCGAGCGAGCTTGCCTATTGAAATTCTTCGGAATGGACATAGATACAAGCTAGCGGCGGATGGGTGAGTAA<br>CGCGTGGGTAACCTGCCCTTAAGTCTGGGATACCATTTGGAAACAGATGCTAATACCGGATAAAAGCTACTTTCGCAT<br>GAAAGAAGTTTAAAAGGCGGCGTAAGCTGTCGCTAAAGGATGGACCTGCGATGCATTAGCTAGTTGGTAAGGTAACG<br>GCTTACCAAGGCGATGATGCATAGCCGAGTTGAGAGACTGATCGGCCACATTGGGACTGAGACACGGCCCAAACTCC<br>TACGGGAGGCAGCAGTAGGGAATCTTCCACAATGGACGCAAGTCTGATGGAGCAACGCCGCGTGAGTGAAGAAGGTT<br>TTCGGATCGTAAAGCTCTGTTGTTGGTGAAGAAGGATAGAGGTAGTAACTGGCCTTTATTTGACGGTAATCAACCAGA<br>AAGTCACGGCTAACTACGTGCCAGCAGCCGCGGTAATACGTAGGTGGCAAGCGTTGTCCGGATTTATTGGGCGTAAAG<br>CGAGCGCAGGCGGATTGATAAGTCTGATGTGAAAGCCTTCGGCTCAACCGAAGAACTGCATCAGAAACTGTCAATCTT<br>GAGTGCAGAAGAGGAGAGTGGAACTCCATGTGTAGCGGTGGAATGCGTAGATATATGGAAGAACACCAGTGGCGAA<br>GGCGGCTCTCTGGTCTGTAACTGACGCTGAGGCTCGAAAGCATGGGTAGCGAACAGGATTAGATACCCTGGTAGTCCA<br>TGCCGTAAACGATGAGTGCTAAGTGTTGGGAGGTTTCCGCCTCTCAGTGCTGCAGCTAACGCATTAAGCACTCCGCCT<br>GGGGAGTACGACCGCAAGGTTGAAACTCAAAGGAATTGACGGGGGCCCGCACAAGCGGTGGAGCATGTGGTTTAATT<br>CGAAGCAACGCGAAGAACCTTACCAGGTCTTGACATCCTTTGACCACCTAAGAGATTAGGTTTTCCCTTCGGGGACAA<br>AGAGACAGGTGGTGCATGGCTGTCGTCAGCTCGTGTCGTGAGATGTTGGGTTAAGTCCCGCAACGAGCGCAACCCTTG<br>TTAATAGTTGCCAGCATTAAGTTGGGCACTCTATTGAGACTGCCGGTGACAAACCGGAGGAAGGTGGGGATGACGTC<br>AAGTCATCATGCCCCTTATGACCTGGGCTACACACGTGCTACAATGGGCAGTACAACGAGAAGCGAACCTGTGAAGG

		CAAGCGGATCTCTTAAAGCTGTTCTCAGTTCGGACTGTAGGCTGCAACTCGCCTACACGAAGCTGGAATCGCTAGTAA<br>TCGCGGATCAGCACGCCGCGGTGAATACGTTCCCGGGCCTTGTACACACCGCCCGTCACACCATGAGAGTTTGTAACA<br>CCCAAAGTCGGTGAGGTAACCTTTGGAGCCAGCCGCCTAAG
	ATCC 25258	TGCCTAATACATGCAAGTCGAGCGAGCTTGCCTATAGAAGTTCTTCGGAATGGAAATAGATACAAGCTAGCGGCGGAT<br>GGGTGAGTAACGCGTGGGTAACCTGCCCTTAAGTCTGGGATACCATTTGGAAACAGATGCTAATACCGGATAAAAGCT<br>ACTTTCGCATGAAAGAAGTTTAAAAGGCGGCGTAAGCTGTCGTAAAGGATGGACTTGCGATGCATTAGCTAGTTGGTA<br>AGGTAACGGCTTACCAAGGCTGATGATGCATAGCCGAGTTGAGAGACTGATCGGCCACATTGGGACTGAGACACGGC<br>CCAAACTCCTACGGGAGGCAGCAGTAGGGAATCTTCCACAATGGACGAAAGTCTGATGGAGCAACGCCGCGTGAGTG<br>AAGAAGGTTTTCGGATCGTAAAGCTCTGTTGTTGGTGAAGAAGGATAGAGGTAGTAACTGGCCTTTATTTGACGGTAA<br>TCAACCAGAAAGTCACGGCTAACTACGTGCCAGCAGCCGCGGTAATACGTAGGTGGCAAGCGTTGTCCGGATTTATTG<br>GGCGTAAAGCGAGCGCAGGCGGATTGATAAGTCTGATGTGAAAGCCTTCGGCTCAACCGAAGAACTGCATCAGAAAC<br>TGTCAATCTTGAGTGCAGAAGAGGAGAGTGGAACTCCATGTGTAGCGGTGGAATGCGTAGATATATGGAAGAACACC<br>AGTGGCGAAGGCGGCTCTCTGGTCTGTAACTGACGCTGAGGCTCGAAAGCATGGGTAGCGAACAGGATTAGATACCC<br>TGGTAGTCCATGCCGTAAACGATGAGTGCTAAGTGTTGGGAGGTTTCCGCCTCTCAGTGCTGCAGCTAACGCATTAAG<br>CACTCCGCCTGGGGAGTACGACCGCAAGGTTGAAACTCAAAGGAATTGACGGGGGCCCGCACAAGCGGTGGAGCATG<br>TGGTTTAATTCGAAGCAACGCGAAGAACCTTACCAGGTCTTGACATCCTTTGACCACCTAAGAGATTAGGTTTTCCCTT<br>CGGGGACAAAGAGACAGGTGGTGCATGGCTGTCGTCAGCTCGTGTCGTGAGATGTTGGGTTAAGTCCCGCAACGAGC<br>GCAACCCTTGTTAATAGTTGCCAGCATTAAGTTGGGCACTCTATTGAGACTGCCGGTGACAAACCGGAGGAAGGTGGG<br>GATGACGTCAAGTCATCATGCCCCTTATGACCTGGGCTACACACGTGCTACAATGGGCAGTACAACGAGAAGCGAAC<br>CTGTGAAGGCAAGCGGATCTCTTAAAGCTGTTCTCAGTTCGGACTGTAGGCTGCAACTCGCCTACACGAAGCTGGAAT<br>CGCTAGTAATCGCGGATCAGCACGCCGCGGTGAATACGTTCCCGGGCCTTGTACACACCGCCCGTCACACCATGAGAG<br>TTTGTAACACCCAAAGTCGGTGAGGTAACCTTTGGAGCCAGCCGCCTAAGGTGGGACAGATGATTAGGGTGAAGTCGT<br>AACAAGGTAGCCGTAGGAGAA
	NP8998F	CAAGTCGAGCGAGCTTTGCCTATAGAAATTCTTYGGAATGGACATAKATACWAGCTWKYGGCGGATGGGTGAGTAAC<br>GCGTGGGTAACCTGCCCTTAAGTCTGGGATACCATTTGGAAACAGATGCTAATACCGGATAAAAGCTACTTTCGCATG<br>AAAGAAGTTTAAAAGGCGGCGTAAGCTGTCKCTAAAGGATGGACCTGCGATGCATTAGCTAGTTGGTAAGGTAACGG<br>CTTACCAAGGCGATGATGCATAGCCGAGTTGAGAGACTGATCGGCCACATTGGGACTGAGACACGGCCCAAACTCCT<br>ACGGGAGGCAGCAGTAKGGAATCTTCCACAATGGACGAAAGTCTGATGGAGCAACGCCGCGTGAGTGAAGAAGGTTT<br>TCGGATCGTAAAGCTCTGTTGTTGGTGAAGAAGGATAGAGGTAGTAACTGGCCTTTATTTGACGGTAATCAACCAGAA<br>AGTCACGGCTAACTACGTGCCAGCAGCCGCGGTAATACGTAGGTGGCAWGCGTTGTCCGGATTTATTGGGCGTAAAG<br>CGAGCGCAGGCGGATTGATAAGTCTGATGTGAAAGCCTTCGGCTCAACCGAAGAACTGCATCAGAAACTGTCAATCTT<br>GAGTGCAGAAGAGGAGAGTGGAACTCCATGTGTAGCGGTGGAATGCGTAGATATATGGAAGAACACCAGTGGCGAA<br>KGCGGCTCTCTGKTCTGTAACTGACGCTGAGGCTCGAAAGCATGGGTAGCGAACAGGATTAGATACCCTGGTAKTCCA<br>TGCCGTAAACGATGAGTGCTAAGTGTTGGGAGKTTYCCGCCTCTCAGTGCTGCWCTAACGCATTAAWCACTCCGCCTG

		GGGAGTACGACCGCAAGGTTGAAACTMAAGGAATTGACGSGGGCCCGCMCAGCGGTGGAGCATGTGGTTTAATTCSA<br>AGCACGCKAAKACCTTACMGGYCTTGACATCCTTTGACCACCTAGAGATAGGTTTTCCCTTCSGGACAAGARACAGGT<br>GGKGCAKGCTGYSTCAKCTCGTGYCSTGARATGTTTGGGTAAKTCCCGCATCGAGCGCACCCTTGTTATMGT
	TMPC 9023A	TGGCAGGTGGCGGGTGCTATACATGCAGTCGAGCGAGCTTGCCTATTGAAATTCTTCGGAATGGACATAGATACAAGC<br>TAGCGGCGGATGGGTGAGTAACGCGTGGGTAACCTGCCCTTAAGTCTGGGATACCATTTGGAAACAGATGCTAATACC<br>GGATAAAAGCTACTTTCGCATGAAAGAAGTTTAAAAGGCGGCGTAAGCTGTCGCTAAAGGATGGACCTGCGATGCAT<br>TAGCTAGTTGGTAAGGTAACGGCTTACCAAGGCGATGATGCATAGCCGAGTTGAGAGACTGATCGGCCACATTGGGA<br>CTGAGACACGGCCCAAACTCCTACGGGAGGCAGCAGTAGGGAATCTTCCACAATGGACGCAAGTCTGATGGAGCAAC<br>GCCGCGTGAGTGAAGAAGGTTTTCGGATCGTAAAGCTCTGTTGTTGGTGAAGAAGGATAGAGGTAGTAACTGGCCTTT<br>ATTTGACGGTAATCAACCAGAAAGTCACGGCTAACTACGTGCCAGCAGCCGCGGTAATACGTAGGTGGCAAGCGTTG<br>TCCGGATTTATTGGGCGTAAAGCGAGCGCAGGCGGATTGATAAGTCTGATGTGAAAGCCTTCGGCTCAACCGAAGAA<br>CTGCATCAGAAACTGTCAATCTTGAGTGCAGAAGAGGAGAGTGGAACTCCATGTGTAGCGGTGGAATGCGTAGATAT<br>ATGGAAGAACACCAGTGGCGAAGGCGGCTCTCTGGTCTGTAACTGACGCTGAGGCTCGAAAGCATGGGTAGCGAACA<br>GGATTAGATACCCTGGTAGTCCATGCCGTAAACGATGAGTGCTAAGTGTTGGGAGGTTTCCGCCTCTCAGTGCTGCAG<br>CTAACGCATTAAGCACTCCGCCTGGGGAGTACGACCGCAAGGTTGAAACTCAAAGGAATTGACGGGGGCCCGCACAA<br>GCGGTGGAGCATGTGGTTTAATTCGAAGCAACGCGAAGAACCTTACCAGGTCTTGACATCCTTTGACCACCTAAGAGA<br>TTAGGTTTTCCCTTCGGGGACAAAGAGACAGGTGGTGCATGGCTGTCGTCAGCTCGTGTCGTGAGATGTTGGGTTAAG<br>TCCCGCAACGAGCGCAACCCTTGTTAATAGTTGCCAGCATTAAGTTGGGCACTCTATTGAGACTGCCGGTGACAAACC<br>GGAGGAAGGTGGGGATGACGTCAAGTCATCATGCCCCTTATGACCTGGGCTACACACGTGCTACAATGGGCAGTACA<br>ACGAGAAGCGAACCTGTGAAGGCAAGCGGATCTCTTAAAGCTGTTCTCAGTTCGGACTGTAGGCTGCAACTCGCCTAC<br>ACGAAGCTGGAATCGCTAGTAATCGCGGATCAGCACGCCGCGGTGAATACGTTCCCGGGCCTTGTACACACCGCCCGT<br>CACACCATGAGAGTTTGTAACACCCAAAGTCGGTGAGGTAACCTTTGGAGCCAGCCGCCTAAGGTGGGACAGATGAT<br>TAGGGTGAAGTCGTAACAAGAGCAGGCCCC
Lactobacillus helveticus	Al-Salhi-190	GGGTGAGTAACACGTGGGGAACCTGCCCCATAGTCTGGGATACCACTTGGAAACAGGTGCTAATACCGGATAAGAAA<br>GCAGATCGCATGATCAGCTTATAAAAGGCGGCGTAAGCTGTCGCTATGGGATGGCCCCGCGGTGCATTAGCTAGTTGG<br>TAAGGTAACGGCTTACCAAGGCAATGATGCATAGCCGAGTTGAGAGACTGATCGGCCACATTGGGACTGAGACACGG<br>CCCAAACTCCTACGGGAGGCAGCAGTAGGGAATCTTCCACAATGGACGCAAGTCTGATGGAGCAACGCCGCGTGAGT<br>GAAGAAGGTTTTCGGATCGTAAAGCTCTGTTGTTGGTGAAGAAGGATAGAGGTAGTAACTGGCCTTTATTTGACGGTG<br>ATCAACCAGAAAGTCACGGCTAACTACGTGCCAGCAGCCGCGGTAATACGTAGGTGGCAAGCGTTGTCCGGATTTATT<br>GGGCGTAAAGCGAGCGCAGGCGGAAGAATAAGTCTGATGTGAAAGCCCTCGGCTTAACCGAGGAACTGCATCGGAAA<br>CTGTTTTTCTTGAGTGCAGAAGAGGAGAGTGGAATTCCATGTGTAGCGGTGGAATGCGTAGATATATGGAAGAACACC<br>AGTGGCGAAGGCGACTCTCTGGTCTGCAACTGACGCTGAGGCTCGAAAGCATGGGTAGCGAACAGGATTAGATACCC<br>TGGTAGTCCATGCCGTAAACGATGAGTGCTAAGTGTTGGGAGGTTTCCGCCTCTCAGTGCTGCAGCTAACGCATTAAG<br>CACTCCGCCTGGGGAGTACGACCGCAAGGTTGAAACTCAAAGGAATTGACGGGGGCCCGCACAAGCGGTGGAGCATG

		TGGTTTAATTCGAAGCAACGCGAAGAACCTTACCAGGTCTTGACATC
	DSM 20075	AAAGTTTGATTCTGGCTCAGGACGAACGCTGGCGGCGTGCCTAATACATGCAAGTCGAGCGAGCAGAACCAGCAGAT<br>TTACTTCGGTAATGACGCTGGGGACGCGAGCGGCGGATGGGTGAGTAACACGTGGGGAACCTGCCCCATAGTCTGGG<br>ATACCACTTGGAAACAGGTGCTAATACCGGATAAGAAAGCAGATCGCATGATCAGCTTATAAAAGGCGGCGTAAGCT<br>GTCGCTATGGGATGGCCCCGCGGTGCATTAGCTAGTTGGTAAGGTAACGGCTTACCAAGGCAATGATGCATAGCCGAG<br>TTGAGAGACTGATCGGCCACATTGGGACTGAGACACGGCCCAAACTCCTACGGGAGGCAGCAGTAGGGAATCTTCCA<br>CAATGGACGCAAGTCTGATGGAGCAACGCCGCGTGAGTGAAGAAGGTTTTCGGATCGTAAAGCTCTGTTGTTGGTGAA<br>GAAGGATAGAGGTAGTAACTGGCCTTTATTTGACGGTAATCAACCAGAAAGTCACGGCTAACTACGTGCCAGCAGCC<br>GCGGTAATACGTAGGTGGCAAGCGTTGTCCGGATTTATTGGGCGTAAAGCGAGCGCAGGCGGAAGAATAAGTCTGAT<br>GTGAAAGCCCTCGGCTTAACCGAGGAACTGCATCGGAAACTGTTTTTCTTGAGTGCAGAAGAGGAGAGTGGAATTCCA<br>TGTGTAGCGGTGGAATGCGTAGATATATGGAAGAACACCAGTGGCGAAGGCGACTCTCTGGTCTGCAACTGACGCTG<br>AGGCTCGAAAGCATGGGTAGCGAACAGGATTAGATACCCTGGTAGTCCATGCCGTAAACGATGAGTGCTAAGTGTTG<br>GGAGGTTTCCGCCTCTCAGTGCTGCAGCTAACGCATTAAGCACTCCGCCTGGGGAGTACGACCGCAAGGTTGAAACTC<br>AAAGGAATTGACGGGGGCCCGCACAAGCGGTGGAGCATGTGGTTTAATTCGAAGCAACGCGAAGAACCTTACCAGGT<br>CTTGACATCTAGTGCCATCCTAAGAGATTAGGAGTTCCCTTCGGGGACGCTAAGACAGGTGGTGCATGGCTGTCGTCA<br>GCTCGTGTCGTGAGATGTTGGGTTAAGTCCCGCAACGAGCGCAACCCTTGTTATTAGTTGCCAGCATTAAGTTGGGCA<br>CTCTAATGAGACTGCCGGTGATAAACCGGAGGAAGGTGGGGATGACGTCAAGTCATCATGCCCCTTATGACCTGGGCT<br>ACACACGTGCTACAATGGACAGTACAACGAGAAGCGAGCCTGCGAAGGCAAGCGAATCTCTGAAAGCTGTTCTCAGT<br>TCGGACTGCAGTCTGCAACTCGACTGCACGAAGCTGGAATCGCTAGTAATCGCGGATCAGAACGCCGCGGTGAATAC<br>GTTCCCGGGCCTTGTACACACCGCCCGTCACACCATGGAAGTCTGCAATGCCCAAAGCCGGTGGCCTAACCTTCGGGA<br>AGGAGCCGTCTAAGGCAGGGCAGATGACTGGGGTGAAGTCGTAACAAGGTAGCCGTAGAGAACCTGCGGCTGGATCA<br>CCTCCT
	KHI1	GCAGTCGAGCGAGCAGAACCAGCAGATTTACTTCGGTAATGACGCTGGGGACGCGAGCGGCGGATGGGTGAGTAACA<br>CGTGGGGAACCTGCCCCATAGTCTAGGATACCACTTGGAAACAGGTGCTAATACCGGATAATAAAGCAGATCGCATG<br>ATCAGCTTATAAAAGGCGGCGTAAGCTGTCGCTATGGGATGGCCCCGCGGTGCATTAGCTAGTTGGTAAGGTAACGGC<br>TTACCAAGGCAATGATGCATAGCCGAGTTGAGAGACTGAACGGCCACATTGGGACTGAGACACGGCCCAAACTCCTA<br>CGGGAGGCAGCAGTAGGGAATCTTCCACAATGGACGCAAGTCTGATGGAGCAACGCCGCGTGAGTGAAGAAGGTTTT<br>CGGATCGTAAAGCTCTGTTGTTGGTGAAGAAGGATAGAGGTAGTAACTGGCCTTTATTTGACGGTAATCAACCAGAAA<br>GTCACGGCTAACTACGTGCCAGCAGCCGCGGTAATACGTAGGTGGCAAGCGTTGTCCGGATTTATTGGGCGTAAAGCG<br>AGCGCAGGCGGAAGAATAAGTCTGATGTGAAAGCCCTCGGCTTAACCGAGGAATTGCATCGGAAACTGTTTTTCTTGA<br>GTGCAGAAGAGGAGAGTGGAACTCCATGTGTAGCGGTGGAATGCGTAGATATATGGAAGAACACCAGTGGCGAAGG<br>CGGCTCTCTGGTCTGCAACTGACGCTGAGGCTCGAAAGCATGGGTAGCGAACAGGATTAGATACCCTGGTAGTCCATG<br>CCGTAAACGATGAGTGCTAAGTGTTGGGAGGTTTCCGCCTCTCAGTGCTGCAGCTAACGCATTAAGCACTCCGCCTGG<br>GGAGTACGACCGCAAGGTTGAAACTCAAAGGAATTGACGGGGGCCCGCACAAGCGGTGGAGCATGTGGTTTAATTCG

		AAGCAACGCGAAGAACCTTACCAGGTCTTGACATCTAGTGCCATCCTAAGAGATTAGGAGTTCCCTTCGGGGACGCTA<br>AGACAGGTGGTGCATGGCTGTCGTCAGCTCGTGTCGTGAGATGTTGGGTTAAGTCCCGCAACGAGCGCAACCCTTATT<br>ATTAGTTGCCAGCATTAAGTTGGGCACTCTAATGAGACTGCCGGTGACAAACCGGAGGAAGGTGGGGATGACGTCAA<br>GTCATCATGCCCCTTATGACCTGGGCTACACACGTGCTACAATGGGCAGTACAACGAGAAGCGAGCCTGCGAAGGCA<br>AGCGAATCTCTGAAAGCTGTTCTCAGTTCGGACTGCAGTCTGCAACTCGACTGCACGAAGCTGGAATCGCTAGTAATC<br>GCGGATCAGAACGCCGCGGTGAATACGTTCCCGGGCCTTGTACACACCGCCCGTCACACCATGGAAGTCTGCAATGCC<br>CAAAGCCGGTGGCCTAACCTTCGGGAAGGAGCCGTCTAAGGCAGGGCAGATGACTGGGGTGAAGTCGTAACA
	MJM60419-1	GGCAATGGCGGGGTGCTATACATGCAAGTCGAGCGAGCAGAACCAGCAGATTTACTTCGGTAATGACGCTGGGGACG<br>CGAGCGGCGGATGGGTGAGTAACACGTGGGGAACCTGCCCCATAGTCTAGGATACCACTTGGAAACAGGTGCTAATA<br>CCGGATAATAAAGCAGATCGCATGATCAGCTTATAAAAGGCGGCGTAAGCTGTCGCTATGGGATGGCCCCGCGGTGC<br>ATTAGCTAGTTGGTAAGGTAACGGCTTACCAAGGCAATGATGCATAGCCGAGTTGAGAGACTGAACGGCCACATTGG<br>GACTGAGACACGGCCCAAACTCCTACGGGAGGCAGCAGTAGGGAATCTTCCACAATGGACGCAAGTCTGATGGAGCA<br>ACGCCGCGTGAGTGAAGAAGGTTTTCGGATCGTAAAGCTCTGTTGTTGGTGAAGAAGGATAGAGGTAGTAACTGGCCT<br>TTATTTGACGGTAATCAACCAGAAAGTCACGGCTAACTACGTGCCAGCAGCCGCGGTAATACGTAGGTGGCAAGCGTT<br>GTCCGGATTTATTGGGCGTAAAGCGAGCGCAGGCGGAAGAATAAGTCTGATGTGAAAGCCCTCGGCTTAACCGAGGA<br>ATTGCATCGGAAACTGTTTTTCTTGAGTGCAGAAGAGGAGAGTGGAACTCCATGTGTAGCGGTGGAATGCGTAGATAT<br>ATGGAAGAACACCAGTGGCGAAGGCGGCTCTCTGGTCTGCAACTGACGCTGAGGCTCGAAAGCATGGGTAGCGAACA<br>GGATTAGATACCCTGGTAGTCCATGCCGTAAACGATGAGTGCTAAGTGTTGGGAGGTTTCCGCCTCTCAGTGCTGCAG<br>CTAACGCATTAAGCACTCCGCCTGGGGAGTACGACCGCAAGGTTGAAACTCAAAGGAATTGACGGGGGCCCGCACAA<br>GCGGTGGAGCATGTGGTTTAATTCGAAGCAACGCGAAGAACCTTACCAGGTCTTGACATCTAGTGCCATCCTAAGAGA<br>TTAGGAGTTCCCTTCGGGGACGCTAAGACAGGTGGTGCATGGCTGTCGTCAGCTCGTGTCGTGAGATGTTGGGTTAAG<br>TCCCGCAACGAGCGCAACCCTTATTATTAGTTGCCAGCATTAAGTTGGGCACCTCTAATGAGACTGCCGGTGACAAAC<br>CGGAGGAAGGTGGGGATGACGTCAAGTCATCATGCCCCTTATGACCTGGGCTACACACGTGCTACAATGGGCAGTAC<br>AACGAGAAGCGAGCCTGCGAAGGCAAGCGAATCTCTGAAAGCTGTTCTCAGTTCGGACTGCAGTCTGCAACTCGACT<br>GCACGAAGCTGGAATCGCTAGTAATCGCGGATCAGAACGCCGCGGTGAATACGTTCCCGGGCCTTGTACACACCGCCC<br>GTCACACCATGGAAGTCTGCAATGCCCAAAGCCGGTGGCCTAACCTTCGGGAAGGAGCCGTCTAAGCAGTCAGATGC<br>CTG
	MJM60419-3	GGCAATGGCGGGGTGCTATACATGCAAGTCGAGCGAGCAGAACCAGCAGATTTACTTCGGTAATGACGCTGGGGACG<br>CGAGCGGCGGATGGGTGAGTAACACGTGGGGAACCTGCCCCATAGTCTAGGATACCACTTGGAAACAGGTGCTAATA<br>CCGGATAATAAAGCAGATCGCATGATCAGCTTATAAAAGGCGGCGTAAGCTGTCGCTATGGGATGGCCCCGCGGTGC<br>ATTAGCTAGTTGGTAAGGTAACGGCTTACCAAGGCAATGATGCATAGCCGAGTTGAGAGACTGAACGGCCACATTGG<br>GACTGAGACACGGCCCAAACTCCTACGGGAGGCAGCAGTAGGGAATCTTCCACAATGGACGCAAGTCTGATGGAGCA<br>ACGCCGCGTGAGTGAAGAAGGTTTTCGGATCGTAAAGCTCTGTTGTTGGTGAAGAAGGATAGAGGTAGTAACTGGCCT<br>TTATTTGACGGTAATCAACCAGAAAGTCACGGCTAACTACGTGCCAGCAGCCGCGGTAATACGTAGGTGGCAAGCGTT

		GTCCGGATTTATTGGGCGTAAAGCGAGCGCAGGCGGAAGAATAAGTCTGATGTGAAAGCCCTCGGCTTAACCGAGGA<br>ATTGCATCGGAAACTGTTTTTCTTGAGTGCAGAAGAGGAGAGTGGAACTCCATGTGTAGCGGTGGAATGCGTAGATAT<br>ATGGAAGAACACCAGTGGCGAAGGCGGCTCTCTGGTCTGCAACTGACGCTGAGGCTCGAAAGCATGGGTAGCGAACA<br>GGATTAGATACCCTGGTAGTCCATGCCGTAAACGATGAGTGCTAAGTGTTGGGAGGTTTCCGCCTCTCAGTGCTGCAG<br>CTAACGCATTAAGCACTCCGCCTGGGGAGTACGACCGCAAGGTTGAAACTCAAAGGAATTGACGGGGGCCCGCACAA<br>GCGGTGGAGCATGTGGTTTAATTCGAAGCAACGCGAAGAACCTTACCAGGTCTTGACATCTAGTGCCATCCTAAGAGA<br>TTAGGAGTTCCCTTCGGGGACGCTAAGACAGGTGGTGCATGGCTGTCGTCAGCTCGTGTCGTGAGATGTTGGGTTAAG<br>TCCCGCAACGAGCGCAACCCTTATTATTAGTTGCCAGCATTAAGTTGGGCACCTCTAATGAGACTGCCGGTGACAAAC<br>CGGAGGAAGGTGGGGATGACGTCAAGTCATCATGCCCCTTATGACCTGGGCTACACACGTGCTACAATGGGCAGTAC<br>AACGAGAAGCGAGCCTGCGAAGGCAAGCGAATCTCTGAAAGCTGTTCTCAGTTCGGACTGCAGTCTGCAACTCGACT<br>GCACGAAGCTGGAATCGCTAGTAATCGCGGATCAGAACGCCGCGGTGAATACGTTCCCGGGCCTTGTACACACCGCCC<br>GTCACACCATGGAAGTCTGCAATGCCCAAAGCCGGTGGCCTAACCTTCGGGAAGGAGCCGTCTAAGCAGTCAGATGC<br>CTG
Lactobacillus johnsonii	ATCC 33200	GGCGGCGTGCCTAATACATGCAAGTCGAGCGAGCTTGCCTAGATGATTTAGTGCTTGCACTAAATGAAACTAGATAC<br>AAGCGAGCGGCGGACGGGTGAGTAACACGTGGGTAACCTGCCCAAGAGACTGGGATAACACCTGGAAACAGATGCT<br>AATACCGGATAACAACACTAGACGCATGTCTAGAGTTTGAAAGATGGTTCTGCTATCACTCTTGGATGGACCTGCGGT<br>GCATTAGCTAGTTGGTAAGGTAATGGCTTACCAAGGCGATGATGCATAGCCGAGTTGAGAGACTGATCGGCCACATTG<br>GGACTGAGACACGGCCCAAACTCCTACGGGAGGCAGCAGTAGGGAATCTTCCACAATGGACGAAAGTCTGATGGAGC<br>AACGCCGCGTGAGTGAAGAAGGGTTTCGGCTCGTAAAGCTCTGTTGGTAGTGAAGAAAGATAGAGGTAGTAACTGGC<br>CTTTATTTGACGGTAATTACTTAGAAAGTCACGGCTAACTACGTGCCAGCAGCCGCGGTAATACGTAGGTGGCAAGCG<br>TTGTCCGGATTTATTGGGCGTAAAGCGAGTGCAGGCGGTTCAATAAGTCTGATGTGAAACGCTTCGGCTCAACCGGAG<br>AATTGCATCAGAAACTGTTGAACTTGAGTGCAGAAGAGGAGAGTGGAACTCCATGTGTAGCGGTGGAATGCGTAGAT<br>ATATGGAAGAACACCAGTGGCGAAGGCGGCTCTCTGGTCTGCAACTGACGCTGAGGCTCGAAAGCATGGGTAGCGAA<br>CAGGATTAGATACCCTGGTAGTCCATGCCGTAAACGATGAGTGCTAAGTGTTGGGAGGTTTCCGCCTCTCAGTGCTGC<br>AGCTAACGCATTAAGCACTCCGCCTGGGGAGTACGACCGCAAGGTTGAAACTCAAAGGAATTGACGGGGGCCCGCAC<br>AAGCGGTGGAGCATGTGGTTTAATTCGAAGCAACGCGAAGAACCTTACCAGGTCTTGACATCCAGTGCAAACCTAAG<br>AGATTAGGTGTTCCCTTCGGGGACGCTGAGACAGGTGGTGCATGGCTGTCGTCAGCTCGTGTCATGAGATGTTGGGTT<br>AAGTCCCGCAACGAGCGCAACCCTTGTCATTAGTTGCCATCATTAAGTTGGGCACTCTAATGAGACTGCCGGTGACAA<br>ACCGGAGGAAGGTGGGGATGACGTCAAGTCATCATGCCCCTTATGACCTGGGCTACACACGTGCTACAATGGACGGT<br>ACAACGAGAAGCGAACCTGCGAAGGCAAGCGGATCTCTTAAAGCCGTTCTCAGTTCGGACTGTAGGCTGCAACTCGC<br>CTACACGAAGCTGGAATCGCTAGTAATCGCGGATCAGCACGCCGCGGTGAATACGTTCCCGGGCCTTGTACACACCGC<br>CCGTCACACCATGAGAGTCTGTAACACCCAAAGCCGGTGGGATAACCTTTATAGGAGTCAGCCGTCTAAGGTAGGAC<br>AGATGATTAGGGTGAA
	LJ_c1-2	TACATGCAAGTCGAGCGAGCTTGCCTAGATGATTTAGTGCTTGCACTAAATGAAACTAGATACAAGCGAGCGGCGGA

		CGGGTGAGTAACACGTGGGTAACCTGCCCAAGAGACTGGGATAACACCTGGAAACAGATGCTAATACCGGATAACAA<br>CACTAGACGCATGTCTAGAGTTTGAAAGATGGTTCTGCTATCACTCTTGGATGGACCTGCGGTGCATTAGCTAGTTGGT<br>AAGGTAACGGCTTACCAAGGCAATGATGCATAGCCGAGTTGAGAGACTGATCGGCCACATTGGGACTGAGACACGGC<br>CCAAACTCCTACGGGAGGCAGCAGTAGGGAATCTTCCACAATGGACGAAAGTCTGATGGAGCAACGCCGCGTGAGTG<br>AAGAAGGGTTTCGGCTCGTAAAGCTCTGTTGGTAGTGAAGAAAGATAGAGGTAGTAACTGGCCTTTATTTGACGGTAA<br>TTACTTAGAAAGTCACGGCTAACTACGTGCCAGCAGCCGCGGTAATACGTAGGTGGCAAGCGTTGTCCGGATTTATTG<br>GGCGTAAAGCGAGTGCAGGCGGTTCAATAAGTCTGATGTGAAAGCCTTCGGCTCAACCGGAGAATTGCATCAGAAAC<br>TGTTGAACTTGAGTGCAGAAGAGGAGAGTGGAACTCCATGTGTAGCGGTGGAATGCGTAGATATATGGAAGAACACC<br>AGTGGCGAAGGCGGCTCTCTGGTCTGTAACTGACGCTGAGGCTCGAAAGCATGGGTAGCGAACAGGATTAGATACCC<br>TGGTAGTCCATGCCGTAAACGATGAGTGCTAAGTGTTGGGAGGTTTCCGCCTCTCAGTGCTGCAGCTAACGCATTAAG<br>CACTCCGCCTGGGGAGTACGACCGCAAGGTTGAAACTCAAAGGAATTGACGGGGGCCCGCACAAGCGGTGGAGCATG<br>TGGTTTAATTCGAAGCAACGCGAAGAACCTTACCAGGTCTTGACATCCAGTGCAAACCTAAGAGATTAGGTGTTCCCT<br>TCGGGGACGCTGAGACAGGTGGTGCATGGCTGTCGTCAGCTCGTGTCGTGAGATGTTGGGTTAAGTCCCGCAACGAGC<br>GCAACCCTTGTCATTAGTTGCCATCATTAAGTTGGGCACTCTAATGAGACTGCCGGTGACAAACCGGAGGAAGGTGGG<br>GATGACGTCAAGTCATCATGCCCCTTATGACCTGGGCTACACACGTGCTACAATGGACGGTACAACGAGAAGCAAAC<br>CTGCGAAGGCAAGCGGATCTCTTAAAGCCGTTCTCAGTTCGGACTGTAGGCTGCAACTCGCCTACACGAAGCTGGAAT<br>CGCTAGTAATCGCGGATCAGCACGCCGCGGTGAATACGTTCCCGGGCCTTGTACACACCGCCCGTCACACCATGAGAG<br>TCTGTAACACCCAAAGCCGGTGGGATAACCTTTATAGGAGTCAG
	LJ_c3-4	GCGGCGTGCCTAATACATGCAAGTCGAGCGAGCTTGCCTAGATGATTTAGTGCTTGCACTAAATGAAACTAGATACA<br>AGCGAGCGGCGGACGGGTGAGTAACACGTGGGTAACCTGCCCAAGAGACTGGGATAACACCTGGAAACAGATGCTA<br>ATACCGGATAACAACACTAGACGCATGTCTAGAGTTTGAAAGATGGTTCTGCTATCACTCTTGGATGGACCTGCGGTG<br>CATTAGCTAGTTGGTAAGGTAACGGCTTACCAAGGCAATGATGCATAGCCGAGTTGAGAGACTGATCGGCCACATTGG<br>GACTGAGACACGGCCCAAACTCCTACGGGAGGCAGCAGTAGGGAATCTTCCACAATGGACGAAAGTCTGATGGAGCA<br>ACGCCGCGTGAGTGAAGAAGGGTTTCGGCTCGTAAAGCTCTGTTGGTAGTGAAGAAAGATAGAGGTAGTAACTGGCC<br>TTTATTTGACGGTAATTACTTAGAAAGTCACGGCTAACTACGTGCCAGCAGCCGCGGTAATACGTAGGTGGCAAGCGT<br>TGTCCGGATTTATTGGGCGTAAAGCGAGTGCAGGCGGTTCAATAAGTCTGATGTGAAAGCCTTCGGCTCAACCGGAGA<br>ATTGCATCAGAAACTGTTGAACTTGAGTGCAGAAGAGGAGAGTGGAACTCCATGTGTAGCGGTGGAATGCGTAGATA<br>TATGGAAGAACACCAGTGGCGAAGGCGGCTCTCTGGTCTGTAACTGACGCTGAGGCTCGAAAGCATGGGTAGCGAAC<br>AGGATTAGATACCCTGGTAGTCCATGCCGTAAACGATGAGTGCTAAGTGTTGGGAGGTTTCCGCCTCTCAGTGCTGCA<br>GCTAACGCATTAAGCACTCCGCCTGGGGAGTACGACCGCAAGGTTGAAACTCAAAGGAATTGACGGGGGCCCGCACA<br>AGCGGTGGAGCATGTGGTTTAATTCGAAGCAACGCGAAGAACCTTACCAGGTCTTGACATCCAGTGCAAACCTAAGA<br>GATTAGGTGTTCCCTTCGGGGACGCTGAGACAGGTGGTGCATGGCTGTCGTCAGCTCGTGTCGTGAGATGTTGGGTTA<br>AGTCCCGCAACGAGCGCAACCCTTGTCATTAGTTGCCATCATTAAGTTGGGCACTCTAATGAGACTGCCGGTGACAAA<br>CCGGAGGAAGGTGGGGATGACGTCAAGTCATCATGCCCCTTATGACCTGGGCTACACACGTGCTACAATGGACGGTA

		CAACGAGAAGCGAACCTGCGAAGGCAAGCGGATCTCTTAAAGCCGTTCTCAGTTCGGACTGTAGGCTGCAACTCGCCT ACACGAAGCTGGAATCGCTAGTAATCGCGGATCAGCACGCCGCGGTGAATACGTTCCCGGGCCTTGTACACACCGCCC GTCACACCATGAGAGTCTGTAACACCCAAAGCCGGTGGGATAACCTTTATAGGAGTCAGCCG
	LJ_c3-6	TCGAGCGAGCTTGCCTAGATGATTTTAGTGCTTGCACTAAATGAAACTAGATACAAGCGAGCGGCGGACGGGTGAGT AACACGTGGGTAACCTGCCCAAGAGACTGGGATAACACCTGGAAACAGATGCTAATACCGGATAACAACACTAGACG CATGTCTAGAGTTTGAAAGATGGTTCTGCTATCACTCTTGGATGGACCTGCGGTGCATTAGCTAGTTGGTAAGGTAAC GGCTTACCAAGGCAATGATGCATAGCCGAGTTGAGAGACTGATCGGCCACATTGGGACTGAGACACGGCCCAAACTC CTACGGGAGGCAGCAGTAGGGAATCTTCCACAATGGACGAAAGTCTGATGGAGCAACGCCGCGTGAGTGAAGAAGG GTTTCGGCTCGTAAAGCTCTGTTGGTAGTGAAGAAAGATAGAGGTAGTAACTGGCCTTTATTTGACGGTAATTACTTA GAAAGTCACGGCTAACTACGTGCCAGCAGCCGCGGTAATACGTAGGTGGCAAGCGTTGTCCGGATTTATTGGGCGTAA AGCGAGTGCAGGCGGTTCAATAAGTCTGATGTGAAAGCCTTCGGCTCAACCGGAGAATTGCATCAGAAACTGTTGAA CTTGAGTGCAGAAGAGGAGAGTGGAACTCCATGTGTAGCGGTGGAATGCGTAGATATATGGAAGAACACCAGTGGCG AAGGCGGCTCTCTGGTCTGCAACTGACGCTGAGGCTCGAAAGCATGGGTAGCGAACAGGATTAGATACCCTGGTAGT CCATGCCGTAAACGATGAGTGCTAAGTGTTGGGAGGTTTCCGCCTCTCAGTGCTGCAGCTAACGCATTAAGCACTCCG CCTGGGGAGTACGACCGCAAGGTTGAAACTCAAAGGAATTGACGGGGGCCCGCACAAGCGGTGGAGCATGTGGTTTA ATTCGAAGCAACGCGAAGAACCTTACCAGGTCTTGACATCCAGTGCAAACCTAAGAGATTAGGTGTTCCCTTCGGGGA CGCTGAGACAGGTGGTGCATGGCTGTCGTCAGCTCGTGTCGTGAGATGTTGGGTTAAGTCCCGCAACGAGCGCAACCC TTGTCATTAGTTGCCATCATTAAGTTGGGCACTCTAATGAGACTGCCGGTGACAAACCGGAGGAAGGTGGGGATGACG TCAAGTCATCATGCCCCTTATGACCTGGGCTACACACGTGCTACAATGGACGGTACAACGAGAAGCAAACCTGCGAA GGCAAGCGGATCTCTTAAAGCCGTTCTCAGTTCGGACTGTAGGCTGCAACTCGCCTACACGAAGCTGGAATCGCTAGT AATCGCGGATCAGCACGCCGCGGTGAATACGTTCCCGGGCCTTGTACACACCGCCCGTCACACCATGAGAGTCTGTAA CACCCAAAGCCGGTGGGAT
	LJ_mika1	AGTCGAGCGAGCTTGCCTAGATGATTTTGGTGCTTGCACTAAATGAAACTAGATACAAGCGAGCGGCGGACGGGTGA GTAACACGTGGGTAACCTGCCCAAGAGACTGGGATAACACCTGGAAACAGATGCTAATACCGGATAACAACACTAGA CGCATGTCTAGAGTTTGAAAGATGGTTCTGCTATCACTCTTGGATGGACCTGCGGTGCATTAGCTAGTTGGTAAGGTA ACGGCTTACCAAGGCAATGATGCATAGCCGAGTTGAGAGACTGATCGGCCACATTGGGACTGAGACACGGCCCAAAC TCCTACGGGAGGCAGCAGTAGGGAATCTTCCACAATGGACGAAAGTCTGATGGAGCAACGCCGCGTGAGTGAAGAAG GGTTTCGGCTCGTAAAGCTCTGTTGGTAGTGAAGAAAGATAGAGGTAGTAACTGGCCTTTATTTGACGGTAATTACTT AGAAAGTCACGGCTAACTACGTGCCAGCAGCCGCGGTAATACGTAGGTGGCAAGCGTTGTCCGGATTTATTGGGCGTA AAGCGAGTGCAGGCGGTTCAATAAGTCTGATGTGAAAGCCTTCGGCTCAACCGGAGAATTGCATCAGAAACTGTTGA ACTTGAGTGCAGAAGAGGAGAGTGGAACTCCATGTGTAGCGGTGGAATGCGTAGATATATGGAAGAACACCAGTGGC GAAGGCGGCTCTCTGGTCTGCAACTGACGCTGAGGCTCGAAAGCATGGGTAGCGAACAGGATTAGATACCCTGGTAG TCCATGCCGTAAACGATGAGTGCTAAGTGTTGGGAGGTTTCCGCCTCTCAGTGCTGCAGCTAACGCATTAAGCACTCC GCCTGGGGAGTACGACCGCAAGGTTGAAACTCAAAGGAATTGACGGGGGCCCGCACAAGCGGTGGAGCATGTGGTTT

		AATTCGAAGCAACGCGAAGAACCTTACCAGGTCTTGACATCCAGTGCAAACCTAAGAGATTAGGTGTTCCCTTCGGGG<br>ACGCTGAGACAGGTGGTGCATGGCTGTCGTCAGCTCGTGTCGTGAGATGTTGGGTTAAGTCCCGCAACGAGCGCAACC<br>CTTGTCATTAGTTGCCATCATTAAGTTGGGCACTCTAATGAGACTGCCGGTGACAAACCGGAGGAAGGTGGGGATGAC<br>GTCAAGTCATCATGCCCCTTATGACCTGGGCTACACACGTGCTACAATGGACGGTACAACGAGAAGCGAACCTGCGA<br>AGGCAAGCGGATCTCTTAAAGCCGTTCTCAGTTCGGACTGTAGGCTGCAACTCGCCTACACGAAGCTGGAATCGCTAG<br>TAATCGCGGATCAGCACGCCGCGGTGAATACGTTCCCGGGCCTTGTACACACCGCCCGTCACACCATGAGAGTCTGTA<br>ACACCCAAAGCCGGTGGGAT
Lactobacillus kefiranofaciens	ATCC 43761	AGAGTTTGATCATGGCTCAGGACGAACGCTGGCGGCGTGCCTAATACATGCAAGTCGAGCGAGCGGAACCAGCAGAA<br>TCACTTCGGTGAGGACGCTGGGAAAGCGAGCGGCGGATGGGTGAGTAACACGTGGGGAACCTGCCCTTAAGTCTGGG<br>ATACCACTTGGAAACAGGTGCTAATACCGGATAAGAAAGCAGTTCGCATGAACAGCTTTTAAAAGGCGGCGCAAGCT<br>GTCGCTAAAGGATGGACCCGCGGTGCATTAGCTAGTTGGTAAGGTAACGGCCTACCAAGGCAGTGATGCATAGCCGA<br>GTTGAGAGACTGATCGGCCACATTGGGACTGAGACACGGCCCAAACTCCTACGGGAGGCAGCAGTAGGGAATCTTCC<br>ACAATGGACGCAAGTCTGATGGAGCAACGCCGCGTGAGTGAAGAAGGTTTTCGGACCGTAAAGCTCTGTTGTTGGTG<br>AAGAAGGATAGAGGTAGTAACTGGCCTTTATTTGACGGTAATCAACCAGAAAGTCACGGCTAACTACGTGCCAGCAG<br>CCGCGGTAATACGTAGGTGGCAAGCGTTGTCCGGATTTATTGGGCGTAAAGCGAGCGCAGGCGGAAGAATAAGTCTG<br>ATGTGAAAGCCCTCGGCTTAACCGAGGAATTGCATCGGAAACTGTTTTTCTTGAGTGCAGAAGAGGAGAGTAGAACTC<br>CATGTGTAGCGGTGGAATGCGTAGATATATGGAAGAATACCAGTGGCGAAGGCGGCTCTCTGGTCTGCAACTGACGCT<br>GAGGCTCGAAAGCATGGGTAGCGAACAGGATTAGATACCCTGGTAGTCCATGCCGTAAACGATGAGTGCTAAGTGTT<br>GGGAGGCTTCCGCCTCTCAGTGCTGCAGCTAACGCATTAAGCACTCCGCCTGGGGAGTACGACCGCAAGGTTGAAACT<br>CAAAGGAATTGACGGGGGCCCGCACAAGCGGTGGAGCATGTGGTTTAATTCGAAGCAACGCGAAGAACCTTACCAGG<br>TCTTGACATCTAGTGCCATTTGTAGAGATACAAAGTTCCCTTCGGGGACGCTAAGACAGGTGGTGCATGGCTGTCGTC<br>AGCTCGTGTCGTGAGATGTTGGGTTAAGTCCCGCAACGAGCGCAACCCTTGTTATTAGTTGCCAGCATTAAGTTGGGC<br>ACTCTAATGAGACTGCCGGTGACAAACCGGAGGAAGGTGGGGATGACGTCAAGTCATCATGCCCCTTATGACCTGGG<br>CTACACACGTGCTACAATGGGCAGCACAACGAGCAGCGAGCCTGCAAAGGCAAGCAAATCTCTGAAAGCTGTTCTCA<br>GTTCGGACTGCAGTCTGCAACTCGACTGCACGAAGCTGGAATCGCTAGTAATCGCGGATCAGCACGCCGCGGTGAATA<br>CGTTCCCGGGCCTTGTACACACCGCCCGTCACACCATGGGAGTCTGCAATGCCCAAAGCCGGTGGCCTAACCGCAAGG<br>AAGGAGCCGTCTAAGGCAGGGCAGATGACTGGGGTGAAGTCGTAACAAGGTAGCCGTAGGAGAACCTGCGGTTGGAT<br>CA
	LMG 19149	GACGAACGCTGGCGGCGTGCCTAATACATGCAAGTCGAGCGAGCGGAACCAGCAGAATCACTTCGGTGAGGACGCTG<br>GGAAAGCGAGCGGCGGATGGGTGAGTAACACGTGGGGAACCTGCCCTTAAGTCTGGGATACCACTTGGAAACAGGTG<br>CTAATACCGGATAAGAAAGCAGTTCGCATGAACAGCTTTTAAAAGGCGGCGCAAGCTGTCGCTAAAGGATGGACCCG<br>CGGTGCATTAGCTAGTTGGTAAGGTAACGGCCTACCAAGGCAGTGATGCATAGCCGAGTTGAGAGACTGATCGGCCA<br>CATTGGGACTGAGACACGGCCCAAACTCCTACGGGAGGCAGCAGTAGGGAATCTTCCACAATGGACGCAAGTCTGAT<br>GGAGCAACGCCGCGTGAGTGAAGAAGGTTTTCGGACCGTAAAGCTCTGTTGTTGGTGAAGAAGGATAGAGGTAGTAA

		CTGGCCTTTATTTGACGGTAATCAACCAGAAAGTCACGGCTAACTACGTGCCAGCAGCCGCGGTAATACGTAGGTGGC<br>AAGCGTTGTCCGGATTTATTGGGCGTAAAGCGAGCGCAGGCGGAAGAATAAGTCTGATGTGAAAGCCCTCGGCTTAA<br>CCGAGGAATTGCATCGGAAACTGTTTTTCTTGAGTGCAGAAGAGGAGAGTAGAACTCCATGTGTAGCGGTGGAATGC<br>GTAGATATATGGAAGAATACCAGTGGCGAAGGCGGCTCTCTGGTCTGCAACTGACGCTGAGGCTCGAAAGCATGGGT<br>AGCGAACAGGATTAGATACCCTGGTAGTCCATGCCGTAAACGATGAGTGCTAAGTGTTGGGAGGCTTCCGCCTCTCAG<br>TGCTGCAGCTAACGCATTAAGCACTCCGCCTGGGGAGTACGACCGCAAGGTTGAAACTCAAAGGAATTGACGGGGGC<br>CCGCACAAGCGGTGGAGCATGTGGTTTAATTCGAAGCAACGCGAAGAACCTTACCAGGTCTTGACATCTAGTGCCATT<br>TGTAGAGATACAAAGTTCCCTTCGGGGACGCTAAGACAGGTGGTGCATGGCTGTCGTCAGCTCGTGTCGTGAGATGTT<br>GGGTTAAGTCCCGCAACGAGCGCAACCCTTGTTATTAGTTGCCAGCATTAAGTTGGGCACTCTAATGAGACTGCCGGT<br>GACAAACCGGAGGAAGGTGGGGATGACGTCAAGTCATCATGCCCCTTATGACCTGGGCTACACACGTGCTACAATGG<br>GCAGCACAACGAGCAGCGAGCCTGCAAAGGCAAGCAAATCTCTGAAAGCTGTTCTCAGTTCGGACTGCAGTCTGCAA<br>CTCGACTGCACGAAGCTGGAATCGCTAGTAATCGCGGATCAGCACGCCGCGGTGAATACGTTCCCGGGCCTTGTACAC<br>ACCGCCCGTCACACCATGGGAGTCTGCAATGCCCAAAGCCGGTGGCCTAACCGCAAGGAAGGAGCCGTCTAAGGCAG<br>GGCAGATGACTGGGGTGAAGTCGTAACAAGGTAGCCGTAGGAGAACC
	TMPC 4D128	GTGCACCGGGCGGCGTGCTATACATGCAAGTCGAGCGAGCGGAACCAGCAGAATCACTTCGGTGAGGACGCTGGGAA<br>AGCGAGCGGCGGATGGGTGAGTAACACGTGGGGAACCTGCCCTTAAGTCTGGGATACCACTTGGAAACAGGTGCTAA<br>TACCGGATAAGAAAGCAGTTCGCATGAACAGCTTTTAAAAGGCGGCGCAAGCTGTCGCTAAAGGATGGACCCGCGGT<br>GCATTAGCTAGTTGGTAAGGTAACGGCCTACCAAGGCAGTGATGCATAGCCGAGTTGAGAGACTGATCGGCCACATT<br>GGGACTGAGACACGGCCCAAACTCCTACGGGAGGCAGCAGTAGGGAATCTTCCACAATGGACGCAAGTCTGATGGAG<br>CAACGCCGCGTGAGTGAAGAAGGTTTTCGGACCGTAAAGCTCTGTTGTTGGTGAAGAAGGATAGAGGTAGTAACTGG<br>CCTTTATTTGACGGTAATCAACCAGAAAGTCACGGCTAACTACGTGCCAGCAGCCGCGGTAATACGTAGGTGGCAAGC<br>GTTGTCCGGATTTATTGGGCGTAAAGCGAGCGCAGGCGGAAGAATAAGTCTGATGTGAAAGCCCTCGGCTTAACCGA<br>GGAATTGCATCGGAAACTGTTTTTCTTGAGTGCAGAAGAGGAGAGTAGAACTCCATGTGTAGCGGTGGAATGCGTAG<br>ATATATGGAAGAATACCAGTGGCGAAGGCGGCTCTCTGGTCTGCAACTGACGCTGAGGCTCGAAAGCATGGGTAGCG<br>AACAGGATTAGATACCCTGGTAGTCCATGCCGTAAACGATGAGTGCTAAGTGTTGGGAGGCTTCCGCCTCTCAGTGCT<br>GCAGCTAACGCATTAAGCACTCCGCCTGGGGAGTACGACCGCAAGGTTGAAACTCAAAGGAATTGACGGGGGCCCGC<br>ACAAGCGGTGGAGCATGTGGTTTAATTCGAAGCAACGCGAAGAACCTTACCAGGTCTTGACATCTAGTGCCATTTGTA<br>GAGATACAAAGTTCCCTTCGGGGACGCTAGACAGGTGGTGCATGGCTGTCGTCAGCTCGTGTCGTGAGATGTGGGTAG<br>TCCGCACGAGCGCACCCTTGTTATAGTGCAGCATAGTGGGCCCTCTATGAGACTGCGGTGACACGGAGAGGTGGGGAT<br>GACGTCAGTCATCATGCCCTTATGACTGGCTACCACGTGCTAAATGGCAGCCACAGCACGGACCTGCAGCAGCATCTG<br>GAGCGTTTCAGTCGACGACGACTCACTGCAACTGAATCGTGAATCCGATACAACGCGCGGGAATCTTCGCTGTTACCT<br>CCGCCGCTCACACTG
	TMPC 4D176	GTGCAAGGGCGGCGTGCTATACATGCAAGTCGAGCGAGTGGAACCAGCAGAATCACTTCGGTGAGGACGCTGGGAAA<br>GCGAGCGGCGGATGGGTGAGTAACACGTGGGGAACCTGCCCTTAAGTCTGGGATACCACTTGGAAACAGGTGCTAAT

		ACCGGATAAGAAAGCAGTTCGCATGAACAGCTTTTAAAAGGCGGCGCAAGCTGTCGCTAAAGGATGGACCCGCGGTG<br>CATTAGCTAGTTGGTAAGGTAACGGCCTACCAAGGCAGTGATGCATAGCCGAGTTGAGAGACTGATCGGCCACATTG<br>GGACTGAGACACGGCCCAAACTCCTACGGGAGGCAGCAGTAGGGAATCTTCCACAATGGACGCAAGTCTGATGGAGC<br>AACGCCGCGTGAGTGAAGAAGGTTTTCGGACCGTAAAGCTCTGTTGTTGGTGAAGAAGGATAGAGGTAGTAACTGGC<br>CTTTATTTGACGGTAATCAACCAGAAAGTCACGGCTAACTACGTGCCAGCAGCCGCGGTAATACGTAGGTGGCAAGCG<br>TTGTCCGGATTTATTGGGCGTAAAGCGAGCGCAGGCGGAAGAATAAGTCTGATGTGAAAGCCCTCGGCTTAACCGAG<br>GAATTGCATCGGAAACTGTTTTTCTTGAGTGCAGAAGAGGAGAGTAGAACTCCATGTGTAGCGGTGGAATGCGTAGAT<br>ATATGGAAGAATACCAGTGGCGAAGGCGGCTCTCTGGTCTGCAACTGACGCTGAGGCTCGAAAGCATGGGTAGCGAA<br>CAGGATTAGATACCCTGGTAGTCCATGCCGTAAACGATGAGTGCTAAGTGTTGGGAGGCTTCCGCCTCTCAGTGCTGC<br>AGCTAACGCATTAAGCACTCCGCCTGGGGAGTACGACCGCAAGGTTGAAACTCAAAGGAATTGACGGGGGCCCGCAC<br>AAGCGGTGGAGCATGTGGGTTTAATTCGAAGCAACGCGAAGAACCTTACCAGGTCTTGACATCTAGTGCCATTTGTAG<br>AGATACAAAGTTCCCTTCGGGGACGCTAAGACAGGTGTGCATGGCTGTCGTCAGCTCGTGTCGTGAGATGTGGGTAAG<br>TCCGCACGAGCGCAACCTGTATAGTGCAGCATAGTGGCACTCTAATGAGAACTGCGTGACAACGGAGAGGTGGATGA<br>CGTCAGTCATCATGCCCTTATGACTGGCTACACACGTGCTACAATGGTACACAACAGGCAGCGAGCCTGCCAGGCAAG<br>CCA
	TMPC 4D178	AAAGCAAATTGCGGGCGTGCCTATACATGCAAGTCGAGCGAGCGGAACCAGCAGAATCACTTCGGTGAGGACGCTGG<br>GAAAGCGAGCGGCGGATGGGTGAGTAACACGTGGGGAACCTGCCCTTAAGTCTGGGATACCACTTGGAAACAGGTGC<br>TAATACCGGATAAGAAAGCAGTTCGCATGAACAGCTTTTAAAAGGCGGCGCAAGCTGTCGCTAAAGGATGGACCCGC<br>GGTGCATTAGCTAGTTGGTAAGGTAACGGCCTACCAAGGCAGTGATGCATAGCCGAGTTGAGAGACTGATCGGCCAC<br>ATTGGGACTGAGACACGGCCCAAACTCCTACGGGAGGCAGCAGTAGGGAATCTTCCACAATGGACGCAAGTCTGATG<br>GAGCAACGCCGCGTGAGTGAAGAAGGTTTTCGGACCGTAAAGCTCTGTTGTTGGTGAAGAAGGATAGAGGTAGTAAC<br>TGGCCTTTATTTGACGGTAATCAACCAGAAAGTCACGGCTAACTACGTGCCAGCAGCCGCGGTAATACGTAGGTGGCA<br>AGCGTTGTCCGGATTTATTGGGCGTAAAGCGAGCGCAGGCGGAAGAATAAGTCTGATGTGAAAGCCCTCGGCTTAAC<br>CGAGGAATTGCATCGGAAACTGTTTTTCTTGAGTGCAGAAGAGGAGAGTAGAACTCCATGTGTAGCGGTGGAATGCGT<br>AGATATATGGAAGAATACCAGTGGCGAAAGGCGGCTCTCTGGTCTGCAACTGACGCTGAGGCTCGAAAGCATGGGTA<br>GCGAACAGGATTAGATACCCTGGTAGTCCATGCCGTAACGATGAGTGCTAAGTGTTGGGAGGCTTCCGCCTCTCAGTG<br>CTGCAGCTAACGCATTAAGCACTCCGCCTGGGAGTACGACCCGCAAGGTTGAAACTCAAAGGAATTGACGGGGCCCG<br>CACCACCGGTGGAGCATGGGGTTTATTCCGAAGCAACGCGAAAAACCTTACCAGTCCTGACTTCTAGTGCCATTTGGA<br>AATTACCAAAGTTCCCTTCGGACGCTCAAACACGAGGTGCATGGACTGTCCTCCGCTCGAGTCAGCAAAATGTGGTTA<br>ACTCCCGCACGAACGCAACCTCTGGTAATGATGTGCAGGATAAGTGGGCCATCTATGGAAACTGGCCGGTGAACATA<br>CACCGGGAAGAATGGTGGCATGAACGCTCAAAGT
Lactobacillus ultunensis	Lactobacillus ultunensis	GTTTGATCCTGGCTCAGGACGAACGCTGGCGGCGTGCCTAATACATGCAAGTCGAGCGAGCGGAACCAGCAGATCTG<br>CTTCGGCAGTGACGCTGGGAAAGCGAGCGGCGGATGGGTGAGTAACACGTGGGGAACCTGCCCCAAAGTCTGGGATA<br>CCACTTGGAAACAGGTGCTAATACCGGATAAGAAAGCAGATCGCATGATCAGCTTTTAAAAGGCGGCGTAAGCTGTC

		GCTATGGGATGGCCCCGCGGTGCATTAGCTAGTTGGTAGAGTAACGGCCTACCAAGGCAATGATGCATAGCCGAGTTG<br>AGAGACTGATCGGCCACATTGGGACTGAGACACGGCCCAAACTCCTACGGGAGGCAGCAGTAGGGAATCTTCCACAA<br>TGGACGCAAGTCTGATGGAGCAACGCCGCGTGAGTGAAGAAGGTTTTCGGATCGTAAAGCTCTGTTGTTGGTGAAGA<br>AGGATAGAGGTAGTAACTGGCCTTTATTTGACGGTAATCAACCAGAAAGTCACGGCTAACTACGTGCCAGCAGCCGC<br>GGTAATACGTAGGTGGCAAGCGTTGTCCGGATTTATTGGGCGTAAAGCGAGCGCAGGCGGAAAAATAAGTCTAATGT<br>GAAAGCCCTCGGCTTAACCGAGGAACTGCATCGGAAACTGTTTTTCTTGAGTGCAGAAGAGGAGAGTGGAACTCCAT<br>GTGTAGCGGTGGAATGCGTAGATATATGGAAGAACACCAGTGGCGAAGGCGGCTCTCTGGTCTGCAACTGACGCTGA<br>GGCTCGAAAGCATGGGTAGCGAACAGGATTAGATACCCTGGTAGTCCATGCCGTAAACGATGAGTGCTAAGTGTTGG<br>GAGGTTTCCGCCTCTCAGTGCTGCAGCTAACGCATTAAGCACTCCGCCTGGGGAGTACGACCGCAAGGTTGAAACTCA<br>AAGGAATTGACGGGGGCCCGCACAAGCGGTGGAGCATGTGGTTTAATTCGAAGCAACGCGAAGAACCTTACCAGGTC<br>TTGACATCTAGTGCCATCTTCAGAGATGAAGAGTTCCCTTCGGGGACGCTAAGACAGGTGGTGCATGGCTGTCGTCAG<br>CTCGTGTCGTGAGATGTTGGGTTAAGTCCCGCAACGAGCGCAACCCTTGTTATTAGTTGCCAGCATTAAGTTGGGCACT<br>CTAATGAGACTGCCGGTGACAAACCGGAGGAAGGTGGGGATGACGTCAAGTCATCATGCCCCTTATGACCTGGGCTA<br>CACACGTGCTACAATGGGCAGTACAACGAGAAGCGAGCCTGCGAAGGCAAGCGAATCTCTGAAAGCTGTTCTCAGTT<br>CGGACTGCAGTCTGCAACTCGACTGCACGAAGCTGGAATCGCTAGTAATCGCGGATCAGCACGCCGCGGTGAATACG<br>TTCCCGAGCCTTGTACACACCGCCCGTCACACCATGGGAGTCTGCAACGCCCAAAGCCGGTGGCCTAACCGAAAGGA<br>AGGAGCCGTCTAAGGCAGGGCAGATGACTGGGGTGAAGTCGTAACAAGGTAGCCGTAGGAGAACCTGCGGCTGGATC<br>ACCTC
	Kx146C1	GTTTGATCCTGGCTCAGGACGAACGCTGGCGGCGTGCCTAATACATGCAAGTCGAGCGAGCGGAACCAGCAGATCTG<br>CTTCGGCAGTGACGCTGGGAAAGCGAGCGGCGGATGGGTGAGTAACACGTGGGGAACCTGCCCCAAAGTCTGGGATA<br>CCACTTGGAAACAGGTGCTAATACCGGATAAGAAAGCAGATCGCATGATCAGCTTTTAAAAGGCGGCGTAAGCTGTC<br>GCTATGGGATGGCCCCGCGGTGCATTAGCTAGTTGGTAGAGTAACGGCCTACCAAGGCAATGATGCATAGCCGAGTTG<br>AGAGACTGATCGGCCACATTGGGACTGAGACACGGCCCAAACTCCTACGGGAGGCAGCAGTAGGGAATCTTCCACAA<br>TGGACGCAAGTCTGATGGAGCAACGCCGCGTGAGTGAAGAAGGTTTTCGGATCGTAAAGCTCTGTTGTTGGTGAAGA<br>AGGATAGAGGTAGTAACTGGCCTTTATTTGACGGTAATCAACCAGAAAGTCACGGCTAACTACGTGCCAGCAGCCGC<br>GGTAATACGTAGGTGGCAAGCGTTGTCCGGATTTATTGGGCGTAAAGCGAGCGCAGGCGGAAAAATAAGTCTAATGT<br>GAAAGCCCTCGGCTTAACCGAGGAACTGCATCGGAAACTGTTTTTCTTGAGTGCAGAAGAGGAGAGTGGAACTCCAT<br>GTGTAGCGGTGGAATGCGTAGATATATGGAAGAACACCAGTGGCGAAGGCGGCTCTCTGGTCTGCAACTGACGCTGA<br>GGCTCGAAAGCATGGGTAGCGAACAGGATTAGATACCCTGGTAGTCCATGCCGTAAACGATGAGTGCTAAGTGTTGG<br>GAGGTTTCCGCCTCTCAGTGCTGCAGCTAACGCATTAAGCACTCCGCCTGGGGAGTACGACCGCAAGGTTGAAACTCA<br>AAGGAATTGACGGGGGCCCGCACAAGCGGTGGAGCATGTGGTTTAATTCGAAGCAACGCGAAGAACCTTACCAGGTC<br>TTGACATCTAGTGCCATCTTCAGAGATGAAGAGTTCCCTTCGGGGACGCTAAGACAGGTGGTGCATGGCTGTCGTCAG<br>CTCGTGTCGTGAGATGTTGGGTTAAGTCCCGCAACGAGCGCAACCCTTGTTATTAGTTGCCAGCATTAAGTTGGGCACT<br>CTAATGAGACTGCCGGTGACAAACCGGAGGAAGGTGGGGATGACGTCAAGTCATCATGCCCCTTATGACCTGGGCTA

		CACACGTGCTACAATGGGCAGTACAACGAGAAGCGAGCCTGCGAAGGCAAGCGAATCTCTGAAAGCTGTTCTCAGTT<br>CGGACTGCAGTCTGCAACTCGACTGCACGAAGCTGGAATCGCTAGTAATCGCGGATCAGCACGCCGCGGTGAATACG<br>TTCCCGAGCCTTGTACACACCGCCCGTCACACCATGGGAGTCTGCAACGCCCAAAGCCGGTGGCCTAACCGAAAGGA<br>AGGAGCCGTCTAAGGCAGGGCAGATGACTGGGGTGAAGTCGTAACAAGGTAGCCGTAGGAGAACCTGCGGCTGGATC<br>ACCTC
	TMPC 4D12A	GGCGTGCGGGAGCTATACATGCAGTCGAGCGAGCGGAACCAGCAGAATCACTTCGGTGAGGACGCTGGGAAAGCGA<br>GCGGCGGATGGGTGAGTAACACGTGGGGAACCTGCCCTTAAGTCTGGGATACCACTTGGAAACAGGTGCTAATACCG<br>GATAAGAAAGCAGTTCGCATGAACAGCTTTTAAAAGGCGGCGCAAGCTGTCGCTAAAGGATGGACCCGCGGTGCATT<br>AGCTAGTTGGTAAGGTAACGGCCTACCAAGGCAGTGATGCATAGCCGAGTTGAGAGACTGATCGGCCACATTGGGAC<br>TGAGACACGGCCCAAACTCCTACGGGAGGCAGCAGTAGGGAATCTTCCACAATGGACGCAAGTCTGATGGAGCAACG<br>CCGCGTGAGTGAAGAAGGTTTTCGGACCGTAAAGCTCTGTTGTTGGTGAAGAAGGATAGAGGTAGTAACTGGCCTTTA<br>TTTGACGGTAATCAACCAGAAAGTCACGGCTAACTACGTGCCAGCAGCCGCGGTAATACGTAGGTGGCAAGCGTTGTC<br>CGGATTTATTGGGCGTAAAGCGAGCGCAGGCGGAAGAATAAGTCTGATGTGAAAGCCCTCGGCTTAACCGAGGAATT<br>GCATCGGAAACTGTTTTTCTTGAGTGCAGAAGAGGAGAGTAGAACTCCATGTGTAGCGGTGGAATGCGTAGATATATG<br>GAAGAATACCAGTGGCGAAGGCGGCTCTCTGGTCTGCAACTGACGCTGAGGCTCGAAAGCATGGGTAGCGAACAGGA<br>TTAGATACCCTGGTAGTCCATGCCGTAAACGATGAGTGCTAAGTGTTGGGAGGCTTCCGCCTCTCAGTGCTGCAGCTA<br>ACGCATTAAGCACTCCGCCTGGGGAGTACGACCGCAAGGTTGAAACTCAAAGGAATTGACGGGGGCCCGCACAAGCG<br>GTGGAGCATGTGGTTTAATTCGAAGCAACGCGAAGAACCTTACCAGGTCTTGACATCTAGTGCCATTTGTAGAGATAC<br>AAAGTTCCCTTCGGGGACGCTAAGAACAGGTGGTGCATGCTGTCGTCAGCTCGTGTCGTGAGATGTTGGGTAAGTCCG<br>CACGAGCGCAACCCTTGTTATTAGTTGCCAGCATAGTGACACTTCTATGAGACTGCCGGTGACAACGAGGAGGTGGGA<br>TGACGTCAGTCATCATGCCTTATGACTGGCTCACACACGTGCTACATGGCCAGCACGAGGCACGAGACCTGCAAAGTC<br>AAGCAATCTCTTAGAGGCGTGTTCTCTCACAGA
	TMPC 46M11	CGGCATCGGCGCTATAATGCAGTCGAGCGAGCAGAACCAGCAGATTTACTTCGGTAATGACGCTGGGGACGCGAGCG<br>GCGGATGGGTGATTAACACGTGGGGAACCTGCCCCATAGTCTAGGATACCACTTGGAAACAGGTGCTAATACCGGAT<br>AATAAAGCAGATCGCATGATCAGCTTATAAAAGGCGGCGTAAGCTGTCGCTATGGGATGGCCCCGCGGTGCATTAGCT<br>AGTTGGTAAGGTAACGGCTTACCAAGGCAATGATGCATAGCCGAGTTGAGAGACTGAACGGCCACATTGGGACTGAG<br>ACACGGCCCAAACTCCTACGGGAGGCAGCAGTAGGGAATCTTCCACAATGGACGCAAGTCTGATGGAGCAACGCCGC<br>GTGAGTGAAGAAGGTTTTCGGATCGTAAAGCTCTGTTGTTGGTGAAGAAGGATAGAGGTAGTAACTGGCCTTTATTTG<br>ACGGTAATCAACCAGAAAGTCACGGCTAACTACGTGCCAGCAGCCGCGGTAATACGTAGGTGGCAAGCGTTGTCCGG<br>ATTTATTGGGCGTAAAGCGAGCGCAGGCGGAAGAATAAGTCTGATGTGAAAGCCCTCGGCTTAACCGAGGAATTGCA<br>TCGGAAACTGTTTTTCTTGAGTGCAGAAGAGGAGAGTGGAACTCCATGTGTAGCGGTGGAATGCGTAGATATATGGAA<br>GAACACCAGTGGCGAAGGCGGCTCTCTGGTCTGCAACTGACGCTGAGGCTCGAAAGCATGGGTAGCGAACAGGATTA<br>GATACCCTGGTAGTCCATGCCGTAAACGATGAGTGCTAAGTGTTGGGAGGTTTCCGCCTCTCAGTGCTGCAGCTAACG<br>CATTAAGCACTCCGCCTGGGGAGTACGACCGCAAGGGTTGAAACTCAAAGGAATTGACGGGGGCCCGCACAAGCGGT

		GGAGCATGTGGGTTTAATTCGAAGCAACGCGAAGAACCTTACCAGGTCTTGACATCTAGTGCATCCTAAGAGATTAGGAGTTCCCTTTCGGGGACGCTAAGACAGGTGTGCATGACTGTCGTCAGCTCGTGTCGTGAGATGTGGGTAGTCCGCACGAGCGCACCTATATAGTGCAGCATAGGTGGCACTCTATGAGACTGACGATGAACAACCGGAGG
	TMPC4D171	GGCAGGCACGGCTATACATGCAAGTCGAGCGAGTGGAACCAGCAGAATCACTTCGGTGAGGACGCTGGGAAAGCGAGCGGCGGATGGGTGAGTAACACGTGGGGAACCTGCCCTTAAGTCTGGGATACCACTTGGAAACAGGTGCTAATACCGGATAAGAAAGCAGTTCGCATGAACAGCTTTTAAAAGGCGGCGCAAGCTGTCGCTAAAGGATGGACCCGCGGTGCATTAGCTAGTTGGTAAGGTAACGGCCTACCAAGGCAGTGATGCATAGCCGAGTTGAGAGACTGATCGGCCACATTGGGACTGAGACACGGCCCAAACTCCTACGGGAGGCAGCAGTAGGGAATCTTCCACAATGGACGCAAGTCTGATGGAGCAACGCCGCGTGAGTGAAGAAGGTTTTCGGACCGTAAAGCTCTGTTGTTGGTGAAGAAGGATAGAGGTAGTAACTGGCCTTTATTTGACGGTAATCAACCAGAAAGTCACGGCTAACTACGTGCCAGCAGCCGCGGTAATACGTAGGTGGCAAGCGTTGTCCGGATTTATTGGGCGTAAAGCGAGCGCAGGCGGAAGAATAAGTCTGATGTGAAAGCCCTCGGCTTAACCGAGGAATTGCATCGGAAACTGTTTTTCTTGAGTGCAGAAGAGGAGAGTAGAACTCCATGTGTAGCGGTGGAATGCGTAGATATATGGAAGAATACCAGTGGCGAAGGCGGCTCTCTGGTCTGCAACTGACGCTGAGGCTCGAAAGCATGGGTAGCGAACAGGATTAGATACCCTGGTAGTCCATGCCGTAAACGATGAGTGCTAAGTGTTGGGAGGCTTCCGCCTCTCAGTGCTGCAGCTAACGCATTAAGCACTCCGCCTGGGGAGTACGACCGCAAGGTTGAAACTCAAGGAATTGACGGGGGCCCGCACAAGCGGTGGAGCATGTGGTTTAATTCGAGCACGCGAGATCTTACCAGGTCTTGACATCTAGTGCATTTGTAGAGATACAAGTTCCTTCGGGACGCTAGACAGTGATGCATGCTGTCGTCAGCTCGTGTCGTGAGATGTTGGGTAGTCCCGCACGAGCGCAACCTTGTTATTAGTTG
GS01	JCP8108	GGATCTGACCAGCTTGCTGGTTGGTGAGAGTGGCGAACGGGTGAGTAATGCGTGACCAACCTGCCCCATGCTCCAGAATAGCTCTTGGAAACGGGTGGTAATGCTGGATGCTCCAACTTGACGCATGTCTTGTTGGGAAAGTGTTTAGTGGCATGGGATGGGGTCGCGTCCTATCAGCTTGTAGGCGGGGTAATGGCCCACCTAGGCTTCGACGGGTAGCCGGCCTGAGAGGGCGGACGGCCACATTGGGACTGAGATACGGCCCAGACTCCTACGGGAGGCAGCAGTGGGGAATATTGCGCAATGGGGGGAACCCTGACGCAGCGACGCCGCGTGCGGGATGAAGGCCTTCGGGTTGTAAACCGCTTTTGATTGGGAGCAAGCGGGTGAGTGTACCTTTCGAATAAGCGCCGGCTAACTACGTGCCAGCAGCCGCGGTAATACGTAGGGCGCAAGCGTTATCCGGAATTATTGGGCGTAAAGAGCTTGTAGGCGGTTCGTCGCGTCTGGTGTGAAAGCCCATCGCTTAACGGTGGGTTTGCGCCGGGTACGGGCGGGCTAGAGTGCAGTAGGGGAGACTGGAATTCTCGGTGTAACGGTGGAATGTGTAGATATCGGGAAGAACACCAATGGCGAAGGCAGGTCTCTGGGCTGTTACTGACGCTGAGAAGCGAAAGCGTGGGGAGCGAACAGGATTAGATACCCTGGTAGTCCACGCCGTAAACGGTGGACGCTGGATGTGGGGCCCATTCCACGGGTTCTGTGTCGGAGCTAACGCGTTAAGCGTCCCGCCTGGGGAGTACGGCCGCAAGGCTAAAACTCAAAGAAATTGACGGGGGCCCGCACAAGCGGCGGAGCATGCGGATTAATTCGATGCAACGCGAAGAACCTTACCTGGGCTTGACATGTGCCTGACGACTGCAGAGATGTGGTTTCCTTTCGGGGCAGGTTCACAGGTGGTGCATGGTCGTCGTCAGCTCGTGTCGTGAGATGTTGGGTTAAGTCCCGCAACGAGCGCAACCCTCGCCCTGTGTTGCCAGCGGGTTATGCCGGGAACTCACGGGGGACCGCCGGGGTTAACTCGGAGGAAGGTGGGGATGACGTCAGATCATCATGCCCCTTACGTCCAGGGCTTCACGCATGCTACAATGGCCAGTACAACGGGTTGCTTCATGGTGACATGGTGCTAATCCCTTAAAACTGGTCTCAGTTCGGATCGTAGTCTGCAACTCGACTACGTGAA

		GGCGGAGTCGCTAGTAATCGCGAATCAGCAACGTCGCGGTGAATGCGTTCCCGGGCCTTGTACACACCGCCCGTCAAG<br>TCATGAAAGTGGGCAGCACCCGAAGCCGGTGGCCTAACCCTTTTGGGATGGAGCCGTCTAAGGTGAGGCT
	UGent25.49	TGGAGGGTTCGATTCTGGCTCAGGATGAACGCTGGCGGCGTGCTTAACACATGCAAGTCGAACGGGATCTGACCAGCT<br>TGCTGGTTGGTGAGAGTGGCGAACGGGTGAGTAATGCGTGACCAACCTGCCCCATGCTCCAGAATAGCTCTTGGAAAC<br>GGGTGGTAATGCTGGATGCTCCAACTTGACGCATGTCTTGTTGGGAAAGTGTTTAGTGGCATGGGATGGGGTCGCGTC<br>CTATCAGCTTGTAGGCGGGGTAATGGCCCACCTAGGCTTCGACGGGTAGCCGGCCTGAGAGGGCGGACGGCCACATT<br>GGGACTGAGATACGGCCCAGACTCCTACGGGAGGCAGCAGTGGGGAATATTGCGCAATGGGGGAAACCCTGACGCAG<br>CGACGCCGCGTGCGGGATGAAGGCCTTCGGGTTGTAAACCGCTTTTGATTGGGAGCAAGCCTTTTGGGTGAGTGTACC<br>TTTCGAATAAGCGCCGGCTAACTACGTGCCAGCAGCCGCGGTAATACGTAGGGCGCAAGCGTTATCCGGAATTATTGG<br>GCGTAAAGAGCTTGTAGGCGGTTCGTCGCGTCTGGTGTGAAAGCCCATCGCTTAACGGTGGGTTTGCGCCGGGTACGG<br>GCGGGCTAGAGTGCAGTAGGGGAGACTGGAATTCTCGGTGTAACGGTGGAATGTGTAGATATCGGGAAGAACACCAA<br>TGGCGAAGGCAGGTCTCTGGGCTGTTACTGACGCTGAGAAGCGAAAGCGTGGGGAGCGAACAGGATTAGATACCCTG<br>GTAGTCCACGCCGTAAACGGTGGACGCTGGATGTGGGGCCCATTCCACGGGTTCTGTGTCGGAGCTAACGCGTTAAGC<br>GTCCCGCCTGGGGAGTACGGCCGCAAGGCTAAAACTCAAAGAAATTGACGGGGGCCCGCACAAGCGGCGGAGCATGC<br>GGATTAATTCGATGCAACGCGAAGAACCTTACCTGGGCTTGACATGTGCCTGACGACTGCAGAGATGTGGTTTCCTTT<br>CGGGGCAGGTTCACAGGTGGTGCATGGTCGTCGTCAGCTCGTGTCGTGAGATGTTGGGTTAAGTCCCGCAACGAGCGC<br>AACCCTCGCCCTGTGTTGCCAGCGGGTTATGCCGGGAACTCACGGGGGACCGCCGGGGTTAACTCGGAGGAAGGTGG<br>GGATGACGTCAGATCATCATGCCCCTTACGTCCAGGGCTTCACGCATGCTACAATGGCCAGTACAACGGGTTGCTTCA<br>TGGTGACATGGTGCTAATCCCTTAAAACTGGTCTCAGTTCGGATCGTAGTCTGCAACTCGACTACGTGAAGGCGGAGT<br>CGCTAGTAATCGCGAATCAGCAACGTCGCGGTGAATGCGTTCCCGGGCCTTGTACACACCGCCCGTCAAGTCATGAAA<br>GTGGGCAGCACCCGAAGCCGGTGGCCTAACCCTTTTGGGATGGAGCCGTCTAAGGTGAGGCTCGTGATTGGGACTAA<br>GTCGTAACAAGGTAGCCGTACCGGAAGGTGCGGCTGGATCACCTCCTTT
	UGent09.01	TGGAGGGTTCGATTCTGGCTCAGGATGAACGCTGGCGGCGTGCTTAACACATGCAAGTCGAACGGGATCTGACCAGCT<br>TGCTGGTTGGTGAGAGTGGCGAACGGGTGAGTAATGCGTGACCAACCTGCCCCATGCTCCAGAATAGCTCTTGGAAAC<br>GGGTGGTAATGCTGGATGCTCCAACTTGACGCATGTCTTGTTGGGAAAGTGTTTAGTGGCATGGGATGGGGTCGCGTC<br>CTATCAGCTTGTAGGCGGGGTAATGGCCCACCTAGGCTTCGACGGGTAGCCGGCCTGAGAGGGCGGACGGCCACATT<br>GGGACTGAGATACGGCCCAGACTCCTACGGGAGGCAGCAGTGGGGAATATTGCGCAATGGGGGAAACCCTGACGCAG<br>CGACGCCGCGTGCGGGATGAAGGCCTTCGGGTTGTAAACCGCTTTTGATTGGGAGCAAGCCTTTTGGGTGAGTGTACC<br>TTTCGAATAAGCGCCGGCTAACTACGTGCCAGCAGCCGCGGTAATACGTAGGGCGCAAGCGTTATCCGGAATTATTGG<br>GCGTAAAGAGCTTGTAGGCGGTTCGTCGCGTCTGGTGTGAAAGCCCATCGCTTAACGGTGGGTTTGCGCCGGGTACGG<br>GCGGGCTAGAGTGCAGTAGGGGAGACTGGAATTCTCGGTGTAACGGTGGAATGTGTAGATATCGGGAAGAACACCAA<br>TGGCGAAGGCAGGTCTCTGGGCTGTTACTGACGCTGAGAAGCGAAAGCGTGGGGAGCGAACAGGATTAGATACCCTG<br>GTAGTCCACGCCGTAAACGGTGGACGCTGGATGTGGGGCCCATTCCACGGGTTCTGTGTCGGAGCTAACGCGTTAAGC<br>GTCCCGCCTGGGGAGTACGGCCGCAAGGCTAAAACTCAAAGAAATTGACGGGGGCCCGCACAAGCGGCGGAGCATGC

		GGATTAATTCGATGCAACGCGAAGAACCTTACCTGGGCTTGACATGTGCCTGACGACTGCAGAGATGTGGTTTCCTTT<br>CGGGGCAGGTTCACAGGTGGTGCATGGTCGTCGTCAGCTCGTGTCGTGAGATGTTGGGTTAAGTCCCGCAACGAGCGC<br>AACCCTCGCCCTGTGTTGCCAGCGGGTTATGCCGGGAACTCACGGGGGACCGCCGGGGTTAACTCGGAGGAAGGTGG<br>GGATGACGTCAGATCATCATGCCCCTTACGTCCAGGGCTTCACGCATGCTACAATGGCCAGTACAACGGGTTGCTTCA<br>TGGTGACATGGTGCTAATCCCTTAAAACTGGTCTCAGTTCGGATCGTAGTCTGCAACTCGACTACGTGAAGGCGGAGT<br>CGCTAGTAATCGCGAATCAGCAACGTCGCGGTGAATGCGTTCCCGGGCCTTGTACACACCGCCCGTCAAGTCATGAAA<br>GTGGGCAGCACCCGAAGCCGGTGGCCTAACCCTTTTGGGATGGAGCCGTCTAAGGTGAGGCTCGTGATTGGGACTAA<br>GTCGTAACAAGGTAGCCGTACCGGAAGGTGCGGCTGGATCACCTCCTTT
	UGent09.07	TGGAGGGTTCGATTCTGGCTCAGGATGAACGCTGGCGGCGTGCTTAACACATGCAAGTCGAACGGGATCTGACCAGCT<br>TGCTGGTTGGTGAGAGTGGCGAACGGGTGAGTAATGCGTGACCAACCTGCCCCATGCTCCAGAATAGCTCTTGGAAAC<br>GGGTGGTAATGCTGGATGCTCCAACTTGACGCATGTCTTGTTGGGAAAGTGTTTAGTGGCATGGGATGGGGTCGCGTC<br>CTATCAGCTTGTAGGCGGGGTAATGGCCCACCTAGGCTTCGACGGGTAGCCGGCCTGAGAGGGCGGACGGCCACATT<br>GGGACTGAGATACGGCCCAGACTCCTACGGGAGGCAGCAGTGGGGAATATTGCGCAATGGGGGAAACCCTGACGCAG<br>CGACGCCGCGTGCGGGATGAAGGCCTTCGGGTTGTAAACCGCTTTTGATTGGGAGCAAGCCTTTTGGGTGAGTGTACC<br>TTTCGAATAAGCGCCGGCTAACTACGTGCCAGCAGCCGCGGTAATACGTAGGGCGCAAGCGTTATCCGGAATTATTGG<br>GCGTAAAGAGCTTGTAGGCGGTTCGTCGCGTCTGGTGTGAAAGCCCATCGCTTAACGGTGGGTTTGCGCCGGGTACGG<br>GCGGGCTAGAGTGCAGTAGGGGAGACTGGAATTCTCGGTGTAACGGTGGAATGTGTAGATATCGGGAAGAACACCAA<br>TGGCGAAGGCAGGTCTCTGGGCTGTTACTGACGCTGAGAAGCGAAAGCGTGGGGAGCGAACAGGATTAGATACCCTG<br>GTAGTCCACGCCGTAAACGGTGGACGCTGGATGTGGGGCCCATTCCACGGGTTCTGTGTCGGAGCTAACGCGTTAAGC<br>GTCCCGCCTGGGGAGTACGGCCGCAAGGCTAAAACTCAAAGAAATTGACGGGGGCCCGCACAAGCGGCGGAGCATGC<br>GGATTAATTCGATGCAACGCGAAGAACCTTACCTGGGCTTGACATGTGCCTGACGACTGCAGAGATGTGGTTTCCTTT<br>CGGGGCAGGTTCACAGGTGGTGCATGGTCGTCGTCAGCTCGTGTCGTGAGATGTTGGGTTAAGTCCCGCAACGAGCGC<br>AACCCTCGCCCTGTGTTGCCAGCGGGTTATGCCGGGAACTCACGGGGGACCGCCGGGGTTAACTCGGAGGAAGGTGG<br>GGATGACGTCAGATCATCATGCCCCTTACGTCCAGGGCTTCACGCATGCTACAATGGCCAGTACAACGGGTTGCTTCA<br>TGGTGACATGGTGCTAATCCCTTAAAACTGGTCTCAGTTCGGATCGTAGTCTGCAACTCGACTACGTGAAGGCGGAGT<br>CGCTAGTAATCGCGAATCAGCAACGTCGCGGTGAATGCGTTCCCGGGCCTTGTACACACCGCCCGTCAAGTCATGAAA<br>GTGGGCAGCACCCGAAGCCGGTGGCCTAACCCTTTTGGGATGGAGCCGTCTAAGGTGAGGCTCGTGATTGGGACTAA<br>GTCGTAACAAGGTAGCCGTACCGGAAGGTGCGGCTGGATCACCTCCTTT
	ATCC14018<br>_2	TTTCGTGGAGGGTTCGATTCTGGCTCAGGATGAACGCTGGCGGCGTGCTTAACACATGCNAGTCGAACGGGATCTGAC<br>CAGCTTGCTGGTTGGTGAGAGTGGCGAACGGGTGAGTAATGCGTGACCAACCTGCCCCATGCTCCAGAATAGCTCTTG<br>GAAACGGGTGGTAATGCTGGATGCTCCAACTTGACGCATGTCTTGTTGGGAAAGTGTTTAGTGGCATGGGATGGGGTC<br>GCGTCCTATCAGCTTGTAGGCGGGGTAATGGCCCACCTAGGCTTCGACGGGTAGCCGGCCTGAGAGGGCGGACGGCC<br>ACATTGGGACTGAGATACGGCCCAGACTNCTACGGGAGGCAGCAGTGGGGAATATTGCGCAATGGGGGAAACCCTGA<br>CGCAGCGACGNCGCGTGCGGGATGAAGGCCTTCGGGTTGTAAACCGCTTTTGATTGGGAGCAAGCCTTTTGGGTGAGT

		GTACCTTTCGAATAAGCGCCGGCTAACTACGTGCCAGCAGCCGCGGTAATACGTAGGGCGCAAGCGTTATCCGGAATTATTGGGCGTAAAGAGCTTGTAGGCGGTTCGTCGCGTCTGGTGTGAAAGCCCATCGCTTAACGGTGGGNTTGCGCCGGGTACGGGCGGGCTAGAGTGCAGTAGGGGAGACTGGAATTCTCGGTGTAACGGTGGAATGTGTAGATATCGGGAAGAACACCAATGGCGAAGGCAGGTCTCTGGGCTGTTACTGACGCTGAGAAGCGAAAGCGTGGGGAGCGAACAGGATTAGATACCCTGGTAGTCCACGCCGTAAACGGTGGACGCTGGATGTGGGGCCCATTCCACGGGTTCTGTGTCGGAGCTAACGCGTTAAGCGTCCCGCCTGGGGAGTACGGCCGCAAGGCTAAAACTCAAAGAAATTGACGGGGGCCNGCACAAGCGGCGGAGCATGCGGATTAATTCGATGNAACGCGAAGAACCTTACCTGGGCTTGACATGTGCCTGACGACTGCAGAGATGTGGTTTCCNTTCGGGGCAGGTTCACAGGTGGTGCATGGTCGTCGTCAGCTCGTGTCGTGAGATGTTGGGTTAAGTCCCGCAACGAGCGCAACCCTCGCCCTGTGTTGCCAGCGGGTTATGCCGGGAACTCACGGGGGACCGCCGGGGTTACCNCGGAGGAAGGTGGGNATGACGTCAGATCATCATGCCCCTTACGTCCAGGGCTTCACGCATGCTACAATGGCCAGTACAACGGGTTGCTTCATGGTGACATGGTGCTAATCCCTTAAAACTNGTCTCAGTTCGGATCGTAGTCTGCAACTCGACTACGTGAAGGCGGAGTCGCTAGTAATCGCGAATCAGCAACGTCGCGGTGAATGCGTTCCCGGGCCTTGTACACACCGCCCGTCAAGTCATGAAAGTGGGCAGCACCCGAAGCCGGTGGCCTAACCCTTTTGGGATGGAGCCGTCTAAGGTGAGGCTCGTGATTGGG
GS02	UGent21.28	TGGAGGGTTCGATTCTGGCTCAGGATGAACGCTGGCGGCGTGCTTAACACATGCAAGTCGAACGGGATCCGACCAGCTTGCTGGTTGGTGAGAGTGGCGAACGGGTGAGTAATGCGTGACCAACCTGCCCCATGCTCCAGAATAGCTCTTGGAAACGGGTGGTAATGCTGGATGCTCCAACTTGACGCATGTCTTGTTGGGAAAGTGTTTAGTGGCATGGGATGGGGTCGCGTCCTATCAGCTTGTAGGCGGGGTAATGGCCCACCTAGGCTTCGACGGGTAGCCGGCCTGAGAGGGCGGACGGCCACATTGGGACTGAGATACGGCCCAGACTCCTACGGGAGGCAGCAGTGGGGAATATTGCGCAATGGGGGAAACCCTGACGCAGCGACGCCGCGTGCGGGATGAAGGCCTTCGGGTTGTAAACCGCTTTTGATTGGGAGCAAGCTTTCGGGTGAGTGTACCTTTCGAATAAGCGCCGGCTAACTACGTGCCAGCAGCCGCGGTAATACGTAGGGCGCAAGCGTTATCCGGAATTATTGGGCGTAAAGAGCTTGTAGGCGGTTCGTCGCGTCTGGTGTGAAAGCCCATCGCTTAACGGTGGGTTTGCGCCGGGTACGGGCGGGCTAGAGTGCAGTAGGGGAGACTGGAATTCCCGGTGTAACGGTGGAATGTGTAGATATCGGGAAGAACACCAATGGCGAAGGCAGGTCTCTGGGCTGTTACTGACGCTGAGAAGCGAAAGCGTGGGGAGCGAACAGGATTAGATACCCTGGTAGTCCACGCCGTAAACGGTGGACGCTGGATGTGGGGCCCATTCCACGGGTTCTGTGTCGGAGCTAACGCGTTAAGCGTCCCGCCTGGGGAGTACGGCCGCAAGGCTAAAACTCAAAGAAATTGACGGGGGCCCGCACAAGCGGCGGAGCATGCGGATTAATTCGATGCAACGCGAAGAACCTTACCTGGGCTTGACATGTGCCTGTCGACTGCAGAGATGTGGTTTCCCTTCGGGGCAGGTTCACAGGTGGTGCATGGTCGTCGTCAGCTCGTGTCGTGAGATGTTGGGTTAAGTCCCGCAACGAGCGCAACCCTCGCCCTGTGTTGCCAGCGGGTTATGCCGGGAACTCACGGGGGACCGCCGGGGTTAACTCGGAGGAAGGTGGGGATGACGTCAGATCATCATGCCCCTTACGTCCAGGGCTTCACGCATGCTACAATGGCCGGTACAACGGGATGCGACATGGTGACATGGAGCGGATCCCTTAAAACCGGTCTCAGTTCGGATCGTAGTCTGCAACTCGACTACGTGAAGGCGGAGTCGCTAGTAATCGCGAATCAGCAACGTCGCGGTGAATGCGTTCCCGGGCCTTGTACACACCGCCCGTCAAGTCATGAAAGTGGGCAGCACCCGAAGCCGGTGGCCTAACCCTTTTGGGATGGAGCCGTCTAAGGTGAGGCTCGTGATTGGGACTAAGTCGTAACAAGGTAGCCGTACCGGAAGGTGCGGCTGGATCACCTCCTTT

	UGent1801	GTGAGTGTACCTTTCGAATAAGCGCCGGCTAACTACGTGCCAGCAGCCGCGGTAATACGTAGGGCGCAAGCGTTATCC<br>GGAATTATTGGGCGTAAAGAGCTTGTAGGCGGTTCGTCGCGTCTGGTGTGAAAGCCCATCGCTTAACGGTGGGTTTGC<br>GCCGGGTACGGGCGGGCTAGAGTGCAGTAGGGGAGACTGGAATTCCCGGTGTAACGGTGGAATGTGTAGATATCGGG<br>AAGAACACCAATGGCGAAGGCAGGTCTCTGGGCTGTTACTGACGCTGAGAAGCGAAAGCGTGGGGAGCGAACAGGA<br>TTAGATACCCTGGTAGTCCACGCCGTAAACGGTGGACGCTGGATGTGGGGCCCATTCCACGGGTTCTGTGTCGGAGCT<br>AACGCGTTAAGCGTCCCGCCTGGGGAGTACGGCCGCAAGGCTAAAACTCAAAGAAATTGACGGGGGCCCGCACAAGC<br>GGCGGAGCATGCGGATTAATTCGATGCAACGCGAAGAACCTTACCTGGGCTTGACATGTGCCTGTCGACTGCAGAGAT<br>GTGGTTTCCCTTCGGGGCAGGTTCACAGGTGGTGCATGGTCGTCGTCAGCTCGTGTCGTGAGATGTTGGGTTAAGTCCC<br>GCAACGAGCGCAACCCTCGCCCTGTGTTGCCAGCGGGTTATGCCGGGAACTCACGGGGGACCGCCGGGGTTAACTCG<br>GAGGAAGGTGGGGATGACGTCAGATCATCATGCCCCTTACGTCCAGGGCTTCACGCATGCTACAATGGCCGGTACAAC<br>GGGATGCGACATGGTGACATGGAGCGGATCCCTTAAAACCGGTCTCAGTTCGGATCGTAGTCTGCAACTCGACTACGT<br>GAAGGCGGAGTCGCTAGTAATCGCGAATCAGCAACGTCGCGGTGAATGCGTTCCCGGGCCTTGTACACACCGCCCGTC<br>AAGTCATGAAAGTGGGCAGCACCCGAAGCCGGTGGCCTAACCCTTTTGGGATGGAGCCGTCTAAGGTGAGGCTCGTG<br>ATTGGGACTAAGTCGTAACAAGGTAGCCGTACCGGAAGGTGCGGCTGGATCACCTCCTTT
GS03	GS9838	TGGAGGGTTCGATTCTGGCTCAGGATGAACGCTGGCGGCGTGCTTAACACATGCAAGTCGAACGGGATCTAGTCAGCT<br>TGCTGACTGGTGAGAGTGGCGAACGGGTGAGTAATGCGTGACCAACCTGCCCCATGCTCCAGAATAGCTCTTGGAAAC<br>GGGTGGTAATGCTGGATGCTCCAACTTGACGCATGTCTTGTTGGGAAAGTGTTTAGTGGCATGGGATGGGGTCGCGTC<br>CTATCAGCTTGTAGGCGGGGTAATGGCCCACCTAGGCTTCGACGGGTAGCCGGCCTGAGAGGGCGGACGGCCACATT<br>GGGACTGAGATACGGCCCAGACTCCTACGGGAGGCAGCAGTGGGGAATATTGCGCAATGGGGGAAACCCTGACGCAG<br>CGACGCCGCGTGCGGGATGAAGGCCTTCGGGTTGTAAACCGCTTTTGATTGGGAGCAAGCCTTCGGGTGAGTGTACCT<br>TTCGAATAAGCGCCGGCTAACTACGTGCCAGCAGCCGCGGTAATACGTAGGGCGCAAGCGTTATCCGGAATTATTGGG<br>CGTAAAGAGCTTGTAGGCGGTTCGTCGCGTCTGGTGTGAAAGCCCATCGCTTAACGGTGGGTCTGCGCCGGGTACGGG<br>CGGGCTAGAGTGCAGTAGGGGAGACTGGAATTCCCGGTGTAACGGTGGAATGTGTAGATATCGGGAAGAACACCAAT<br>GGCGAAGGCAGGTCTCTGGGCTGTTACTGACGCTGAGAAGCGAAAGCGTGGGGAGCGAACAGGATTAGATACCCTGG<br>TAGTCCACGCCGTAAACGGTGGACGCTGGATGTGGGGCCCATTCCACGGGTTCCGTGTCGGAGCTAACGCGTTAAGCG<br>TCCCGCCTGGGGAGTACGGCCGCAAGGCTAAAACTCAAAGAAATTGACGGGGGCCCGCACAAGCGGCGGAGCATGCG<br>GATTAATTCGATGCAACGCGAAGAACCTTACCTGGGCTTGACATGTGCCTGTCGACTGCAGAGATGTGGTTTCCCTTC<br>GGGGCAGGTTCACAGGTGGTGCATGGTCGTCGTCAGCTCGTGTCGTGAGATGTTGGGTTAAGTCCCGCAACGAGCGCA<br>ACCCTCGCCCTGTGTTGCCAGCGGGTTATGCCGGGAACTCACGGGGGACCGCCGGGGTTAACTCGGAGGAAGGTGGG<br>GATGACGTCAGATCATCATGCCCCTTACGTCCAGGGCTTCACGCATGCTACAATGGCCGGTACAACGGGATGCGACAT<br>GGTGACATGGAGCGGATCCCTTAAAACCGGTCTCAGTTCGGATCGTAGTCTGCAACTCGACTACGTGAAGGCGGAGTC<br>GCTAGTAATCGCGAATCAGCAACGTCGCGGTGAATGCGTTCCCGGGCCTTGTACACACCGCCCGTCAAGTCATGAAAG<br>TGGGCAGCACCCGAAGCCGGTGGCCTAACCCTTTTGGGATGGAGCCGTCTAAGGTGAGGCTCGTGATTGGGACTAAGT<br>CGTAACAAGGTAGCCGTACCGGAAGGTGCGGCTGGATCACCTCCTTT

	UGent06.41	TGGAGGGTTCGATTCTGGCTCAGGATGAACGCTGGCGGCGTGCTTAACACATGCAAGTCGAACGGGATCTAGTCAGCT<br>TGCTGACTGGTGAGAGTGGCGAACGGGTGAGTAATGCGTGACCAACCTGCCCCATGCTCCAGAATAGCTCTTGGAAAC<br>GGGTGGTAATGCTGGATGCTCCAACTTGACGCATGTCTTGTTGGGAAAGTGTTTAGTGGCATGGGATGGGGTCGCGTC<br>CTATCAGCTTGTAGGCGGGGTAATGGCCCACCTAGGCTTCGACGGGTAGCCGGCCTGAGAGGGCGGACGGCCACATT<br>GGGACTGAGATACGGCCCAGACTCCTACGGGAGGCAGCAGTGGGGAATATTGCGCAATGGGGGAAACCCTGACGCAG<br>CGACGCCGCGTGCGGGATGAAGGCCTTCGGGTTGTAAACCGCTTTTGATTGGGAGCAAGCCTTTTGGGTGAGTGTACC<br>TTTCGAATAAGCGCCGGCTAACTACGTGCCAGCAGCCGCGGTAATACGTAGGGCGCAAGCGTTATCCGGAATTATTGG<br>GCGTAAAGAGCTTGTAGGCGGTTCGTCGCGTCTGGTGTGAAAGCCCATCGCTTAACGGTGGGTCTGCGCCGGGTACGG<br>GCGGGCTAGAGTGCAGTAGGGGAGACTGGAATTCCCGGTGTAACGGTGGAATGTGTAGATATCGGGAAGAACACCAA<br>TGGCGAAGGCAGGTCTCTGGGCTGTTACTGACGCTGAGAAGCGAAAGCGTGGGGAGCGAACAGGATTAGATACCCTG<br>GTAGTCCACGCCGTAAACGGTGGACGCTGGATGTGGGGCCCATTCCACGGGTTCCGTGTCGGAGCTAACGCGTTAAGC<br>GTCCCGCCTGGGGAGTACGGCCGCAAGGCTAAAACTCAAAGAAATTGACGGGGGCCCGCACAAGCGGCGGAGCATGC<br>GGATTAATTCGATGCAACGCGAAGAACCTTACCTGGGCTTGACATGTGCCTGTCGACTGCAGAGATGTGGTTTCCCTT<br>CGGGGCAGGTTCACAGGTGGTGCATGGTCGTCGTCAGCTCGTGTCGTGAGATGTTGGGTTAAGTCCCGCAACGAGCGC<br>AACCCTCGCCCTGTGTTGCCAGCGGGTTATGCCGGGAACTCACGGGGGACCGCCGGGGTTAACTCGGAGGAAGGTGG<br>GGATGACGTCAGATCATCATGCCCCTTACGTCCAGGGCTTCACGCATGCTACAATGGCCGGTACAACGGGATGCGACA<br>TGGTGACATGGAGCGGATCCCTTAAAACCGGTCTCAGTTCGGATCGTAGTCTGCAACTCGACTACGTGAAGGCGGAGT<br>CGCTAGTAATCGCGAATCAGCAACGTCGCGGTGAATGCGTTCCCGGGCCTTGTACACACCGCCCGTCAAGTCATGAAA<br>GTGGGCAGCACCCGAAGCCGGTGGCCTAACCCTTTTGGGATGGAGCCGTCTAAGGTGAGGCTCGTGATTGGGACTAA<br>GTCGTAACAAGGTAGCCGTACCGGAAGGTGCGGCTGGATCACCTCCTTT
	UGent09.48	TGGAGGGTTCGATTCTGGCTCAGGATGAACGCTGGCGGCGTGCTTAACACATGCAAGTCGAACGGGATCTAGTCAGCT<br>TGCTGACTGGTGAGAGTGGCGAACGGGTGAGTAATGCGTGACCAACCTGCCCCATGCTCCAGAATAGCTCTTGGAAAC<br>GGGTGGTAATGCTGGATGCTCCAACTTGACGCATGTCTTGTTGGGAAAGTGTTTAGTGGCATGGGATGGGGTCGCGTC<br>CTATCAGCTTGTAGGCGGGGTAATGGCCCACCTAGGCTTCGACGGGTAGCCGGCCTGAGAGGGCGGACGGCCACATT<br>GGGACTGAGATACGGCCCAGACTCCTACGGGAGGCAGCAGTGGGGAATATTGCGCAATGGGGGAAACCCTGACGCAG<br>CGACGCCGCGTGCGGGATGAAGGCCTTCGGGTTGTAAACCGCTTTTGATTGGGAGCAAGCCTTCGGGTGAGTGTACCT<br>TTCGAATAAGCGCCGGCTAACTACGTGCCAGCAGCCGCGGTAATACGTAGGGCGCAAGCGTTATCCGGAATTATTGGG<br>CGTAAAGAGCTTGTAGGCGGTTCGTCGCGTCTGGTGTGAAAGCCCATCGCTTAACGGTGGGTCTGCGCCGGGTACGGG<br>CGGGCTAGAGTGCAGTAGGGGAGACTGGAATTCCCGGTGTAACGGTGGAATGTGTAGATATCGGGAAGAACACCAAT<br>GGCGAAGGCAGGTCTCTGGGCTGTTACTGACGCTGAGAAGCGAAAGCGTGGGGAGCGAACAGGATTAGATACCCTGG<br>TAGTCCACGCCGTAAACGGTGGACGCTGGATGTGGGGCCCATTCCACGGGTTCCGTGTCGGAGCTAACGCGTTAAGCG<br>TCCCGCCTGGGGAGTACGGCCGCAAGGCTAAAACTCAAAGAAATTGACGGGGGCCCGCACAAGCGGCGGAGCATGCG<br>GATTAATTCGATGCAACGCGAAGAACCTTACCTGGGCTTGACATGTGCCTGTCGACTGCAGAGATGTGGTTTCCCTTC<br>GGGGCAGGTTCACAGGTGGTGCATGGTCGTCGTCAGCTCGTGTCGTGAGATGTTGGGTTAAGTCCCGCAACGAGCGCA

		ACCCTCGCCCTGTGTTGCCAGCGGGTTATGCCGGGAACTCACGGGGGACCGCCGGGGTTAACTCGGAGGAAGGTGGG<br>GATGACGTCAGATCATCATGCCCCTTACGTCCAGGGCTTCACGCATGCTACAATGGCCGGTACAACGGGATGCGACAT<br>GGTGACATGGAGCGGATCCCTTAAAACCGGTCTCAGTTCGGATCGTAGTCTGCAACTCGACTACGTGAAGGCGGAGTC<br>GCTAGTAATCGCGAATCAGCAACGTCGCGGTGAATGCGTTCCCGGGCCTTGTACACACCGCCCGTCAAGTCATGAAAG<br>TGGGCAGCACCCGAAGCCGGTGGCCTAACCCTTTTGGGATGGAGCCGTCTAAGGTGAGGCTCGTGATTGGGACTAAGT<br>CGTAACAAGGTAGCCGTACCGGAAGGTGCGGCTGGATCACCTCCTTT
GS04	JCP8481	ATGCAAGTCGAACGGGATCCGACCAGCTTGCTGGTTGGTGAGAGTGGCGAACGGGTGAGTAATGCGTGACCAACCTG<br>CCCCATGCTCCAGAATAGCTCTTGGAAACGGGTGGTAATGCTGGATGCTCCAACTTGACGCATGTCTTGTTGGGAAAG<br>TGTTTAGTGGCATGGGATGGGGTCGCGTCCTATCAGCTTGTAGGCGGGGTAATGGCCCACCTAGGCTTCGACGGGTAG<br>CCGGCCTGAGAGGGCGGACGGCCACATTGGGACTGAGATACGGCCCAGACTCCTACGGGAGGCAGCAGTGGGGAATA<br>TTGCGCAATGGGGGAAACCCTGACGCAGCGACGCCGCGTGCGGGATGAAGGCCTTCGGGTTGTAAACCGCTTTTGATT<br>GGGAGCAAGCTTTCGGGTGAGTGTACCTTTCGAATAAGCGCCGGCTAACTACGTGCCAGCAGCCGCGGTAATACGTAG<br>GGCGCAAGCGTTATCCGGAATTATTGGGCGTAAAGAGCTTGTAGGCGGTTCGTCGCGTCTGGTGTGAAAGCCCATCGC<br>TTAACGGTGGGTTTGCGCCGGGTACGGGCGGGCTAGAGTGCAGTAGGGGAGACTGGAATTCCCGGTGTAACGGTGGA<br>ATGTGTAGATATCGGGAAGAACACCAATGGCGAAGGCAGGTCTCTGGGCTGTTACTGACGCTGAGAAGCGAAAGCGT<br>GGGGAGCGAACAGGATTAGATACCCTGGTAGTCCACGCCGTAAACGGTGGACGCTGGATGTGGGGCCCATTCCACGG<br>GTTCTGTGTCGGAGCTAACGCGTTAAGCGTCCCGCCTGGGGAGTACGGCCGCAAGGCTAAAACTCAAAGAAATTGAC<br>GGGGGCCCGCACAAGCGGCGGAGCATGCGGATTAATTCGATGCAACGCGAAGAACCTTACCTGGGCTTGACATGTGC<br>CTGTCGACTGCAGAGATGTGGTTTCCCTTCGGGGCAGGTTCACAGGTGGTGCATGGTCGTCGTCAGCTCGTGTCGTGA<br>GATGTTGGGTTAAGTCCCGCAACGAGCGCAACCCTCGCCCTGTGTTGCCAGCGGGTTATGCCGGGAACTCACGGGGGA<br>CCGCCGGGGTTAACTCGGAGGAAGGTGGGGATGACGTCAGATCATCATGCCCCTTACGTCCAGGGCTTCACGCATGCT<br>ACAATGGCCGGTACAACGGGATGCGACATGGTGACATGGAGCGGATCCCTTAAAACCGGTCTCAGTTCGGATCGTAG<br>TCTGCAACTCGACTACGTGAAGGCGGAGTCGCTAGTAATCGCGAATCAGCAACGTCGCGGTGAATGCGTTCCCGGGCC<br>TTGTACACACCGCCCGTCAAGTCATGAAAGTGGGCAGCACCCGAAGCCGGTGGCCTAACCCTTTTGGGATGGAGCCGT<br>CTAAGGTGAGG
GS05	UMB1686	TGGAGGGTTCGATTCTGGCTCAGGATGAACGCTGGCGGCGTGCTTAACACATGCAAGTCGAACGGGATCTAGTCAGCT<br>TGCTGACTGGTGAGAGTGGCGAACGGGTGAGTAATGCGTGACCAACCTGCCCCATGCTCCAGAATAGCTCTTGGAAAC<br>GGGTGGTAATGCTGGATGCTCCAACTTGACGCATGTCTTGTTGGGAAAGTGTTTAGTGGCATGGGATGGGGTCGCGTC<br>CTATCAGCTTGTAGGCGGGGTAATGGCCCACCTAGGCTTCGACGGGTAGCCGGCCTGAGAGGGCGGACGGCCACATT<br>GGGACTGAGATACGGCCCAGACTCCTACGGGAGGCAGCAGTGGGGAATATTGCGCAATGGGGGAAACCCTGACGCAG<br>CGACGCCGCGTGCGGGATGAAGGCCTTCGGGTTGTAAACCGCTTTTGATTGGGAGCAAGCCTTCGGGTGAGTGTACCT<br>TTCGAATAAGCGCCGGCTAACTACGTGCCAGCAGCCGCGGTAATACGTAGGGCGCAAGCGTTATCCGGAATTATTGGG<br>CGTAAAGAGCTTGTAGGCGGTTCGTCGCGTCTGGTGTGAAAGCCCATCGCTTAACGGTGGGTCTGCGCCGGGTACGGG<br>CGGGCTAGAGTGCAGTAGGGGAGACTGGAATTCCCGGTGTAACGGTGGAATGTGTAGATATCGGGAAGAACACCAAT

		GGCGAAGGCAGGTCTCTGGGCTGTTACTGACGCTGAGAAGCGAAAGCGTGGGGAGCGAACAGGATTAGATACCCTGG<br>TAGTCCACGCCGTAAACGGTGGACGCTGGATGTGGGGCCCATTCCACGGGTTCCGTGTCGGAGCTAACGCGTTAAGCG<br>TCCCGCCTGGGGAGTACGGCCGCAAGGCTAAAACTCAAAGAAATTGACGGGGGCCCGCACAAGCGGCGGAGCATGCG<br>GATTAATTCGATGCAACGCGAAGAACCTTACCTGGGCTTGACATGTGCCTGACAGCTGCAGAGATGTGGTTTCCCTTC<br>GGGGCAGGTTCACAGGTGGTGCATGGTCGTCGTCAGCTCGTGTCGTGAGATGTTGGGTTAAGTCCCGCAACGAGCGCA<br>ACCCTCGCCCTGTGTTGCCAGCGGGTTATGCCGGGAACTCACGGGGGACCGCCGGGGTTAACTCGGAGGAAGGTGGG<br>GATGACGTCAGATCATCATGCCCCTTACGTCCAGGGCTTCACGCATGCTACAATGGCCGGTACAACGGGATGCGACAT<br>GGTGACATGGAGCGGATCCCTTAAAACCGGTCTCAGTTCGGATCGTAGTCTGCAACTCGACTACGTGAAGGCGGAGTC<br>GCTAGTAATCGCGAATCAGCAACGTCGCGGTGAATGCGTTCCCGGGCCTTGTACACACCGCCCGTCAAGTCATGAAAG<br>TGGGCAGCACCCGAAGCCGGTGGCCTAACCCTTTTGGGATGGAGCCGTCTAAGGTGAGGCTCGTGATTGGGACTAAGT<br>CGTAACAAGGTAGCCGTACCGGAAGGTGCGGCTGGATCACCTCCTTT
	6119V5	TGGAGGGTTCGATTCTGGCTCAGGATGAACGCTGGCGGCGTGCTTAACACATGCAAGTCGAACGGGATCTAGTCAGCT<br>TGCTGACTGGTGAGAGTGGCGAACGGGTGAGTAATGCGTGACCAACCTGCCCCATGCTCCAGAATAGCTCTTGGAAAC<br>GGGTGGTAATGCTGGATGCTCCAACTTGACGCATGTCTTGTTGGGAAAGTGTTTAGTGGCATGGGATGGGGTCGCGTC<br>CTATCAGCTTGTAGGCGGGGTAATGGCCCACCTAGGCTTCGACGGGTAGCCGGCCTGAGAGGGCGGACGGCCACATT<br>GGGACTGAGATACGGCCCAGACTCCTACGGGAGGCAGCAGTGGGGAATATTGCGCAATGGGGGAAACCCTGACGCAG<br>CGACGCCGCGTGCGGGATGAAGGCCTTCGGGTTGTAAACCGCTTTTGATTGGGAGCAAGCTTTCGGGTGAGTGTACCT<br>TTCGAATAAGCGCCGGCTAACTACGTGCCAGCAGCCGCGGTAATACGTAGGGCGCAAGCGTTATCCGGAATTATTGGG<br>CGTAAAGAGCTTGTAGGCGGTTCGTCGCGTCTGGTGTGAAAGCCCATCGCTTAACGGTGGGTCTGCGCCGGGTACGGG<br>CGGGCTAGAGTGCAGTAGGGGAGACTGGAATTCCCGGTGTAACGGTGGAATGTGTAGATATCGGGAAGAACACCAAT<br>GGCGAAGGCAGGTCTCTGGGCTGTTACTGACGCTGAGAAGCGAAAGCGTGGGGAGCGAACAGGATTAGATACCCTGG<br>TAGTCCACGCCGTAAACGGTGGACGCTGGATGTGGGGCCCATTCCACGGGTTCCGTGTCGGAGCTAACGCGTTAAGCG<br>TCCCGCCTGGGGAGTACGGCCGCAAGGCTAAAACTCAAAGAAATTGACGGGGGCCCGCACAAGCGGCGGAGCATGCG<br>GATTAATTCGATGCAACGCGAAGAACCTTACCTGGGCTTGACATGTGCCTGACAGCTGCAGAGATGTGGTTTCCCTTC<br>GGGGCAGGTTCACAGGTGGTGCATGGTCGTCGTCAGCTCGTGTCGTGAGATGTTGGGTTAAGTCCCGCAACGAGCGCA<br>ACCCTCGCCCTGTGTTGCCAGCGGGTTATGCCGGGAACTCACGGGGGACCGCCGGGGTTAACTCGGAGGAAGGTGGG<br>GATGACGTCAGATCATCATGCCCCTTACGTCCAGGGCTTCACGCATGCTACAATGGCCGGTACAACGGGATGCGACAT<br>GGTGACATGGAGCGGATCCCTTAAAACCGGTCTCAGTTCGGATCGTAGTCTGCAACTCGACTACGTGAAGGCGGAGTC<br>GCTAGTAATCGCGAATCAGCAACGTCGCGGTGAATGCGTTCCCGGGCCTTGTACACACCGCCCGTCAAGTCATGAAAG<br>TGGGCAGCACCCGAAGCCGGTGGCCTAACCCTTTTGGGATGGAGCCGTCTAAGGTGAGGCTCGTGATTGGGACTAAGT<br>CGTAACAAGGTAGCCGTACCGGAAGGTGCGGCTGGATCACCTCCTTT
GS06	GED7760B	TGGAGGGTTCGATTCTGGCTCAGGATGAACGCTGGCGGCGTGCTTAACACATGCAAGTCGAACGGGATCCGACCAGCT<br>TGCTGGTTGGTGAGAGTGGCGAACGGGTGAGTAATGCGTGACCAACCTGCCCCATGCTCCAGAATAGCTCTTGGAAAC<br>GGGTGGTAATGCTGGATGCTCCAACTTGACGCATGTCTTGTTGGGAAAGTGTTTAGTGGCATGGGATGGGGTCGCGTC

		CTATCAGCTTGTAGGCGGGGTAATGGCCCACCTAGGCTTCGACGGGTAGCCGGCCTGAGAGGGCGGACGGCCACATT<br>GGGACTGAGATACGGCCCAGACTCCTACGGGAGGCAGCAGTGGGGAATATTGCGCAATGGGGGAAACCCTGACGCAG<br>CGACGCCGCGTGCGGGATGAAGGCCTTCGGGTTGTAAACCGCTTTTGATTGGGAGCAAGCTTTCGGGTGAGTGTACCT<br>TTCGAATAAGCGCCGGCTAACTACGTGCCAGCAGCCGCGGTAATACGTAGGGCGCAAGCGTTATCCGGAATTATTGGG<br>CGTAAAGAGCTTGTAGGCGGTTCGTCGCGTCTGGTGTGAAAGCCCATCGCTTAACGGTGGGTTTGCGCCGGGTACGGG<br>CGGGCTAGAGTGCAGTAGGGGAGACTGGAATTCCCGGTGTAACGGTGGAATGTGTAGATATCGGGAAGAACACCAAT<br>GGCGAAGGCAGGTCTCTGGGCTGTTACTGACGCTGAGAAGCGAAAGCGTGGGGAGCGAACAGGATTAGATACCCTGG<br>TAGTCCACGCCGTAAACGGTGGACGCTGGATGTGGGGCCCATTCCACGGGTTCTGTGTCGGAGCTAACGCGTTAAGCG<br>TCCCGCCTGGGGAGTACGGCCGCAAGGCTAAAACTCAAAGAAATTGACGGGGGCCCGCACAAGCGGCGGAGCATGCG<br>GATTAATTCGATGCAACGCGAAGAACCTTACCTGGGCTTGACATGTGCCTGACGACTGCAGAGATGTGGTTTCCCTTC<br>GGGGCAGGTTCACAGGTGGTGCATGGTCGTCGTCAGCTCGTGTCGTGAGATGTTGGGTTAAGTCCCGCAACGAGCGCA<br>ACCCTCGCCCTGTGTTGCCAGCGGGTTATGCCGGGAACTCACGGGGGACCGCCGGGGTTAACTCGGAGGAAGGTGGG<br>GATGACGTCAGATCATCATGCCCCTTACGTCCAGGGCTTCACGCATGCTACAATGGCCGGTACAACGGGATGCGACAT<br>GGTGACATGGAGCGGATCCCTTAAAACCGGTCTCAGTTCGGATCGTAGTCTGCAACTCGACTACGTGAAGGCGGAGTC<br>GCTAGTAATCGCGAATCAGCAACGTCGCGGTGAATGCGTTCCCGGGCCTTGTACACACCGCCCGTCAAGTCATGAAAG<br>TGGGCAGCACCCGAAGCCGGTGGCCTAACCCTTTTGGGATGGAGCCGTCTAAGGTGAGGCTCGTGATTGGGACTAAGT<br>CGTAACAAGGTAGCCGTACCGGAAGGTGCGGCTGGATCACCTCCTTT
GS07	CMW7778B	TGGAGGGTTCGATTCTGGCTCAGGATGAACGCTGGCGGCGTGCTTAACACATGCAAGTCGAACGGGATCTGACCAGCT<br>TGCTGGTTGGTGAGAGTGGCGAACGGGTGAGTAATGCGTGACCAACCTACCCTATGCTCCAGAATAGCTCCTGGAAAC<br>GGGTGGTAATGCTGGATGCTCCAACTTGACGCATGTTTTGTTGGGAAAGTGTTTAGCGGCATGGGATGGGGTCGCGTC<br>CTATCAGCTTGTAGGCGGGGTAATGGCCCACCTAGGCTTCGACGGGTAGCCGGCCTGAGAGGGCGGACGGCCACATT<br>GGGACTGAGATACGGCCCAGACTCCTACGGGAGGCAGCAGTGGGGAATATTGCGCAATGGGGGAAACCCTGACGCAG<br>CGACGCCGCGTGCGGGATGAAGGCCTTCGGGTTGTAAACCGCTTTTGATTGGGAGCAAGCCTTCGGGTGAGTGTACCT<br>TTCGAATAAGCGCCGGCTAACTACGTGCCAGCAGCCGCGGTAATACGTAGGGCGCAAGCGTTATCCGGATTTATTGGG<br>CGTAAAGAGCTTGTAGGCGGTTCGTCGCGTCTGGTGTGAAAGCCCATCGCTTAACGGTGGGTCTGCGCCGGGTACGGG<br>CGGGCTAGAGTGCAGTAGGGGAAACTGGAATTCTCGGTGTAACGGTGGAATGTGTAGATATCGGGAAGAACACCAAT<br>GGCGAAGGCAGGTTTCTGGGCTGTTACTGACGCTGAGAAGCGAAAGCGTGGGGAGCGAACAGGATTAGATACCCTGG<br>TAGTCCACGCCGTAAACGGTGGACGCTGGATGTGGGGCCCATTCCACGGGTTCCGTGTCGGAGCTAACGCGTTAAGCG<br>TCCCGCCTGGGGAGTACGGCCGCAAGGCTAAAACTCAAAGAAATTGACGGGGGCCCGCACAAGCGGCGGAGCATGCG<br>GATTAATTCGATGCAACGCGAAGAACCTTACCTGGGCTTGACATGTGCCTGATGACTGCAGAGATGTGGTTTCCTTTC<br>GGGGCAGGTTCACAGGTGGTGCATGGTCGTCGTCAGCTCGTGTCGTGAGATGTTGGGTTAAGTCCCGCAACGAGCGCA<br>ACCCTCGCCCTGTGTTGCCAGCGGGTTATGCCGGGAACTCACGGGGGACCGCCGGGGTTAACTCGGAGGAAGGTGGG<br>GATGACGTCAGATCATCATGCCCCTTACGTCCAGGGCTTCACGCATGCTACAATGGCCGGTACAACGGGGTGCGACAT<br>GGTGACATGGAGCTAATCCCTTAAAACCGGTCTCAGTTCGGATCGTAGTCTGCAACTCGACTACGTGAAGGCGGAGTC

		GCTAGTAATCGCGAATCAGCAACGTCGCGGTGAATGCGTTCCCGGGCCTTGTACACACCGCCCGTCAAGTCATGAAAG<br>TGGGCAGCACCCGAAGCCGGTGGCCTGACCTTTTGGAGGGAGCCGTCTAAGGTGAGGCTCGTGATTGGGACTAAGTC<br>GTAACAAGGTAGCCGTACCGGAAGGTGCGGCTGGATCACCTCCTTT
GS08	KA0025	TGGAGGGTTCGATTCTGGCTCAGGATGAACGCTGGCGGCGTGCTTAACACATGCAAGTCGAACGGGATCTGACCAGCT<br>TGCTGGTTGGTGAGAGTGGCGAACGGGTGAGTAATGCGTGACCAACCTACCCCATGCTCCAGAATAGCTCCTGGAAAC<br>GGGTGGTAATGCTGGATGCTCCAACTTGACGCATGTTTTGTTGGGAAAGTGTTTAGCGGCATGGGATGGGGTCGCGTC<br>CTATCAGCTTGTAGGCGGGGTAATGGCCCACCTAGGCTTCGACGGGTAGCCGGCCTGAGAGGGCGGACGGCCACATT<br>GGGACTGAGATACGGCCCAGACTCCTACGGGAGGCAGCAGTGGGGAATATTGCGCAATGGGGGAAACCCTGACGCAG<br>CGACGCCGCGTGCGGGATGAAGGCCTTCGGGTTGTAAACCGCTTTTGATTGGGAGCAAGCTTTCGGGTGAGTGTACCT<br>TTCGAATAAGCGCCGGCTAACTACGTGCCAGCAGCCGCGGTAATACGTAGGGCGCAAGCGTTATCCGGAATTATTGGG<br>CGTAAAGAGCTTGTAGGCGGTTCGTCGCGTCTGGTGTGAAAGCCCATCGCTTAACGGTGGGTCTGCGCCGGGTACGGG<br>CGGGCTAGAGTGCAGTAGGGGAAACTGGAATTCTCGGTGTAACGGTGGAATGTGTAGATATCGGGAAGAACACCAAT<br>GGCGAAGGCAGGTTTCTGGGCTGTTACTGACGCTGAGAAGCGAAAGCGTGGGGAGCGAACAGGATTAGATACCCTGG<br>TAGTCCACGCCGTAAACGGTGGACGCTGGATGTGGGGCCCATTCCACGGGTTCCGTGTCGGAGCTAACGCGTTAAGCG<br>TCCCGCCTGGGGAGTACGGCCGCAAGGCTAAAACTCAAAGAAATTGACGGGGGCCCGCACAAGCGGCGGAGCATGCG<br>GATTAATTCGATGCAACGCGAAGAACCTTACCTGGGCTTGACATGTGCCTGACGACTGCAGAGATGTGGTTTCCTTTC<br>GGGGCAGGTTCACAGGTGGTGCATGGTCGTCGTCAGCTCGTGTCGTGAGATGTTGGGTTAAGTCCCGCAACGAGCGCA<br>ACCCTCGCCCTGTGTTGCCAGCGGGTTATGCCGGGAACTCACGGGGGACCGCCGGGGTTAACTCGGAGGAAGGTGGG<br>GATGACGTCAGATCATCATGCCCCTTACGTCCAGGGCTTCACGCATGCTACAATGGCCAGTACAACGGGTTGCGACAT<br>GGTGACATGGAGCTAATCCCTTAAAACTGGTCTCAGTTCGGATCGTAGTCTGCAACTCGACTACGTGAAGGCGGAGTC<br>GCTAGTAATCGCGAATCAGCAACGTCGCGGTGAATGCGTTCCCGGGCCTTGTACACACCGCCCGTCAAGTCATGAAAG<br>TGGGCAGCACCCGAAGCCGGTGGCCTAACCCTTTTGGGATGGAGCCGTCTAAGGTGAGGCTCGTGATTGGGACTAAGT<br>CGTAACAAGGTAGCCGTACCGGAAGGTGCGGCTGGATCACCTCCTTT
GS		NR010TGGAGGGTTCGATTCTGGCTCAGGATGAACGCTGGCGGCGTGCTTAACACATGCAAGTCGAACGGGATCTGAC<br>CAGCTTGCTGGTTGGTGAGAGTGGCGAACGGGTGAGTAATGCGTGACCAACCTGCCCCATGCTCCAGAATAGCTCTTG<br>GAAACGGGTGGTAATGCTGGATGCTCCAACTTGACGCATGTCTTGTTGGGAAAGTGTTTAGTGGCATGGGATGGGGTC<br>GCGTCCTATCAGCTTGTAGGCGGGGTAATGGCCCACCTAGGCTTCGACGGGTAGCCGGCCTGAGAGGGCGGACGGCC<br>ACATTGGGACTGAGATACGGCCCAGACTCCTACGGGAGGCAGCAGTGGGGAATATTGCGCAATGGGGGAAACCCTGA<br>CGCAGCGACGCCGCGTGCGGGATGAAGGCCTTCGGGTTGTAAACCGCTTTTGATTGGGAGCAAGCTTTCGGGTGAGTG<br>TACCTTTCGAATAAGCGCCGGCTAACTACGTGCCAGCAGCCGCGGTAATACGTAGGGCGCAAGCGTTATCCGGAATTA<br>TTGGGCGTAAAGAGCTTGTAGGCGGTTCGTCGCGTCTGGTGTGAAAGCCCATCGCTTAACGGTGGGTCTGCGCCGGGT<br>ACGGGCGGGCTAGAGTGCAGTAGGGGAGACTGGAATTCCCGGTGTAACGGTGGAATGTGTAGATATCGGGAAGAACA<br>CCAATGGCGAAGGCAGGTCTCTGGGCTGTTACTGACGCTGAGAAGCGAAAGCGTGGGGAGCGAACAGGATTAGATAC<br>CCTGGTAGTCCACGCCGTAAACGGTGGACGCTGGATGTGGGGCCCATTCCACGGGTTCCGTGTCGGAGCTAACGCGTT

		AAGCGTCCCGCCTGGGGAGTACGGCCGCAAGGCTAAAACTCAAAGAAATTGACGGGGGCCCGCACAAGCGGCGGAG<br>CATGCGGATTAATTCGATGCAACGCGAAGAACCTTACCTGGGCTTGACATGTGCCTGTCGACTGCAGAGATGTGGTTT<br>CCCTTCGGGGCAGGTTCACAGGTGGTGCATGGTCGTCGTCAGCTCGTGTCGTGAGATGTTGGGTTAAGTCCCGCAACG<br>AGCGCAACCCTTGCCCTGTGTTGCCAGCGGGTTATGCCGGGAACTCACGGGGGACCGCCGGGGTTAACTCGGAGGAA<br>GGTGGGGATGACGTCAGATCATCATGCCCCTTACGTCCAGGGCTTCACGCATGCTACAATGGCCAGTACAACGGGTTG<br>CGACATGGTGACATGGAGCTAATCCCTTAAAACTGGTCTCAGTTCGGATCGTAGTCTGCAACTCGACTACGTGAAGGC<br>GGAGTCGCTAGTAATCGCGAATCAGCAACGTCGCGGTGAATGCGTTCCCGGGCCTTGTACACACCGCCCGTCAAGTCA<br>TGAAAGTGGGCAGCACCCGAAGCCGGTGGCCTGACCTTTTTGGAGGGAGCCGTCTAAGGTGAGGCTCGTGATTGGGA<br>CTAAGTCGTAACAAGGTAGCCGTACCGGAAGGTGCGGCTGGATCACCTCCTTT
